# Mechanism of Action of Zinc Oxide Nanoparticles as an Antibacterial Agent Against *Streptococcus mutans*

**DOI:** 10.3390/biom15121660

**Published:** 2025-11-27

**Authors:** Raphaelle Emram, Ronit Vogt Sionov, Vitaly Gutkin, Asaf Wilensky, Doron Steinberg, Rawi Assad

**Affiliations:** 1 Faculty of Dental Medicine, Institute of Biomedical and Oral Research (IBOR), The Hebrew University of Jerusalem, Ein Kerem Campus, Jerusalem 9112102, Israel; raphaell.emram@mail.huji.ac.il (R.E.); dorons@ekmd.huji.ac.il (D.S.); 2 Department of Periodontology, Hadassah Medical Center, Faculty of Dental Medicine, The Hebrew University of Jerusalem, Jerusalem 9112102, Israel; asafw@ekmd.huji.ac.il; 3 Unit for Nano Characterization, The Harvey M. Krueger Family Center for Nanoscience and Nanotechnology, The Hebrew University of Jerusalem, Edmond J. Safra Campus, Jerusalem 9190401, Israel; vitalyg@savion.huji.ac.il

**Keywords:** antibacterial, anti-biofilm, *Streptococcus mutans*, ZnO nanoparticles

## Abstract

Oral microbial biofilms play a critical role in the development of various oral infectious diseases, including periodontitis and tooth caries, with *Streptococcus mutans* recognized as a key biofilm-forming bacterium due to its strong adhesion and acidogenic capacity. Zinc oxide nanoparticles (ZnO NPs) have demonstrated antibacterial properties against various bacteria. This study investigated the antibacterial and antibiofilm properties of ZnO NPs on *S. mutans* and elucidated their mode of action. Bacterial cultures were exposed to increasing concentrations of ZnO NPs, and planktonic growth, biofilm biomass and biofilm metabolic activity were measured. Complementary assays assessed bacterial ATP content, pH shift in the media, reactive oxygen species (ROS) production, membrane integrity (SYTO 9/PI live/dead staining) and membrane potential. Morphological changes were examined by high-resolution scanning electron microscopy (HR-SEM), while gene expression was analyzed by real-time qPCR. We observed that ZnO NPs inhibited *S. mutans* growth and biofilm formation in a dose-dependent manner, with both the minimum inhibitory and biofilm inhibitory concentration of 0.5 mg/mL. ZnO NP treatment disrupted bacterial membranes, caused cytoplasmic leakage, and induced ROS production. EPS production determined by Congo Red staining was significantly reduced. Gene expression analysis revealed significant upregulation of *vicR*, *luxS*, *wapA*, *gtpB*, *nox* and *ftsZ*, and downregulation of *spaP*, *gtpC* and *atpB*. In conclusion, ZnO NPs compromise *S. mutans* viability and biofilm development through oxidative stress and membrane disruption, highlighting their potential use as bioactive materials in oral healthcare.

## 1. Introduction

Dental biofilms are complex microbial communities that play a central role in the onset and progression of oral diseases such as dental caries and periodontitis [1]. Among the numerous bacterial species inhabiting the oral cavity, *Streptococcus mutans* is particularly notable for its robust biofilm-forming capability and resilience in acidic environments [2,3]. This Gram-positive, facultative anaerobic bacterium adheres to hard surfaces and ferments dietary carbohydrates, producing acids that demineralize enamel. *S. mutans* has been found in biofilms developing on both tooth surfaces [4] and titanium implants [5], demonstrating its ability to colonize diverse oral substrates. In addition to its established role in dental caries [3], *S. mutans* has been detected as an early colonizer in developing dental biofilms associated with periodontitis and peri-implantitis, suggesting its involvement in the initial stages of dysbiotic biofilm formation and disease progression [6,7]. These features underscore its significance not only as a cariogenic pathogen but also as a potential initiator in broader processes of oral biofilm maturation and imbalance.

A key factor contributing to the virulence of *S. mutans* is its ability to produce extracellular polysaccharides (EPS), which form a structural matrix that facilitates bacterial adhesion and stabilizes biofilm architecture [3,8,9]. This EPS-rich biofilm environment supports the accumulation and integration of other microbial species and also enhances bacterial resistance to antimicrobials and host defense mechanisms [10,11,12], presenting a challenge for maintaining a healthy oral cavity [13].

Given the central role of *S. mutans* in shaping pathogenic oral biofilms, strategies that target its growth, adhesion, and survival have been at the forefront of dental research. Traditional approaches, such as mechanical debridement or the use of broad-spectrum antimicrobials, often face limitations, including incomplete biofilm removal and the potential for bacterial resistance. These challenges have driven growing interest in nanomaterials as novel tools to prevent or disrupt EPS production and biofilm formation [14,15,16].

Zinc oxide (ZnO) in the form of nanoparticles is a widely studied metal oxide with recognized antimicrobial and antibiofilm properties against both antibiotic-sensitive and antibiotic-resistant bacterial strains, including *Enterococcus faecalis*, *Staphylococcus aureus*, *Escherichia coli*, *Acinetobacter baumannii*, and *Pseudomonas aeruginosa* [17,18,19,20,21,22,23,24,25,26,27,28,29]. Its mechanisms of antibacterial action on these bacteria were found to be multifaceted, including the disruption of bacterial cell membranes [22,23], generation of reactive oxygen species (ROS) [23,24,25], lipid peroxidation [23] and release of zinc ions (Zn^2+^) [26,27], all of which impair bacterial growth and metabolic activity. Because of this multi-targeted activity, ZnO acts through multiple pathways simultaneously, reducing the likelihood of resistance development [28], although transient adaptive resistance has been reported [29].

There are reports demonstrating that ZnO NPs can reduce *S. mutans* growth and biofilm formation [30,31,32,33,34,35]. The underlying mechanisms of action against this bacterium are still poorly understood. The few studies that have explored potential mechanisms of action focused mainly on single endpoints as EPS suppression and protein leakage [34], biofilm disruption linked to oxidative stress pathways [36] or altered protein expression in *S. mutans* [37]. A broad, multi-parameter analysis of *S. mutans* responses to pure ZnO NPs is still needed. Therefore, the present work aims to further elucidate the mode of action of ZnO NPs against *S. mutans* by integrating physiological, structural, and transcriptional analyses.

ZnO NPs are widely used in clinical use in dentistry [38,39,40], where it has been explored as an additive to restorative materials [41], sealants [42], and cements [30], to provide antibacterial activity without compromising material performance.

Based on previous studies demonstrating the antibacterial and antibiofilm activities of zinc oxide nanoparticles (ZnO NPs) against *Streptococcus mutans* [43,44], the objective of the present study was to further elucidate the underlying biological mechanisms governing ZnO–*S. mutans* interactions. Building upon these important and valuable contributions, we employed a multi-faceted methodological approach to provide a comprehensive understanding of ZnO’s antimicrobial properties, its impact on biofilm formation, and its mode of action.

In this study, ZnO was applied in nanoparticle form to maximize surface reactivity and enhance bacterial interaction. The findings reveal that ZnO NPs suppress *S. mutans* growth and biofilm development through multiple pathways, including oxidative stress induction, disruption of cell membranes, impairment of energy metabolism, and modulation of virulence- and stress-related gene expression. This integrative mechanistic perspective advances current knowledge by providing deeper insight into the multifaceted antibacterial activity of ZnO NPs and reinforces their potential as bioactive materials in preventive and restorative dentistry.

## 2. Materials and Methods

### 2.1. Materials

Zinc oxide (ZnO) nanoparticles (<100 nm particle size) in a 20% suspension in water was purchased from Sigma Aldrich (Cat. No. 721077; St. Louis, MO, USA). The average particle size is stated to be ≤40 nm according to the Certificate of Analysis (Supplementary Data S1).

### 2.2. Characterization of ZnO NPs by DLS, SEM and STEM

Dynamic Light Scattering (DLS) measurements were performed using a Zetasizer Nano ZS (Malvern Panalytical Ltd., Mavern, UK). ZnO nanoparticles were dispersed either in double-distilled water (DDW) or in Brain Heart Infusion (BHI) medium and transferred into standard disposable cuvettes suitable for DLS analysis. Measurements were acquired in backscatter mode at a detection angle of 173° (NIBS) at 25 °C. The hydrodynamic diameter (Z-average) was calculated from the autocorrelation function of the scattered light assuming spherical particle geometry, as implemented by the instrument software [45]. This analysis showed that the Z-average of the ZnO NPs was 130–147 nm and the Polydispersity index (PI) was 0.175–0.236 (Supplementary Data S2E).

Electron microscopy imaging was performed by Analytical High Resolution Scanning Electron Microscope Apreo 2S (Thermo Fisher Scientific, Eindhoven, The Netherlands) using the SEM mode (accelerating voltage 10–15 kV and current 0.2 nA). This analysis showed that the average particle diameter was 10–47 nm (Supplementary Data S2A,B).

The high-resolution TEM images were performed using the Aberration Probe-Corrected Scanning Transmission Electron Microscope Themis Z G3 (Thermo Fisher Scientific, Eindhoven, The Netherlands), accelerating voltage 300 kV and current 0.2 nA, showing an average particle diameter of 10–30 nm (Supplementary Data S2C,D).

### 2.3. Bacteria and Cultivation

The cariogenic *Streptococcus mutans* UA159 strain (ATCC 700610) was used as the bacterial model strain in our study. It was cultured in brain heart infusion (BHI) broth (HiMedia Laboratories Pvt. Ltd., Maharashtra, India), for planktonic-growing conditions, and supplemented with 2% sucrose for biofilm-producing conditions. A starter culture was prepared the day before the experiment by inoculating 100 μL of a frozen stock into 10 mL of BHI, followed by overnight incubation at 37 °C in a humidified incubator supplemented with 5% CO_2_ [46]. All experiments were performed under these incubation conditions.

### 2.4. MTT Metabolic Assay of Biofilms and CV Biofilm Biomass Staining

To determine the biofilm formation of *S. mutans*, an overnight culture was diluted in BHI supplemented with 2% sucrose (BHIS) to an initial OD_600nm_ of 0.2, and 100 μL of this suspension was added to 100 μL of the various two-fold concentrations of ZnO NPs in BHIS in flat-bottomed 96-well tissue-grade plates (Corning, Kennebunk, ME, USA), yielding an initial bacterial OD_600nm_ of 0.1 and a ×1 agent concentration. After a 24 h incubation, the biofilms formed were washed twice with 200 μL PBS and then either exposed to 50 μL of 0.5 mg/mL MTT (Sigma) [47] in PBS for 1 h at 37 °C to measure the metabolic activity or 100 μL of a 0.25% crystal violet (CV) solution (Merck KGaH, Darmstadt, Germany) for 20 min at room temperature to measure the biofilm biomass [48].

MTT assay: At the end of incubation, 180 μL PBS was added to the MTT-exposed biofilms, and the supernatant was decanted. The tetrazolium formed in the biofilms was dissolved in 200 μL dimethyl sulfoxide (DMSO), and the absorbance at 570 nm was measured in a Multiskan SkyHigh microplate reader (Thermo Scientific Multiskan SkyHigh, Life Technologies, Holdings Pte Ltd., Singapore).

CV assay: The CV-stained biofilms were washed several times with DDW to remove excess stain, and the CV stain of biofilms was dissolved in 200 μL of 33% acetic acid, and the absorbance at 595 nm was measured in a Multiskan SkyHigh microplate reader.

Triplicates were performed for each experiment that was repeated three times. The percentage of metabolically active bacteria in biofilms and the biofilm biomass of the treated samples were calculated in comparison to control biofilms after subtracting the turbidity caused by ZnO NPs itself or the broth medium alone without bacteria.

### 2.5. Colony Forming Units (CFU)

To determine the bacterial count in control and ZnO NP-treated samples with an initial OD_600nm_ of 0.1, ten-fold serial dilutions were prepared in BHI medium. A 100 µL aliquot from each dilution was spread onto BHI agar plates and incubated overnight at 37 °C in a humidified incubator supplemented with 5% CO_2_. Following incubation, the number of colonies on each plate was counted manually, and the colony-forming units (CFU) per well were calculated using the formula [49]:

CFU = number of colonies × dilution factor × original sample volume × 10

The multiplication factor of 10 was applied in the calculation to account for the fact that only 1/10 of the volume from each dilution was plated on the agar.

### 2.6. ATP Content

Another assay used to determine the viability of bacteria was the BacTiter-Glo microbial cell viability reagent (Promega, Madison, WI, USA) which quantifies ATP content as an indicator of metabolically active cells [46]. At designated time points, 100 μL aliquots were taken from bacterial cultures grown in 5 mL medium with an initial OD_600nm_ of 0.1 and transferred to a µ-clear 96-well flat-bottom white microplate (Greiner Bio-One GmbH, Frickenhausen, Germany). An equal volume (100 µL) of BacTiter-Glo reagent was then added to each well. Bacteria-free medium with or without ZnO NPs served as background reads. ZnO NPs did not interfere with the assay. Following a 10 min incubation at room temperature, luminescence was measured in an M200 infinite plate reader (Tecan Group Ltd., Männedorf, Switzerland).

### 2.7. pH Measurements

Bacterial suspensions of initial OD_600nm_ = 0.1 in BHI medium were treated with different concentrations of ZnO NPs (0.5–1 mg/mL) and incubated at 37 °C in a humidified incubator supplemented with 5% CO_2_ for 24 h. At various time points, the pH of the samples was measured using pH indicator paper strips (MColorpHast, Merck KGaA, Darmstadt, Germany).

### 2.8. SYTO 9/PI Live/Dead Staining

Following a 2 h exposure of *S. mutans* (initial OD_600nm_ = 0.3) to ZnO NPs, the bacteria were stained with 3.3 µM SYTO 9 (Molecular Probes, Life Technologies, Carlsbad, CA, USA) and 10 µg/mL propidium iodide (PI) (Sigma, St. Louis, MO, USA) in 1 mL PBS for 20 min. Green and red fluorescence were subsequently measured by flow cytometry (LSR Fortessa, BD Biosciences, San Jose, CA, USA) using excitation/emission settings of 488/520 nm for SYTO 9 and 561/586 nm for PI, respectively [50]. SYTO 9 binds to nucleic acids in both live and dead bacterial cells, emitting green fluorescence, whereas PI penetrates only membrane-compromised cells and emits red fluorescence upon nucleic acid binding. Notably, dead bacteria that have lost nucleic acid content due to cytoplasmic leakage appear as SYTO 9 low/negative and PI-negative cells.

### 2.9. Morphological Imaging by High-Resolution Scanning Electron Microscopy (HR-SEM)

To visualize the morphology of planktonically growing cells following a 2 h exposure to ZnO NPs, bacterial cultures with an initial OD_600nm_ of 0.3 were rinsed once with DDW and fixed in 4% glutaraldehyde (Electron Microscopy Sciences, Hatfield, PA, USA) for 2 h [51]. Thereafter, the fixed cells were washed once with ddw and resuspended in 50 µL DDW. Ten µL aliquots were placed onto 0.7 × 0.7 cm glass fragments prepared from microscope slides and air-dried. The dried specimens were sputter-coated with iridium and examined using an Analytical High Resolution Scanning Electron Microscope Apreo 2S LoVac (Thermo Fisher Scientific) at various magnifications.

### 2.10. Energy Dispersive X-Ray Spectroscopy (EDS)

The elemental analysis was measured with the EDS (Energy Dispersive X-Ray Spectroscopy) system composed of an UltraDry Premium 60 mm^2^ Silicon Drift (SSD) detector by Thermo Fisher Scientific. Accelerating voltage 15 kV and current 1.6 nA.

### 2.11. Determination of Extracellular Polysaccharide (EPS) Production by Congo Red

The Congo Red agar method [52] was used to evaluate EPS production by *S. mutans* (initial OD_600nm_ = 0.1) that had been incubated for 2 h, 4 h and 6 h in BHI medium in the absence and presence of 0.1, 0.25, 0.5, 1, 2.5, 5 and 10 mg/mL ZnO NPs. At each time points, 10 µL of control and ZnO NP-treated *S. mutans* cultures were spotted on BHI agar supplemented with 0.08% Congo Red and 1% sucrose in flat-bottomed six-well plates (Corning) followed by a 24 h incubation at 37 °C in a humidified incubator supplemented with 5% CO_2_. The area of the black halo that appears around the bacterial colonies, which presents EPS production, was analyzed using the ImageJ 1.53e software (The National Institute of Health, Bethesda, MD, USA).

The Congo Red agar plates were prepared by diluting 10 mL of an autoclaved 0.8% stock solution of Congo Red stain in 100 mL of autoclaved BHI containing 1.5% agar to which sucrose was added to a final concentration of 1%, each well of the 6-well plates receiving 2 mL of the solution.

### 2.12. ROS Production

Reactive oxygen species (ROS) production by *S. mutans* was quantified using a luminol-dependent chemiluminescence assay [53]. An overnight culture of *S. mutans* was adjusted to an OD_600nm_ of 0.2 in Hank’s balanced salt solution (HBSS; without phenol red) supplemented with 1% D-glucose. Each well of a 96-well white plate contained 100 µL of the bacterial suspension and 100 µL of ZnO NP suspension prepared in HBSS. Final ZnO NP concentrations ranged from 0 to 1 mg/mL. Luminol (50 µM final concentration; prepared from a 50 mM stock solution in DMSO) and horseradish peroxidase (HRP; 4 U/mL; Sigma) were included in all wells. Bacteria-free control samples with or without ZnO NPs were included to assess background signals. Chemiluminescence was recorded in an M200 infinite Tecan plate reader with 1 s integration time per well, every 10 s for up to 2 h. Results are expressed as relative luminescence units (RLU) over time to monitor the kinetics of ROS production.

### 2.13. Membrane Potential Determination by Flow Cytometry

The effect of ZnO NPs on the membrane potential of *S. mutans* was assessed using the BacLight Membrane Potential Kit (Molecular Probes, Life Technologies, Eugene, OR, USA) according to the manufacturer’s instructions [50]. An overnight culture of *S. mutans* in BHI was diluted to an OD_600nm_ of 0.3 and exposed to different concentrations of ZnO NPs for 30 min. Cells were then centrifuged and resuspended in 1 mL PBS and stained with the potentiometric dye 3,3′-diethyloxacarbocyanine iodide (DiOC2(3)) at a final concentration of 30 µM and incubated for 20 min at room temperature. The fluorescence intensities were measured by flow cytometry (LSR-Fortessa, BD Biosciences) using a 488 nm excitation laser, with green (530 nm) and red (610/620 nm) filters. A total of 50,000 events were recorded for each sample. Data acquisition was performed with BD FACSDiva software, and analysis was carried out using FCS Express 7 (De Novo Software). All treatments were performed in triplicate.

For each sample, the geometric mean fluorescence intensity of both green and red channels was calculated. The geometric mean fluorescence intensity of the untreated control was set to 1, and the fluorescence values of all treated samples were normalized relative to the controls. Relative fluorescence ratios (red/green) were used to assess membrane potential changes, where a relative increase in red fluorescence compared to green fluorescence indicates membrane hyperpolarization.

### 2.14. RNA Extraction

An overnight culture of *S. mutans* was diluted to an initial OD_600nm_ of 0.1 in BHI supplemented with 2% sucrose and incubated for 2 h in the absence or presence of ZnO NPs at a final concentration of 0.25 mg/mL. Following incubation, bacterial pellets were treated with 1 mL of RNAprotect (Qiagen, Hilden, Germany) for 5 min on ice. The pellets were then resuspended in 1 mL of Tri-Reagent (Sigma-Aldrich) and transferred to Type B bead tubes (Macherey-Nagel, Düren, Germany). Cell disruption was carried out using a FastPrep cell disrupter (BIO 101, Savant Instruments, Holbrook, NY, USA) at 4.5 m/s for three cycles of 45 s, with 5 min cooling intervals on ice between each cycle. After lysis, the glass beads were removed by centrifugation at 20,000× *g* for 2 min. The supernatant was mixed with 200 µL of chloroform, vortexed vigorously for 15 s, and incubated at room temperature for 15 min to allow phase separation. The samples were then centrifuged at 21,100× *g* for 15 min at 4 °C. The aqueous phase containing RNA was carefully collected, mixed with an equal volume of isopropanol, and incubated at room temperature for 30 min. RNA was precipitated by centrifugation at 21,100× *g* for 30 min. The resulting RNA pellets were washed twice with 1 mL of 75% ethanol, air-dried for 30 min, and resuspended in 20 μL RNase- and DNase-free water (Bio-Lab., Jerusalem, Israel). RNA purity was assessed by electrophoresis on a 1% agarose gel containing ethidium bromide, and RNA concentration was determined using a Nanodrop spectrophotometer (Nanovue, GE Healthcare Life Sciences, Buckinghamshire, UK) [50].

### 2.15. Reverse Transcription (RT) and Quantitative Real-Time PCR

cDNA was synthesized from RNA using the AB High-Capacity cDNA Reverse Transcription Kit (Applied Biosciences by Thermo Fisher Scientific, Vilnius, Lithuania). Quantitative real-time PCR (qPCR) was performed using the Bio-Rad CFX Connect Real-Time System and the CFX Maestro software. Each reaction contained 10 ng of cDNA, Power SYBR Green PCR Master Mix (Applied Biosystems, Life Technologies, Woolston Warrington, UK), and 300 nM of the respective forward and reverse primers (Table 1). Each sample was run in triplicate for each gene, and the average Ct obtained was used for the calculations. The thermal cycling conditions consisted of an initial incubation at 50 °C for 2 min, followed by a polymerase activation step at 95 °C for 10 min. Amplification was carried out for 40 cycles of 95 °C for 15 s and 60 °C for 1 min. Melt curve analysis was performed by heating to 95 °C for 15 s, cooling to 60 °C for 10 s, and then increasing the temperature in 0.5 °C increments until reaching 95 °C. The housekeeping gene *gyrA* [54] was used as the internal reference, and relative gene expression was calculated using the 2^−ΔΔCt^ method. Fold changes in expression of the genes were determined by comparing their expression in the treated samples with their respective controls, with control expression normalized to 1 for each gene. The ΔCt of each of the three treated samples were compared to the ΔCt of each of the three controls. All experiments were conducted in triplicate and repeated three times independently.

### 2.16. Cytotoxicity Assay

To study the cytotoxicity of ZnO NPs, 4 × 10^4^ Vero epithelial cells (CCL-81, ATCC) and 4 × 10^4^ HEK-293T cells were seeded in 200 µL of DMEM supplemented with 10% heat-inactivated fetal bovine serum (Sigma, St. Louis, MO, USA) in 96-flat-bottomed tissue-grade plates for a 24 h incubation. The following day, the medium was exchanged with medium containing different concentrations of ZnO NPs (0–10 mg/mL) for another 24 h incubation. At the end of incubation, MTT was added to a final concentration of 1 mg/mL, to measure the metabolic activity of the cells [46]. The formazan formed was dissolved in 200 µL dimethylsulfoxide, and the OD at 570 was measured in a Multiskan SkyHigh microplate reader (Thermo Scientific Multiskan SkyHigh, Life Technologies, Holdings Pte Ltd., Singapore).

### 2.17. Statistical Analysis

The experiments were performed in triplicate. The data is presented as the average ± standard deviation. Statistical significance was calculated according to the Student’s *t*-test. A *p* value less than 0.05 was considered statistically significant when comparing between groups.

## 3. Results

### 3.1. Antibacterial and Anti-Biofilm Effects of ZnO NPs on S. mutans

**Figure 1:**
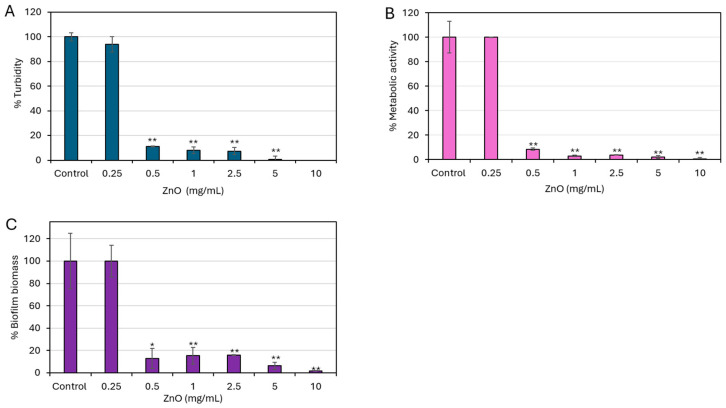
ZnO NPs prevent the planktonic growth and biofilm formation of *S. mutans*. (**A**) Planktonic growth of *S. mutans* following a 24 h incubation with indicated ZnO NP concentrations. (**B**) Metabolic activity of biofilms formed after a 24 h incubation with ZnO NPs as determined by MTT assay. (**C**) Biofilm biomass after a 24 h incubation with ZnO NPs as determined by crystal violet staining. The control bacteria incubated for 24 h in the absence of ZnO NPs was set to 100%. Data are presented as mean ± SD of triplicate experiments. * *p* < 0.05 and ** *p* < 0.005 compared to control.

To establish the minimum inhibitory concentration (MIC) and minimum biofilm inhibitory concentration (MBIC) of ZnO NPs, the bacteria were exposed to a range of ZnO NP concentrations (0.1–10 mg/mL) under conditions favoring either planktonic growth or biofilm formation. ZnO NPs exhibited a concentration-dependent antibacterial effect against *S. mutans* UA159 (Figure 1A). Significant reduction in bacterial growth was observed at 0.5 mg/mL ZnO NPs, with turbidity decreasing to 10% of control levels (*p* < 0.005) (Figure 1A). Higher concentrations of ZnO NPs (1–10 mg/mL) resulted in even stronger growth inhibition, reaching 92–100% inhibition (Figure 1A). Based on these findings, 0.5 mg/mL ZnO NPs is considered the MIC value for this bacterial strain.

A similar inhibitory effect of ZnO NPs on biofilm formation was observed (Figure 1B,C). The MTT assay showed a significant reduction (91.5 ± 0.9% reduction) in biofilm metabolic activity at ZnO NP concentrations equal to and above 0.5 mg/mL (*p* < 0.005) (Figure 1B), whereas no significant alteration was detected at 0.25 mg/mL (Figure 1B), indicating that ZnO NPs impair bacterial biofilm formation in a concentration-dependent manner. Consistently, biofilm formation assessed by crystal violet staining showed decreased biofilm biomass with increasing ZnO NP concentrations (Figure 1C). A significant decrease of >90% was observed at 0.5 mg/mL (*p* < 0.005), reaching 98 ± 0.07% inhibition at 10 mg/mL (Figure 1C). These findings indicate that the minimum biofilm inhibitory concentration (MBIC) value of ZnO NPs is 0.5 mg/mL, aligning with the MIC determined under planktonic growth conditions.

### 3.2. ZnO NPs Exert a Bacteriostatic Effect That Progresses into a Bactericidal Effect upon Prolonged Exposure

**Figure 2:**
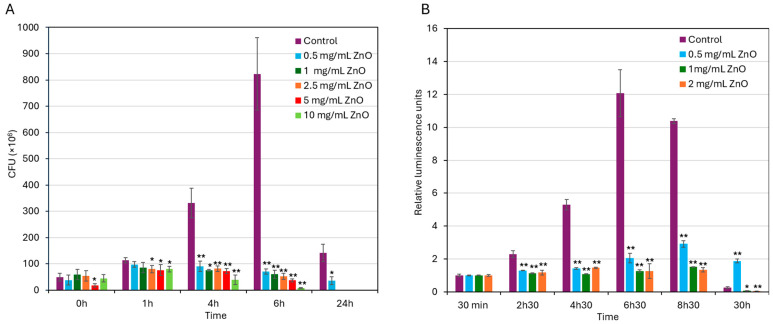
Bacteriostatic and bactericidal effect of ZnO NPs on *S. mutans*. (**A**) CFU counts of *S. mutans* at various time points after exposure to indicated concentrations of ZnO NPs. (**B**) ATP levels in *S. mutans* at various time points after being exposed to the indicated concentrations of ZnO NPs, relative to the initial levels at time 0 which was set to 1. Data are presented as mean ± SD of triplicate experiments. * *p* < 0.05 and ** *p* < 0.005 compared to control bacteria at each time point.

To further validate the antibacterial activity of ZnO NPs against *S. mutans* UA159, colony-forming units (CFUs) were counted at various time points following bacterial exposure to different concentrations of ZnO NPs (0–10 mg/mL). As expected, the CFU of untreated control bacteria increased significantly during the first 6 h of incubation consistent with active bacterial proliferation (Figure 2A), followed by a notable decline after 24 h when the bacteria have entered the dead phase. In contrast, treatment with ZnO NPs at concentrations of 0.5–2 mg/mL effectively suppressed bacterial growth with more and less stable CFU counts during the first 6 h of incubation, followed by a decline in CFU counts after 24 h (Figure 2A). At concentrations of 1 mg/mL and above, no colonies were detected after a 24 h incubation (Figure 2A). These findings indicate that ZnO NPs at 0.5 mg/mL and above have an initial bacteriostatic effect on the bacteria, which progress to a bactericidal effect with prolonged exposure.

Additionally, relative changes in intracellular ATP levels were measured over time using the BacTiter-Glo microbial viability kit (Promega, Madison, WI, USA) to further confirm the antibacterial activity of ZnO NPs. This assay measures ATP levels, which serve as a measure for metabolically active bacterial cells. As shown in Figure 2B, untreated control bacteria exhibited a marked increase in ATP levels during the first 6.5 h, consistent with active bacterial proliferation until reaching the stationary growth phase (6.5–8.5 h). By 30 h, there was a drop in ATP levels in control bacteria which reflects the transition into late stationary phase/early dead phase (Figure 2B). Bacteria exposed to 0.5–2 mg/mL ZnO NPs showed significantly lower ATP levels during the first 8.5 h compared to control, with stronger inhibition at 1–2 mg/mL than 0.5 mg/mL ZnO NPs (87 ± 1.4% inhibition versus 72 ± 2% inhibition; Figure 2B). During the first 8.5 h, the ATP levels in the presence of 1–2 mg/mL ZnO NPs stayed stable (Figure 2B), which indicates a bacteriostatic effect. By 30 h, ATP levels declined in bacteria treated with 1–2 mg/mL ZnO NPs (Figure 2B), which is consistent with the loss of viability observed by CFU counting (Figure 2A). These findings further demonstrate that ZnO NPs exert dose-dependent antibacterial activity on *S. mutans* UA159 with a MIC of 0.5 mg/mL.

### 3.3. ZnO NPs Inhibit Acid Production by S. mutans in a Concentration-Dependent Manner

**Figure 3:**
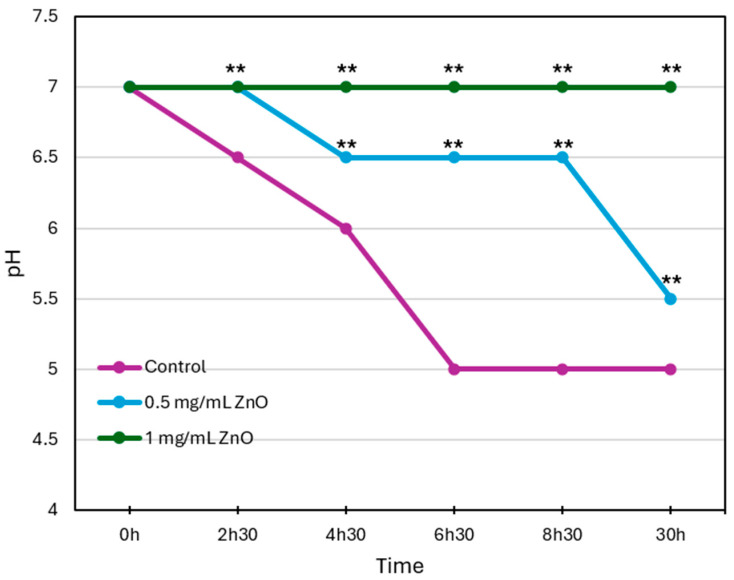
Effect of ZnO NPs on the pH of *S. mutans* cultures over a 30 h incubation period. The pH of the culture medium was monitored at different time points to evaluate acid production in the presence of various concentrations of ZnO NPs (0.5 and 1 mg/mL) compared with the untreated control. ** *p* < 0.005 compared to control bacteria.

The pH of the culture medium was monitored over a period of 30 h to assess the effect of different ZnO NPs concentrations on acid production by *S. mutans*. As shown in Figure 3, in the untreated control group (purple line), the pH dropped from 7.0 to 5.0 within 6.5 h, indicating significant acid production by the bacteria. In the presence of ZnO NPs, the decrease in pH values was much slower and less severe. At 0.5 mg/mL ZnO NPs (blue line), the pH dropped to 6.5 after 8.5 h and to 5.5 after 30 h. At 1 mg/mL ZnO NPs (green line), the pH remained stable at around 7.0 during the entire 30 h incubation period tested. These findings suggest that ZnO NPs reduce acid production by *S. mutans* in a concentration-dependent manner, likely due to a decrease in bacterial viability or metabolic activity.

### 3.4. SYTO 9/PI Live/Dead Staining Suggests That ZnO NPs Induce Cytoplasmic Leakage

**Figure 4:**
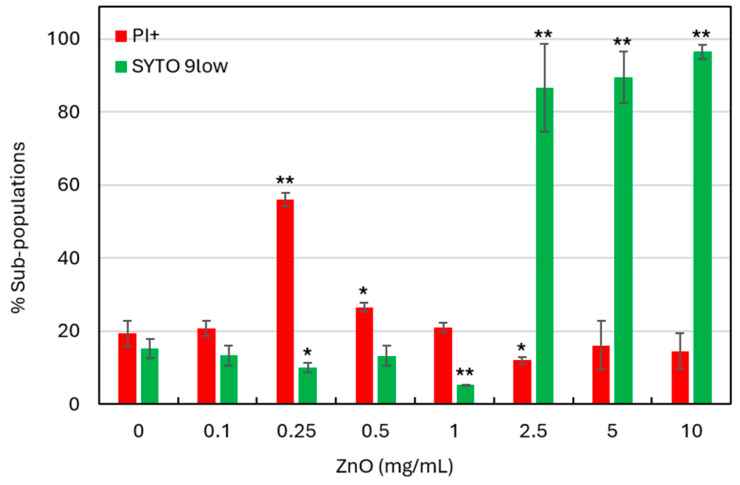
ZnO NPs induce membrane damage and cytoplasmic leakage in *S. mutans. S. mutans* cultures were treated with the indicated concentrations of ZnO NPs for 6 h, followed by SYTO 9/PI live/dead staining and analysis by flow cytometry. The percentage of PI^+^ and SYTO 9^low^ are presented. The graph presents the mean ±SD of three replicates. * *p* < 0.05, ** *p* < 0.005 compared to control bacteria.

Flow cytometry with SYTO 9 and propidium iodide (PI) live/dead staining was used to investigate the effect of ZnO NPs on *S. mutans* viability after a 6 h treatment. SYTO 9 is a nucleic acid stain that labels both live and dead cells; however, when nucleic acids have leaked out of the cell due to membrane destruction, the bacterium exhibits reduced green fluorescence intensity (here termed SYTO 9^low^). PI is a positively charged dye that penetrates only cells with compromised membranes, thereby marking dead or dying cells. However, if the cells have lost their nucleic acid due to severe membrane damage, these bacteria will not be stained properly with PI. Therefore, in this study, we have categorized the bacterial population into PI^+^ cells (dead or dying with permeable membranes), and SYTO 9^low^ (dead bacteria with cytoplasmic leakage) [46]. As shown in Figure 4, a 6 h treatment with 0.25 mg/mL ZnO NPs caused the appearance of a PI^+^ population (56 ± 1.8%), indicating that ZnO NPs comprises membrane integrity. Interestingly, with increasing ZnO NP concentrations (≥0.5 mg/mL), this population disappears, and at ≥2.5 mg/mL, the majority of the bacterial population (>90%) appeared as SYTO 9^low^ (Figure 4). This shift suggests a concentration-dependent effect of ZnO NPs, where higher concentrations induce extensive cytoplasmic leakage and loss of nucleic acid staining. Moreover, these findings show that ZnO NPs already at the ½ MIC of 0.25 mg/mL affect bacterial viability, with increasing cytotoxicity at MIC and higher concentrations.

### 3.5. ZnO NPs-Induced Morphological Changes in S. mutans as Demonstrated by HR-SEM Imaging

**Figure 5:**
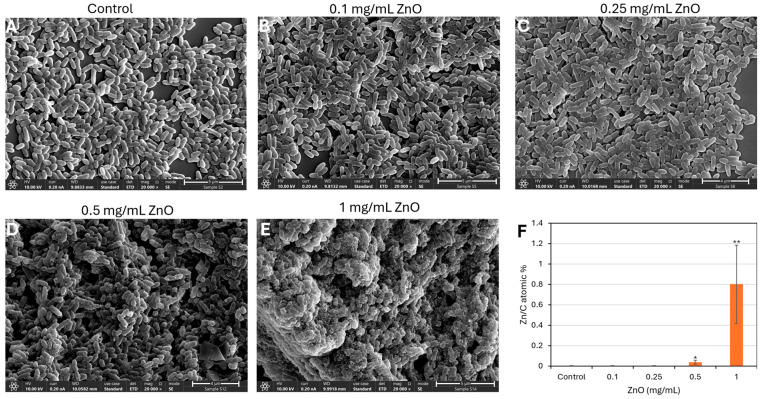
High-resolution scanning electron microscopy (HR-SEM) images of planktonic growing *S. mutans* following 2 h incubation with increasing concentrations of ZnO NPs. (**A**) Control (untreated); (**B**) 0.1 mg/mL ZnO NPs; (**C**) 0.25 mg/mL ZnO NPs; (**D**) 0.5 mg/mL ZnO NPs; (**E**) 1 mg/mL ZnO NPs. Images were acquired double-blinded at ×20,000 magnification. (**F**) Summary of EDS analysis of Zn/C content in 3 arbitrary sites of each sample as presented in Supplementary Data S3. * *p* < 0.05, ** *p* < 0.005.

To further investigate the impact of ZnO NPs on bacterial morphology, high-resolution scanning electron microscopy (HR-SEM) was performed on planktonic *S. mutans* cells following a 2 h incubation with increasing concentrations of ZnO NPs (Figure 5). Untreated control cells (Figure 5A) exhibited their characteristic ovoid morphology with well-defined membrane folded surface structures indicative of cell division [56], often arranged in short chains or clusters. Cells treated with 0.1 and 0.25 mg/mL ZnO NPs (Figure 5B,C) appeared with smoother surfaces with less folded surface structures. Exposure to 0.5 mg/mL ZnO NPs (Figure 5D) resulted in evident morphological cell damage, including collapsed or shrunken cells and the presence of nanoparticle aggregates adhering to the cell surface. At the highest concentration tested (1 mg/mL, Figure 5E), the majority of cells appeared severely distorted or lysed, with widespread membrane rupture and amorphous debris surrounding the remnants of bacterial structures.

EDS analysis showed a dose-dependent increase in the amount of Zn associated with the bacteria in ZnO-treated bacteria (Figure 5F and Supplementary Data S3).

Together, these observations reveal a clear concentration-dependent effect of ZnO NPs on *S. mutans* morphology, consistent with the membrane compromise and cytoplasmic leakage observed in the live/dead staining assay (Figure 4), supporting a direct interaction between ZnO NPs and the bacterial cell membrane.

### 3.6. ZnO NPs Induce ROS Production

**Figure 6:**
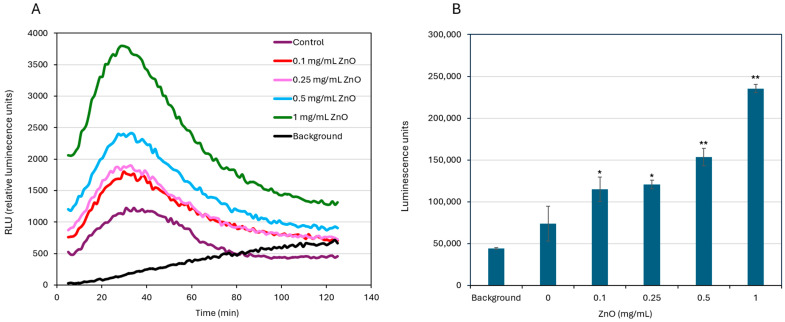
ZnO NPs increase ROS production *in S. mutans*. *S. mutans* was incubated in HBSS supplemented with 1% glucose, 50 µM Luminol and 4 U/mL HRP in the absence or presence of the indicated concentrations of ZnO NPs, and the luminescence was measured every minute for 125 min. Reactive oxygen species (ROS) are converted into radicals by horseradish peroxidase (HRP), which then oxidize luminol to emit luminescence. (**A**) Untreated bacteria (no ZnO NPs) showed moderate luminescence due to basic ROS production, while treatment with ZnO NPs induced a concentration-dependent increase in luminescence, with the strongest response observed at 1 mg/mL. Background (1 mg/mL ZnO) showed negligible luminescence, confirming that the luminescence originated from the bacteria. (**B**) Quantification of total ROS production during the 125 min test period expressed as the area under the curve (AUC ± SD) calculated from data presented in panel A. The AUC values demonstrate a dose-dependent increase in ROS levels in response to ZnO NP treatment. * *p* < 0.05, ** *p* <0.005 compared to control bacteria.

Reactive oxygen species (ROS) levels were measured in *S. mutans* cultures exposed to different concentrations of ZnO NPs over time. As shown in Figure 6, ROS production increased with higher ZnO NP concentrations. The highest ROS production was observed at 1 mg/mL, followed by 0.5 and 0.25 mg/mL ZnO NPs, while untreated control samples showed the lowest (basal) ROS levels (Figure 6).

### 3.7. ZnO NPs Treatment Causes a Slight Increase in Membrane Potential

**Figure 7:**
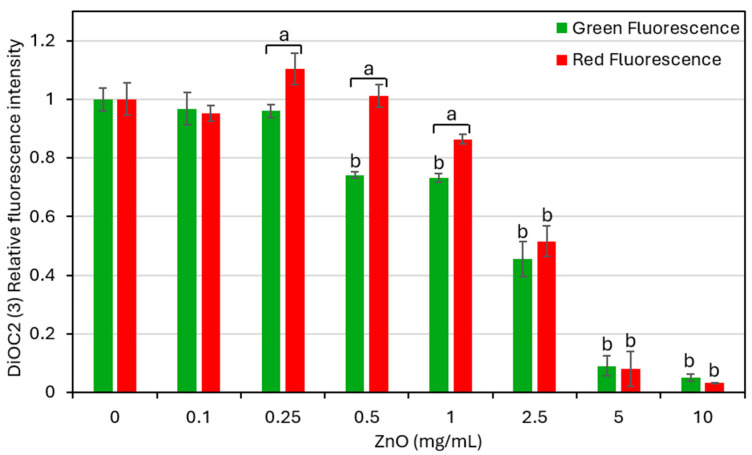
Effect of ZnO NPs on the membrane potential of *S. mutans.* Membrane potential was assessed using the potentiometric dye DiOC2(3). Green (530 nm) and red (610/620 nm) fluorescence intensities were measured by flow cytometry after a 30 min exposure to increasing ZnO NP concentrations (0–10 mg/mL). Data are presented as mean ± SD of triplicate experiments. a: *p* < 0.05 when comparing red fluorescence intensity with green fluorescent intensity. b: *p* < 0.05 when comparing the fluorescent intensity of treated cells with that of untreated control bacteria.

Changes in membrane potential were measured by flow cytometry using the fluorescent potentiometric dye DiOC2(3) after a 30 min treatment of *S. mutans* with different concentrations of ZnO NPs. A relatively higher intensity of red fluorescence in comparison to green fluorescence is an indication of membrane hyperpolarization. All fluorescence intensity calculations were performed relative to untreated control bacteria, which were set to 1 (Figure 7). Treatment with ZnO NPs at 0.5–10 mg/mL caused a concentration-dependent decrease in green fluorescence intensity, suggesting partial cytoplasmic leakage at 0.5–2.5 mg/mL and severe cytoplasmic leakage at 5 and 10 mg/mL (Figure 7). At 0.25–1 mg/mL, the red fluorescence intensity was relatively higher than the green fluorescence intensity (Figure 7), suggesting that ZnO NPs have induced membrane hyperpolarization. At higher concentrations (≥2.5 mg/mL), ZnO NPs resulted in a marked reduction in both fluorescent signals, which may be attributed to cytoplasmic leakage and bacterial cell death.

### 3.8. Treatment with ZnO NPs Leads to Reduced EPS Production

**Figure 8:**
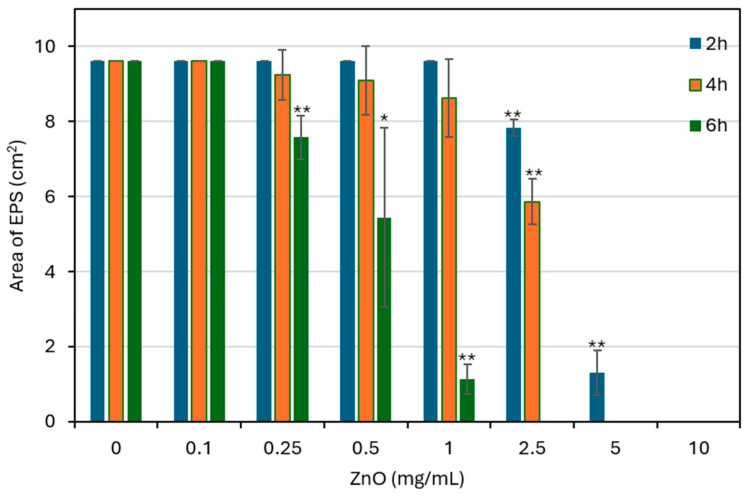
Effect of ZnO on EPS production by *S. mutans. S. mutans* was exposed to the indicated concentrations of ZnO NPs for 2, 4 and 6 h, and then 10 µL of the bacterial suspension was spotted on Conge Red agar plates for a 24 h incubation. The black area around the bacteria, indicative of EPS production, was measured by ImageJ software. EPS production was measured after 2, 4, and 6 h of exposure to increasing ZnO concentrations (0–10 mg/mL). Data are presented as mean ± SD of triplicate experiments. * *p* < 0.05, ** *p* < 0.005 compared to control bacteria.

Extracellular polysaccharide substance (EPS) production was measured using the Congo red staining technique, after 2, 4, and 6 h of bacterial exposure to different ZnO NPs concentrations. At low ZnO NP concentration (0.1 mg/mL), the EPS production was similar to control bacteria across all time points (Figure 8). Starting at 0.25 mg/mL, EPS production began to decrease, especially after 6 h (Figure 8). The reduction became more pronounced at 0.5–2.5 mg/mL, where a clear drop in EPS production was seen over time (Figure 8). At 5 mg/mL and above, EPS formation was nearly absent (Figure 8), which might be due to the antibacterial action of ZnO NPs. These results show that ZnO NPs significantly affect EPS production, which are essential for biofilm matrix formation.

### 3.9. Effect of ZnO NPs on Gene Expression

**Figure 9:**
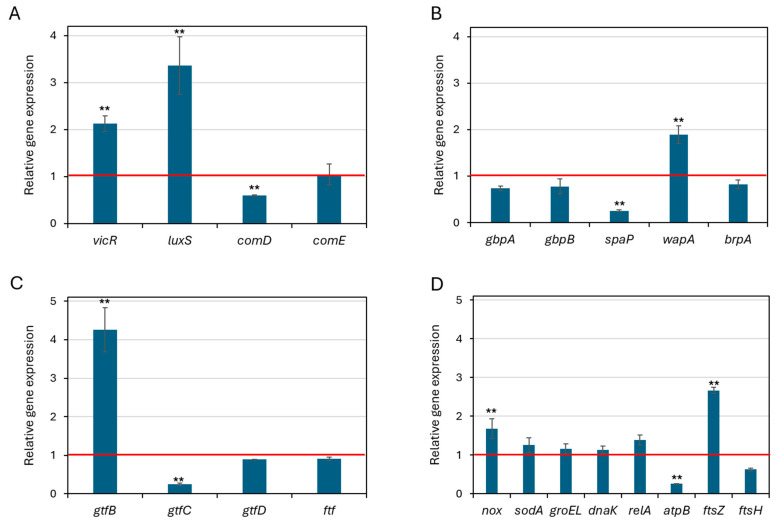
Effect of ZnO NPs on gene expression in *S. mutans***.** Relative expression levels of selected genes were measured by real-time qPCR after 2 h exposure to 0.25 mg/mL ZnO NPs compared with untreated control. Expression levels were normalized to the housekeeping gene *gyrA* and fold-changes calculated using the 2^−ΔΔCt^ method. (**A**) Quorum sensing–related genes. (**B**) Genes involved in regulating biofilm formation and membrane integrity. (**C**) EPS production-related genes. (**D**) Stress response and cell division-related genes. Several genes showed significant upregulation or downregulation in response to ZnO NPs, indicating that sub-inhibitory concentrations modulate stress adaptation and virulence pathways. Data are presented as mean ± SD of triplicate experiments. *n* = 3, ** *p* < 0.005 compared to control bacteria.

To obtain a mechanistic insight into the antibacterial and anti-biofilm activity of ZnO NPs, *S. mutans* was exposed to 0.25 mg/mL ZnO NPs for 2 h, and changes in expression of genes involved in quorum sensing, biofilm formation, stress responses and cell division were studied by real-time qPCR. ZnO NPs induced significant changes in the expression of certain genes. The quorum sensing genes *vicR* and *luxS* were significantly upregulated by 2.1 ± 0.2-fold and 3.4 ± 0.6-fold, respectively (*p* < 0.01), while *comD* and *comE* showed no significant changes (Figure 9A). These genes represent different quorum sensing systems in *S. mutans* [57]. Genes related to biofilm structure and cell envelope integrity were differentially affected by ZnO NPs: *wapA*, which facilitates cell–cell interactions within biofilms [58], was upregulated, whereas *spaP*, associated with initial adhesion and early colonization [59], was significantly downregulated (Figure 9B), suggesting that under ZnO-induced stress, the bacteria prioritize maintaining existing biofilm structure over initiating new attachment. Among the EPS-related genes, *gtfB* was strongly induced (4.3 ± 0.6), whereas *gtfC* was downregulated (4.2 ± 0.03), suggesting altered biofilm matrix composition (Figure 9C). The oxidative stress response gene *nox* was significantly upregulated, while *sodA*, *groEL*, and *dnaK* were only slightly upregulated (Figure 9D), suggesting an activated stress response. The cell division gene *ftsZ* was highly upregulated by 2.7 ± 0.1-fold (*p* < 0.05), while *atpB*, involved in energy metabolism, was significantly downregulated 3.8 ± 0.01-fold (*p* < 0.05), reflecting energetic stress. These findings indicate that even short-term (2 h) exposure to ZnO NPs initiates substantial transcriptional changes in *S. mutans*, affecting core processes such as stress adaptation, biofilm formation, and energy regulation.

### 3.10. Cytotoxic Effect of ZnO NPs

To evaluate the cytotoxicity of ZnO NPs, Vero epithelial cells and HEK-293T epithelial cells were exposed to concentrations ranging from 0 to 10 mg/mL for a 24 h. Cellular metabolic activity was assessed by the MTT assay. A significant reduction in cell viability was observed at 39 µg/mL and higher concentrations.

## 4. Discussion

Oral biofilms play a central role in diseases such as caries, gingivitis, periodontitis, and peri-implantitis [60,61]. Furthermore, dentures and implants can serve as surfaces for biofilms growth, potentially leading to infections [62,63,64]. *S. mutans* is considered an early colonizer of the tooth surface and play a central role in biofilm development [3]. It adheres to surfaces, produces extracellular polysaccharides (EPS), and tolerate acidic conditions, which allows it to persist within complex microbial communities [65]. Because of these features, *S. mutans* contribute not only to caries initiation but also to the ecological stability of pathogenic biofilms. Effort has been directed toward finding new strategies that target *S. mutans* and weaken its virulent traits.

Zinc oxide nanoparticles (ZnO NPs) have attracted attention for their broad-spectrum antibacterial and antibiofilm activity with good biocompatibility [24,66]. Their modes of action are diverse, involving disruption of bacterial membranes, induction of oxidative stress, and release of zinc ions [22,23,25,67]. While ZnO NPs has been studied against several pathogens, its specific effects on *S. mutans* physiology and virulence remain not fully understood. In this study, we aimed to investigate the impact of ZnO NPs on *S. mutans* and to explore its potential use in oral health applications.

Although the antimicrobial and antibiofilm activities of ZnO nanoparticles against *S. mutans* is documented [30,31,32,33,34,35], the mechanism of action on this bacterium is still not fully understood. Despite these contributions, a thorough explanation of how uncoated, pure ZnO NPs influence *S. mutans* cellular behavior has remained incomplete. Our findings help bridge this gap by showing that ZnO NPs trigger a combination of interrelated responses, including elevated ROS generation, loss of membrane potential, reduced intracellular ATP levels, impaired EPS production, and downregulation of key virulence and stress-associated genes. Together, these outcomes illustrate a coordinated mechanism through which ZnO nanoparticles disrupt *S. mutans* survival and biofilm development.

Our findings show that ZnO NPs inhibits *S. mutans* growth in a concentration-dependent manner. At 0.5 mg/mL, ZnO NPs significantly inhibited the growth and viability of *S. mutans*, as confirmed by turbidity, crystal violet, and MTT assays. The reduction in turbidity indicates decreased bacterial growth, while weaker crystal violet staining points to reduced biomass accumulation and adhesion, suggesting an inhibitory effect of ZnO NPs on biofilm formation. The MTT assay further confirmed a decrease in bacterial metabolic activity, supporting our hypothesis that ZnO NPs have bacteriostatic or bactericidal effects. The consistency across these complementary methods strengthens the evidence for ZnO NP’s antimicrobial potential. While turbidity and crystal violet cannot distinguish live from dead cells, and MTT may underestimate dormant but viable populations, the combined results clearly show that ZnO NPs disrupt both the growth and biofilm-associated virulence of *S. mutans*. At higher concentrations of ZnO NPs, the suppression was almost complete. These findings highlight the potential utility of zinc oxide in preventive strategies, making it a valuable approach to limiting virulence. This is consistent with the finding of Hamad and Atiyea [32] showing a MIC value of 0.312 mg/mL for several *S. mutans* clinical isolates from human dental caries and Hernández-Sierra et al. [68] who observed a MIC of 0.5 mg/mL against some *S. mutans* strains. Another study included bioinspired ZnO NPs showing a MIC of 75–100 μg/mL against two *S. mutans* strains [34]. Comparisons between such materials and our commercial ZnO NPs should therefore be interpreted cautiously, as synthesis method, surface charge, and particle coating strongly influence reactivity and biological effects.

One of the early responses to ZnO NPs exposure was a rapid change in membrane potential. Because membrane potential is critical for ATP synthesis, nutrient transport, and pH regulation, its disruption explains the reduced energy state of the bacteria. Similar hyperpolarization responses have been reported when *S. mutans* is treated with antibacterial compounds such as AEA [50], CBG [55] and CBD [69]. The proton-pumping F_1_F_0_-ATPase plays a central role in regulating this potential [70]. It is therefore likely that one of the effects of ZnO NPs on *S. mutans* might be the targeting of F_1_F_0_-ATPase. Clarifying the exact molecular targets will require further study.

ROS measurements showed a dose-dependent increase in oxidative stress upon ZnO NPs exposure, with higher ROS levels at 1 mg/mL and above. The ROS surge likely contributes to membrane lipid peroxidation, enzyme inactivation, and protein damage, providing a mechanistic basis for the observed changes in membrane potential and dye uptake [23].

Energy metabolism was also strongly affected. ATP levels remained low in ZnO NPs-treated cultures across multiple time points, particularly at higher concentrations. ATP is essential for growth, biofilm matrix formation, and acid production. This drop fits well with the other results we observed. The loss of energy is consistent with the membrane damage and oxidative stress described earlier, helping explain the reduced viability and virulence.

ZnO NP treatment also suppressed EPS production. Because EPS is crucial for biofilm structure and bacterial adhesion [3,71], its inhibition points to a direct anti-biofilm effect of ZnO NPs in addition to the general antibacterial activity. This dual action - limiting bacterial survival and disrupting the biofilm matrix - makes ZnO NPs especially appealing for dental applications.

Although ZnO NPs concentrations above MIC show strong inhibition, killing is not immediate. CFU assays revealed that even at 5–10 mg/mL ZnO NPs, viable bacteria persisted for several hours in a bacteriostatic state, with cell death only after 24 h. The late death of the bacteria enabled us to study the early effects of ZnO NPs on these cells.

HR-SEM imaging shows significant changes in the bacterial morphology already at sub-MIC of 0.1–0.25 mg/mL with more smooth surfaces with fewer septum-initiating sites. At the MIC of 0.5 mg/mL, several protrusions and dots are observed at the bacterial surface which seems to be adhesion of ZnO NPs to the bacteria together with distortions of the bacterial cell membrane. This was even more pronounced at the 2 × MIC, where most of the bacteria appeared with distorted structures. The affinity of ZnO NPs to bacteria membrane and to the bacteria was verified by EDS analysis, which demonstrated strong association between the ZnO NPs and the bacteria.

At the transcriptional level, a short 2 h exposure to sub-MIC ZnO NPs concentration (0.25 mg/mL) induced broad changes in gene expression in *S. mutans*. Notably, the stress response gene *nox* was significantly upregulated. This might be an adaptive response to the ZnO-induced ROS production. *nox* encodes for NADH oxidase, a flavin-containing oxidoreductase, which reduces diatomic oxygen (O_2_) to H_2_O through the oxidation of NADH to NAD^+^, thereby preventing the formation of damaging ROS [72,73]. Its expression is regulated by oxygen through *SpxA* and by *Rex* [73]. The regeneration of NAD^+^ is essential for the production of pyruvate in the carbon cycle [73], which is an important energy source for *S. mutans* [74]. In contrast to *nox*, there were no significant alterations in the expression of *sod1* encoding superoxide dismutase and the two chaperones *groEL* and *dnaK*, which are upregulated in response to acid stress [75].

The quorum-sensing regulators *vicR* and *luxS* were also significantly upregulated, suggesting that ZnO NPs-induced stress stimulates communication networks that may influence biofilm dynamics. The *luxS* gene plays an important role in the ability of *S. mutans* to adapt to environmental stress. Mutants lacking this system exhibit reduced tolerance to acid and oxidative stress and form weaker, irregular biofilms compared to the wild-type strain [76,77]. Moreover, transcriptomic and proteomic studies have shown that disruption of *luxS* alters the expression of many genes involved in metabolism, transport, and stress responses, highlighting its important role in helping the cell adapt to environmental changes [78]. Together, these findings underscore the dual function of *luxS* in metabolic homeostasis and AI-2–mediated signaling, both of which are essential for virulence and biofilm development in *S. mutans*.

*VicR* positively regulates *gtfB* and *gtfC* [9], supporting the observed transcriptional coupling between quorum-sensing and biofilm-related genes. Genes related to biofilm formation showed divergent regulation: while *gtfB* (EPS synthesis) and *wapA* (cell–cell interaction) were upregulated, *gtfC* and *spaP* (initial adhesion and early colonization) were downregulated, indicating a remodeling of EPS composition and surface interactions under ZnO exposure. Altogether, these findings suggest that ZnO NPs do not simply suppress biofilm-related genes but drive *S. mutans* into a stress-adaptive state characterized by oxidative stress responses, altered quorum sensing, and selective modulation of virulence pathways. Although some biofilm-associated genes were upregulated, these shifts seem not to be sufficient to overcome the energy loss and structural damage caused by ZnO NPs, which ultimately reduce bacterial biofilm formation.

Another important finding is the significant downregulation of *atpB*, encoding a subunit of the F_1_F_0_-ATPase. F_1_F_0_-ATPase is important for acid tolerance by hydrolyzing ATP to pump protons out of the cell [79]. A reduction in F_1_F_0_-ATPase activity may increase the bacterial sensitivity to acid stress and affect the membrane potential important for ATP production [80], which is in line with reduced ATP levels in bacteria treated with ZnO NPs.

The upregulation of the cell-division gene *ftsZ* is interesting in light of its central role in bacterial cytokinesis by forming the Z-ring at the future site of septum formation. Changes in its expression can interfere with normal bacterial division [81,82]. The observed increase in *ftsZ* expression may either contribute directly to growth inhibition by leading to aberrant or premature septum formation or represent a compensatory response to ZnO-induced stress. *ftsZ* has been shown to be upregulated in *S. mutans* by other antibacterial agents [50] and its GTPase activity is elevated by acidic stress [83]. Altogether, the alterations in gene expression highlight the profound impact of ZnO on bacterial regulatory networks.

A further consideration is the potential cytotoxicity of ZnO NPs in the dental field and toward human oral cells. Reported effects vary widely across studies: human gingival and periodontal ligament fibroblasts show reduced viability at concentrations of 40–100 µg/mL [84,85], whereas other mammalian cells, such as WI-38 normal lung epithelial, HepG2 hepatocellular carcinoma and HEK-293T epithelial cells, tolerate much higher levels (400–500 µg/mL) [31,86]. Comparisons between such materials should therefore be interpreted cautiously, as synthesis method, surface charge, size, and particle drug delivery platform strongly influence reactivity and biological effects.

Similarly, ZnO NPs incorporated into a poly(lactic-co-glycolic acid) (PLGA) matrix were found to reduce cell viability of human gingival fibroblasts in a dose- and time-dependent manner, with significant toxicity at concentrations ≥40 µg/mL after 48 h [84]. Our findings show that the ZnO NPs of Sigma are cytotoxic to both Vero epithelial cells and HEK-293T epithelial cells at a concentration of 39 µg/mL. Nevertheless, ZnO incorporated into toothpastes and varnishes has demonstrated low cytotoxicity [87]. These differences highlight that toxicity depends strongly on cell type, nanoparticle properties, and exposure conditions. Although the antibacterial concentration used in our study (0.5 mg/mL) exceeds some reported cytotoxic thresholds, such levels represent free ZnO NPs suspensions rather than immobilized ZnO in pharmaceutical formulations used in different applications. Future studies should evaluate ZnO-based drug delivery technologies that maintain antimicrobial efficacy while ensuring oral cell compatibility.

The main limitation of this study is the use of only a single bacterial species (*S. mutans UA159*) to investigate the mode of action and monospecies biofilm formation under stationary conditions. Although the current findings provide valuable mechanistic insights into the antibacterial effects of ZnO NPs, it is important to recognize that biofilm-associated bacteria often display distinct physiological and resistance characteristics compared to their planktonic counterparts. Therefore, future studies should aim to expand these investigations to multispecies biofilm models, in order to better understand how ZnO NPs influence biofilm architecture and long-term bacterial behavior under conditions that more closely resemble the oral environment.

## 5. Conclusions

In summary, this study demonstrates that ZnO nanoparticles have both bacteriostatic and bactericidal effects against the cariogenic bacterium *S. mutans*, with a minimum inhibitory concentration (MIC) of 0.5 mg/mL. An antibacterial effect was already observed at the sub-MIC of 0.25 mg/mL, as shown by increased membrane permeability, although surviving bacteria resumed growth. The minimum biofilm inhibitory concentration (MBIC) was similar to the MIC, suggesting that the antibiofilm effect is largely due to antibacterial activity. Our findings, however, suggest that ZnO NPs also directly inhibit biofilm formation by reducing EPS production and affecting the expression of biofilm-related genes. Our findings further show that the antibacterial effect is caused by a combination of multiple mechanisms, including the induction of ROS production, alterations in membrane properties resulting in cytoplasmic leakage and bacterial death. Additionally, ZnO NPs prevent acidification caused by *S. mutans*, which is one of the major virulence factors, together with biofilm formation in dental caries formation. At higher ZnO NP concentrations, there was even an increase in the pH to pH 8, which is advantageous for maintaining an above neutral pH in its microenvironment. Our study supports the potential of ZnO NPs in dental applications to control bacterial growth and biofilm formation.

## Figures and Tables

**Table 1:** Primers used for real-time qPCR of the indicated *S. mutans* genes [50,55].

Genes	Forward Primers	Reverse Primers	Biological Function
*gyrA*	TACAGGTGATGTCATGGGTAAATA C	CCG GGT AGT ACT TCC ATT AGG TCA C	DNA gyrase subunit; housekeeping gene.
*gtfB*	AGCAATGCAGCCAATCTACAAAT	ACGAACTTTGCCGTTATTGTCA	Glycosyltransferase-I; makes water-insoluble glucans (EPS).
*gtfC*	GGTTTAACGTCAAAATTAGCTGTATT	CTCAACCAACCGCCACTGTT	Glycosyltransferase-SI; makes both water soluble and water-insoluble glucans (EPS).
*gtfD*	CAGGCAGCCAACGCATTAA	AGCCCTCGCTCATCATAAGC	Glucosyltransferase-D, makes water-soluble glucans.
*ftf*	AAATATGAAGGCGGCTACAACG	CTTCACCAGTCTTAGCATCCTGAA	Fructosyltransferase; makes fructans (EPS).
*gbpA*	GGTGGTTCTGTGCCTGATGA	TTGCCAGCCTGATACACGTT	Glucan-binding protein, promotes adhesion and biofilm stability.
*gbpB*	AGGGCAATGTACTTGGGGTG	TTTGGCCACCTTGAACACCT	Glucan-binding protein B; binds glucans, regulates biofilm formation.
*spaP*	GACTTTGGTAATGGTTATGCATCAA	TTTGTATCAGCCGGATCAAGTG	Surface antigen I/II; adhesion (sucrose-independent biofilm).
*wapA*	GCACGCTTGCAGTACATTGC	CATAAGGTCGCGAGCAGCT	Wall protein; adhesion and biofilm
*brpA*	GGAGGAGCTGCATCAGGATTC	AACTCCAGCACATCCAGCAAG	Biofilm-regulating protein A; controls biofilm formation, acid and oxidative stress, and cell division.
*nox*	GGGTTGTGGAATGGCACTTTGG	CAATGGCTGTCACTGGCGATTC	NADH oxidase; ROS scavenger.
*sodA*	GCAGTGCTAAGACTCCCGAATC	TTGCGGAAGTGTGAGATTGGC	Superoxide dismutase; oxidative stress defense.
*groEL*	CCAGGAGCTTTGACTGCGAC	TTGCGGATGATGATGTAGATGGT	Chaperone (Hsp60); stress response.
*dnaK*	GCAGGTCAAGAGGGAGCTCA	CCGCCCTTGTCTGAGAATC	Chaperone (Hsp70); stress response.
*relA*	ACAAAAAGGGTATCGTCCGTACAT	AATCACGCTTGGTATTGCTAATTG	GTP diphosphokinase; stringent response, biofilm regulation.
*atpB*	AGCCAACCTTGGCAACTGAAA	TGTCAGACGGCGTTCAAGGTT	β-subunit of F_1_F_0_-ATPase, ATP synthesis and acid tolerance.
*ftsZ*	CAACCAAGAGCACAACAGCAAG	ACGACGAAGATTCCAATCGCC	Cell division protein; Z-ring formation.
*ftsH*	TGTTCCGTTCTTCTCTATTTCTGG	GCACGCTCTGCTTTCTTAGC	ATP-dependent protease, stress response and cell division.
*vicR*	CGCAGTGGCTGAGGAAAATG	ACCTGTGTGTGTCGCTAAGTGATG	Response regulator of cell wall and biofilm genes.
*luxS*	ACTGTTCCCCTTTTGGCTGTC	AACTTGCTTTGATGACTGTGGC	Autoinducer-2 synthase; quorum sensing, biofilm.
*comD*	TGAAAATAGCATAGGTGAGTCAAAG	ATTTAGGTTAGCTGATTAACACTATACAC	Histidine kinase, part of ComDE quorum sensing system.
*comE*	CACAACAACTTATTGACGCTATCCC	TGATTGGCTACTTCCAGTCCTTTC	Response regulator of ComDE, controls competence/biofilm genes.

## Data Availability

All relevant data are presented in the manuscript. Raw data for the figures are available upon reasonable request from the corresponding author.

## Supplementary Materials

The following supporting information can be downloaded at: https://www.mdpi.com/article/10.3390/biom15121660/s1, Supplementary Data S1: Certificate of analysis of ZnO from Sigma-Aldrich; Supplementary Data S2: Characterization of ZnO nanoparticles; Supplementary Data S3: EDS analysis of zinc content on bacterial samples.

## Abbreviations

The following abbreviations are used in this manuscript:

CFU	Colony forming units
CV	Crystal violet
EDS	Energy Dispersive X-ray Spectroscopy
EPS	Extracellular polysaccharides substance
HR-SEM	High-resolution scanning electron microscopy
MBIC	Minimum biofilm inhibitory concentration
MIC	Minimum inhibitory concentration
ROS	Reactive oxygen species
*S. mutans*	*Streptococcus mutans*
ZnO NPs	Zinc oxide nanoparticles

## References

[1] Valm A.M. (2019). The Structure of Dental Plaque Microbial Communities in the Transition from Health to Dental Caries and Periodontal Disease. J. Mol. Biol..

[2] Wang C., Hou J., van der Mei H.C., Busscher H.J., Ren Y. (2019). Emergent Properties in *Streptococcus mutans* Biofilms Are Controlled through Adhesion Force Sensing by Initial Colonizers. mBio.

[3] Lemos J.A., Palmer S.R., Zeng L., Wen Z.T., Kajfasz J.K., Freires I.A., Abranches J., Brady L.J. (2019). The Biology of *Streptococcus mutans*. Microbiol. Spectr..

[4] Koo H., Xiao J., Klein M.I., Jeon J.G. (2010). Exopolysaccharides produced by *Streptococcus mutans* glucosyltransferases modulate the establishment of microcolonies within multispecies biofilms. J. Bacteriol..

[5] Yoshida E., Imai S., Hanada N., Hayakawa T. (2013). Biofilm Bormation on Titanium and Hydroxyapatite Surface using Artificial Mouth System. J. Hard Tissue Biol..

[6] Laosuwan K., Epasinghe D.J., Wu Z., Leung W.K., Green D.W., Jung H.S. (2018). Comparison of biofilm formation and migration of *Streptococcus mutans* on tooth roots and titanium miniscrews. Clin. Exp. Dent. Res..

[7] Dani S., Prabhu A., Chaitra K.R., Desai N.C., Patil S.R., Rajeev R. (2016). Assessment of *Streptococcus mutans* in healthy versus gingivitis and chronic periodontitis: A clinico-microbiological study. Contemp. Clin. Dent..

[8] Fitri D.K., Tuygunov N., Wan Harun W.H.A., Purwasena I.A., Cahyanto A., Zakaria M.N. (2025). Key virulence genes associated with *Streptococcus mutans* biofilm formation: A systematic review. Front. Oral Health.

[9] Zheng T., Jing M., Gong T., Yan J., Wang X., Xu M., Zhou X., Zeng J., Li Y. (2023). Regulatory mechanisms of exopolysaccharide synthesis and biofilm formation in *Streptococcus mutans*. J. Oral Microbiol..

[10] Nilsson M., Jakobsen T.H., Givskov M., Twetman S., Tolker-Nielsen T. (2019). Oxidative stress response plays a role in antibiotic tolerance of *Streptococcus mutans* biofilms. Microbiology.

[11] Høiby N., Bjarnsholt T., Givskov M., Molin S., Ciofu O. (2010). Antibiotic resistance of bacterial biofilms. Int. J. Antimicrob. Agents.

[12] Bowen W.H., Koo H. (2011). Biology of *Streptococcus mutans*-derived glucosyltransferases: Role in extracellular matrix formation of cariogenic biofilms. Caries Res..

[13] Krzyściak W., Jurczak A., Kościelniak D., Bystrowska B., Skalniak A. (2014). The virulence of *Streptococcus mutans* and the ability to form biofilms. Eur. J. Clin. Microbiol. Infect. Dis..

[14] Sims K.R., Maceren J.P., Liu Y., Rocha G.R., Koo H., Benoit D.S.W. (2020). Dual antibacterial drug-loaded nanoparticles synergistically improve treatment of *Streptococcus mutans* biofilms. Acta Biomater..

[15] DeCesaris D.S., Hayashi M.A.L., Vickerman M.M., Rickard A.H., Tenuta L.M.A. (2025). Dextranase enhances nanoparticle penetration of *S. mutans* biofilms. J. Oral Microbiol..

[16] Sahli C., Moya S.E., Lomas J.S., Gravier-Pelletier C., Briandet R., Hémadi M. (2022). Recent advances in nanotechnology for eradicating bacterial biofilm. Theranostics.

[17] Gudkov S.V., Burmistrov D.E., Serov D.A., Rebezov M.B., Semenova A.A., Lisitsyn A.B. (2021). A mini review of antibacterial properties of ZnO nanoparticles. Front. Phys..

[18] Lallo da Silva B., Caetano B.L., Chiari-Andréo B.G., Pietro R., Chiavacci L.A. (2019). Increased antibacterial activity of ZnO nanoparticles: Influence of size and surface modification. Colloids Surf. B Biointerfaces.

[19] Vitasovic T., Caniglia G., Eghtesadi N., Ceccato M., Bo Jesen E.D., Gosewinkel U., Neusser G., Rupp U., Walther P., Kranz C. (2024). Antibacterial Action of Zn^2+^ Ions Driven by the in vivo Formed ZnO Nanoparticles. ACS Appl. Mater. Interfaces.

[20] Lahiri D., Ray R.R., Sarkar T., Upadhye V.J., Ghosh S., Pandit S., Pati S., Edinur H.A., Abdul Kari Z., Nag M. (2022). Anti-biofilm efficacy of green-synthesized ZnO nanoparticles on oral biofilm: In vitro and in silico study. Front. Microbiol..

[21] Riahi S., Moussa N.B., Lajnef M., Jebari N., Dabek A., Chtourou R., Guisbiers G., Vimont S., Herth E. (2023). Bactericidal activity of ZnO nanoparticles against multidrug-resistant bacteria. J. Mol. Liq..

[22] Zhang L., Jiang Y., Ding Y., Povey M., York D. (2007). Investigation into the antibacterial behaviour of suspensions of ZnO nanoparticles (ZnO nanofluids). J. Nanopart. Res..

[23] Tiwari V., Mishra N., Gadani K., Solanki P.S., Shah N.A., Tiwari M. (2018). Mechanism of Anti-bacterial Activity of Zinc Oxide Nanoparticle Against Carbapenem-Resistant *Acinetobacter baumannii*. Front. Microbiol..

[24] Sirelkhatim A., Mahmud S., Seeni A., Kaus N.H.M., Ann L.C., Bakhori S.K.M., Hasan H., Mohamad D. (2015). Review on zinc oxide nanoparticles: Antibacterial activity and toxicity mechanism. Nano-Micro Lett..

[25] Dwivedi S., Wahab R., Khan F., Mishra Y.K., Musarrat J., Al-Khedhairy A.A. (2014). Reactive oxygen species mediated bacterial biofilm inhibition via zinc oxide nanoparticles and their statistical determination. PLoS ONE.

[26] Lee J.H., Kim Y.G., Cho M.H., Lee J. (2014). ZnO nanoparticles inhibit *Pseudomonas aeruginosa* biofilm formation and virulence factor production. Microbiol. Res..

[27] McQuillan J.S., Shaw A.M. (2014). Whole-cell *Escherichia coli*-based bio-sensor assay for dual zinc oxide nanoparticle toxicity mechanisms. Biosens. Bioelectron..

[28] Pelgrift R.Y., Friedman A.J. (2013). Nanotechnology as a therapeutic tool to combat microbial resistance. Adv. Drug Deliv. Rev..

[29] Zhang R., Carlsson F., Edman M., Hummelgård M., Jonsson B.G., Bylund D., Olin H. (2018). *Escherichia coli* Bacteria Develop Adaptive Resistance to Antibacterial ZnO Nanoparticles. Adv. Biosyst..

[30] Andrade V., Martínez A., Rojas N., Bello-Toledo H., Flores P., Sánchez-Sanhueza G., Catalán A. (2018). Antibacterial activity against *Streptococcus mutans* and diametrical tensile strength of an interim cement modified with zinc oxide nanoparticles and terpenes: An in vitro study. J. Prosthet. Dent..

[31] Barma M.D., Muthupandiyan I., Samuel S.R., Amaechi B.T. (2021). Inhibition of *Streptococcus mutans*, antioxidant property and cytotoxicity of novel nano-zinc oxide varnish. Arch. Oral Biol..

[32] Hamad A.M., Atiyea Q.M. (2021). In vitro study of the effect of zinc oxide nanoparticles on *Streptococcus mutans* isolated from human dental caries. J. Phys. Conf. Ser..

[33] Mirhosseini F., Amiri M., Daneshkazemi A., Zandi H., Javadi Z.S. (2019). Antimicrobial Effect of Different Sizes of Nano Zinc Oxide on Oral Microorganisms. Front. Dent..

[34] Soni J., Revathi D., Dhanraj G., Ramasubburayan R. (2024). Bioinspired green synthesis of ZnO nanoparticles by marine-derived Streptomyces plicatus and its multifaceted biomedicinal properties. Microb. Pathog..

[35] Tiwari A.K., Jha S., Singh A.K., Mishra S.K., Pathak A.K., Ojha R.P., Yadav R.S., Dikshit A. (2022). Innovative Investigation of Zinc Oxide Nanoparticles Used in Dentistry. Crystals.

[36] Shaik M.R., Kandaswamy K., Guru A., Khan H., Giri J., Mallik S., Shah M.A., Arockiaraj J. (2024). Piperine-coated zinc oxide nanoparticles target biofilms and induce oral cancer apoptosis via BCl-2/BAX/P53 pathway. BMC Oral Health.

[37] Jiang N., Zhao S., Wang S., Lu Z. (2021). Proteomics of *Streptococcus mutans* to Reveal the Antibiofilm Formation Mechanism of Ag/ZnO Nanocomposites with Light-Emitting Diode Radiation. Int. J. Nanomed..

[38] Jiang J., Pi J., Cai J. (2018). The Advancing of Zinc Oxide Nanoparticles for Biomedical Applications. Bioinorg. Chem. Appl..

[39] Xiao D., Huang Y., Fang Z., Liu D., Wang Q., Xu Y., Li P., Li J. (2025). Zinc oxide nanoparticles for skin wound healing: A systematic review from the perspective of disease types. Mater. Today Bio.

[40] Govindarajan D.K., Mohanarangam M., Kadirvelu L., Sivaramalingam S.S., Jothivel D., Ravichandran A., Periasamy S., Kandaswamy K. (2025). Biofilms and oral health: Nanotechnology for biofilm control. Discov. Nano.

[41] Wang Y., Hua H., Li W., Wang R., Jiang X., Zhu M. (2019). Strong antibacterial dental resin composites containing cellulose nanocrystal/zinc oxide nanohybrids. J. Dent..

[42] Alves M.J., Grenho L., Lopes C., Borges J., Vaz F., Vaz I.P., Fernandes M.H. (2018). Antibacterial effect and biocompatibility of a novel nanostructured ZnO-coated gutta-percha cone for improved endodontic treatment. Mater. Sci. Eng. C Mater. Biol. Appl..

[43] Eshed M., Lellouche J., Matalon S., Gedanken A., Banin E. (2012). Sonochemical coatings of ZnO and CuO nanoparticles inhibit *Streptococcus mutans* biofilm formation on teeth model. Langmuir.

[44] Mohd Bakhori S.K., Mahmud S., Ling C.A., Sirelkhatim A.H., Hasan H., Mohamad D., Masudi S.M., Seeni A., Abd Rahman R. (2017). In-vitro efficacy of different morphology zinc oxide nanopowders on *Streptococcus sobrinus* and *Streptococcus mutans*. Mater. Sci. Eng. C Mater. Biol. Appl..

[45] Zada M.H., Kubek M., Khan W., Kumar A., Domb A. (2019). Dispersible hydrolytically sensitive nanoparticles for nasal delivery of thyrotropin releasing hormone (TRH). J. Control. Release.

[46] Chamlagain M., Hu J., Sionov R.V., Steinberg D. (2024). Anti-bacterial and anti-biofilm activities of arachidonic acid against the cariogenic bacterium *Streptococcus mutans*. Front. Microbiol..

[47] Sionov R.V., Siag A., Mersini E.T., Kogan N.M., Alkhazov T., Koman I., Rowlo P., Gutkin V., Gross M., Steinberg D. (2025). The Incorporation of CBD into Biodegradable DL-Lactide/Glycolide Copolymers Creates a Persistent Antibacterial Environment: An In Vitro Study on *Streptococcus mutans* and *Staphylococcus aureus*. Pharmaceutics.

[48] Melkam A., Sionov R.V., Shalish M., Steinberg D. (2024). Enhanced Anti-Bacterial Activity of Arachidonic Acid against the Cariogenic Bacterium *Streptococcus mutans* in Combination with Triclosan and Fluoride. Antibiotics.

[49] Sionov R.V., Tsavdaridou D., Aqawi M., Zaks B., Steinberg D., Shalish M. (2021). Tooth mousse containing casein phosphopeptide-amorphous calcium phosphate prevents biofilm formation of *Streptococcus mutans*. BMC Oral Health.

[50] Wolfson G., Sionov R.V., Smoum R., Korem M., Polacheck I., Steinberg D. (2023). Anti-Bacterial and Anti-Biofilm Activities of Anandamide against the Cariogenic *Streptococcus mutans*. Int. J. Mol. Sci..

[51] Sionov R.V., Korem M., Polacheck I., Steinberg D. (2025). Cannabidiol (CBD) Acts as an Antioxidant on *Gardnerella vaginalis*, Resulting in Reduced Metabolic Activity, Loss of Survivability, and Elimination of Biofilms. Antibiotics.

[52] Aqawi M., Sionov R.V., Gallily R., Friedman M., Steinberg D. (2021). Anti-Biofilm Activity of Cannabigerol against *Streptococcus mutans*. Microorganisms.

[53] Sionov R.V., Assi S., Gershkovitz M., Sagiv J.Y., Polyansky L., Mishalian I., Fridlender Z.G., Granot Z. (2015). Isolation and Characterization of Neutrophils with Anti-Tumor Properties. J. Vis. Exp..

[54] Bernabè G., Pauletto A., Zamuner A., Cassari L., Castagliuolo I., Brun P., Dettin M. (2022). Exploiting Conserved Quorum Sensing Signals in *Streptococcus mutans* and *Streptococcus pneumoniae*. Microorganisms.

[55] Aqawi M., Sionov R.V., Friedman M., Steinberg D. (2023). The Antibacterial Effect of Cannabigerol toward *Streptococcus mutans* Is Influenced by the Autoinducers 21-CSP and AI-2. Biomedicines.

[56] Zamakhaeva S., Chaton C.T., Rush J.S., Ajay Castro S., Kenner C.W., Yarawsky A.E., Herr A.B., van Sorge N.M., Dorfmueller H.C., Frolenkov G.I. (2021). Modification of cell wall polysaccharide guides cell division in *Streptococcus mutans*. Nat. Chem. Biol..

[57] Wright P.P., Ramachandra S.S. (2022). Quorum Sensing and Quorum Quenching with a Focus on Cariogenic and Periodontopathic Oral Biofilms. Microorganisms.

[58] Zhu L., Kreth J., Cross S.E., Gimzewski J.K., Shi W., Qi F. (2006). Functional characterization of cell-wall-associated protein WapA in *Streptococcus mutans*. Microbiology.

[59] Yang J., Deng D., Brandt B.W., Nazmi K., Wu Y., Crielaard W., Ligtenberg A.J.M. (2019). Diversity of SpaP in genetic and salivary agglutinin mediated adherence among *Streptococcus mutans* strains. Sci. Rep..

[60] Lasserre J.F., Brecx M.C., Toma S. (2018). Oral Microbes, Biofilms and Their Role in Periodontal and Peri-Implant Diseases. Materials.

[61] Colombo A.P.V., Tanner A.C.R. (2019). The Role of Bacterial Biofilms in Dental Caries and Periodontal and Peri-implant Diseases: A Historical Perspective. J. Dent. Res..

[62] Busscher H.J., Rinastiti M., Siswomihardjo W., van der Mei H.C. (2010). Biofilm formation on dental restorative and implant materials. J. Dent. Res..

[63] Arweiler N.B., Netuschil L. (2016). The Oral Microbiota. Adv. Exp. Med. Biol..

[64] Berger D., Rakhamimova A., Pollack A., Loewy Z. (2018). Oral Biofilms: Development, Control, and Analysis. High Throughput.

[65] Peterson S.N., Snesrud E., Liu J., Ong A.C., Kilian M., Schork N.J., Bretz W. (2013). The dental plaque microbiome in health and disease. PLoS ONE.

[66] Colon G., Ward B.C., Webster T.J. (2006). Increased osteoblast and decreased *Staphylococcus epidermidis* functions on nanophase ZnO and TiO2. J. Biomed. Mater. Res. A.

[67] Li M., Zhu L., Lin D. (2011). Toxicity of ZnO nanoparticles to *Escherichia coli*: Mechanism and the influence of medium components. Environ. Sci. Technol..

[68] Hernández-Sierra J.F., Ruiz F., Pena D.C., Martínez-Gutiérrez F., Martínez A.E., Guillén Ade J., Tapia-Pérez H., Castañón G.M. (2008). The antimicrobial sensitivity of *Streptococcus mutans* to nanoparticles of silver, zinc oxide, and gold. Nanomedicine.

[69] Barak T., Sharon E., Steinberg D., Feldman M., Sionov R.V., Shalish M. (2022). Anti-Bacterial Effect of Cannabidiol against the Cariogenic *Streptococcus mutans* Bacterium: An In Vitro Study. Int. J. Mol. Sci..

[70] Suzuki T., Tagami J., Hanada N. (2000). Role of F1F0-ATPase in the growth of *Streptococcus mutans* GS5. J. Appl. Microbiol..

[71] Karygianni L., Ren Z., Koo H., Thurnheer T. (2020). Biofilm Matrixome: Extracellular Components in Structured Microbial Communities. Trends Microbiol..

[72] Cheng X., Xu X., Zhou X., Ning J. (2024). Oxidative stress response: A critical factor affecting the ecological competitiveness of *Streptococcus mutans*. J. Oral Microbiol..

[73] Baker J.L., Derr A.M., Karuppaiah K., MacGilvray M.E., Kajfasz J.K., Faustoferri R.C., Rivera-Ramos I., Bitoun J.P., Lemos J.A., Wen Z.T. (2014). *Streptococcus mutans* NADH oxidase lies at the intersection of overlapping regulons controlled by oxygen and NAD^+^ levels. J. Bacteriol..

[74] Ahn S.J., Deep K., Turner M.E., Ishkov I., Waters A., Hagen S.J., Rice K.C. (2019). Characterization of LrgAB as a stationary phase-specific pyruvate uptake system in *Streptococcus mutans*. BMC Microbiol..

[75] Matsui R., Cvitkovitch D. (2010). Acid tolerance mechanisms utilized by *Streptococcus mutans*. Future Microbiol..

[76] Wen Z.T., Burne R.A. (2004). LuxS-mediated signaling in *Streptococcus mutans* is involved in regulation of acid and oxidative stress tolerance and biofilm formation. J. Bacteriol..

[77] Yoshida A., Ansai T., Takehara T., Kuramitsu H.K. (2005). LuxS-based signaling affects Streptococcus mutans biofilm formation. Appl. Environ. Microbiol..

[78] Sztajer H., Lemme A., Vilchez R., Schulz S., Geffers R., Yip C.Y., Levesque C.M., Cvitkovitch D.G., Wagner-Döbler I. (2008). Autoinducer-2-regulated genes in *Streptococcus mutans* UA159 and global metabolic effect of the luxS mutation. J. Bacteriol..

[79] Sekiya M., Izumisawa S., Iwamoto-Kihara A., Fan Y., Shimoyama Y., Sasaki M., Nakanishi-Matsui M. (2019). Proton-pumping F-ATPase plays an important role in *Streptococcus mutans* under acidic conditions. Arch. Biochem. Biophys..

[80] Benarroch J.M., Asally M. (2020). The Microbiologist’s Guide to Membrane Potential Dynamics. Trends Microbiol..

[81] Bottomley A.L., Liew A.T.F., Kusuma K.D., Peterson E., Seidel L., Foster S.J., Harry E.J. (2017). Coordination of Chromosome Segregation and Cell Division in *Staphylococcus aureus*. Front. Microbiol..

[82] Du S., Lutkenhaus J. (2019). At the Heart of Bacterial Cytokinesis: The Z Ring. Trends Microbiol..

[83] Chen Y., Li Y., Yuan C., Liu S., Xin F., Deng X., Wang X. (2022). *Streptococcus mutans* cell division protein FtsZ has higher GTPase and polymerization activities in acidic environment. Mol. Oral Microbiol..

[84] Mozaffari A., Mirzapour S.M., Rad M.S., Ranjbaran M. (2023). Cytotoxicity of PLGA-zinc oxide nanocomposite on human gingival fibroblasts. J. Adv. Periodontol. Implant. Dent..

[85] Seker S., Elçin A.E., Yumak T., Sınağ A., Elçin Y.M. (2014). In vitro cytotoxicity of hydrothermally synthesized ZnO nanoparticles on human periodontal ligament fibroblast and mouse dermal fibroblast cells. Toxicol. In Vitr..

[86] Selim Y.A., Azb M.A., Ragab I., HM Abd El-Azim M. (2020). Green Synthesis of Zinc Oxide Nanoparticles Using Aqueous Extract of Deverra tortuosa and their Cytotoxic Activities. Sci. Rep..

[87] Pushpalatha C., Suresh J., Gayathri V.S., Sowmya S.V., Augustine D., Alamoudi A., Zidane B., Mohammad Albar N.H., Patil S. (2022). Zinc Oxide Nanoparticles: A Review on Its Applications in Dentistry. Front. Bioeng. Biotechnol..

