# Supplementary material for: Mechanism of Action of Zinc Oxide Nanoparticles as an Antibacterial Agent Against Streptococcus mutans
Source: Biomolecules. 2025 Nov 27;15(12):1660. doi: 10.3390/biom15121660 (PMC12730517; doi:10.3390/biom15121660)

biomolecules

MDPI

Sigma-Aldrich.

3050 Spruce Street, Saint Louis, MO 63103, USA
Website: www.sigmaaldrich.com
Email USA: techserv@sial.com
Outside USA: eurtechserv@sial.com

Certificate of Analysis

Product Name:
Zinc oxide, dispersion - nanoparticles, <100 nm particle size (TEM), ≤40 nm avg. part. size (APS), 20 wt. % in H_2O

**Product Number:**	**721077**	ZnO
**Batch Number:**	**MKCT9826**	
Brand:	ALDRICH	
CAS Number:	1314-13-2	
Quality Release Date:	21 AUG 2023	

**Test**	**Specification**	**Result**
Appearance (Color)	Off White to Light Yellow and Light Beige to Beige	Off White
Appearance (Form)	Dispersion	Dispersion
ICP Major Analysis<br>Confirms Zinc Component	Confirmed	Confirmed
Size	< 100 nm	40 nm
pH	6.0 - 9.0	8.9
Concentration	18 - 22 % wt	21 % wt

Larry Coers, Director
Quality Control
Milwaukee, WI US

Supplementary data S1. Certificate of analysis of ZnO NPs from Sigma-Aldrich.

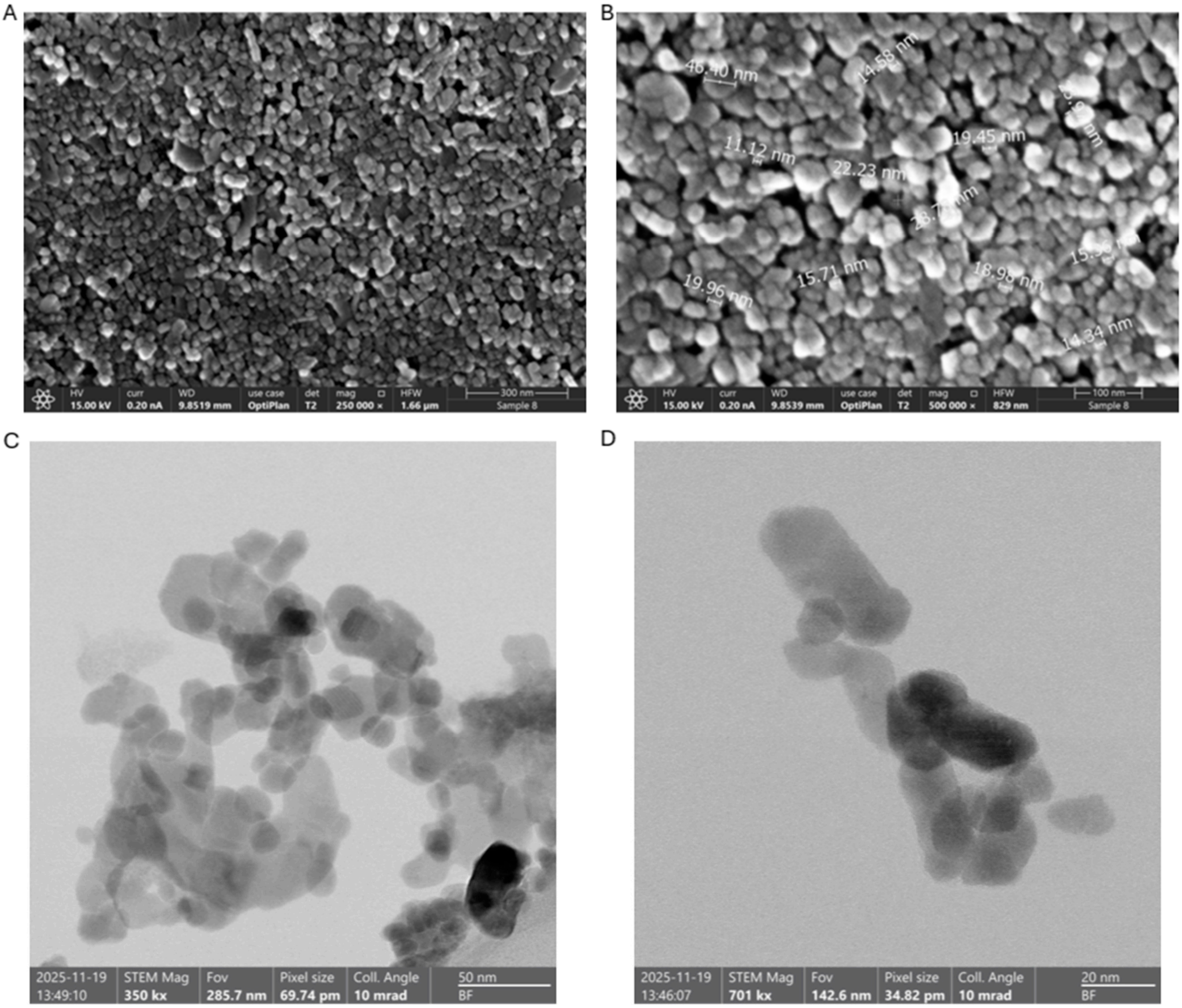

E

	Z-Average (nm)	Polydispersity Index (PI)
0.25 mg/mL ZnO in BHI	130.677	0.236
0.5 mg/mL ZnO in BHI	147.112	0.203
1 mg/mL ZnO in BHI	147.357	0.175
40 mg/mL ZnO in ddw	140.245	0.209

Supplementary data S2. Characterization of ZnO nanoparticles. Figure S2. Representative images of High-resolution scanning electron microscopy (HR-SEM) (**A.** and **B)** and Transmission Electron Microscopy (**C**. and **D**.) Size range between 10-47 nm. **E.** Table of Z values (130-147 nm) and Polydispersity Index (PI) obtained with Dynamic Light Scattering (DLS).

Supplementary data S3: EDS analysis of ZnO content on bacterial samples

EDS analysis of Zn content in 3 arbitrary sites of each sample of *S. mutans,* three samples of each concentration (0-1 mg/mL).

Sample 1 – Control

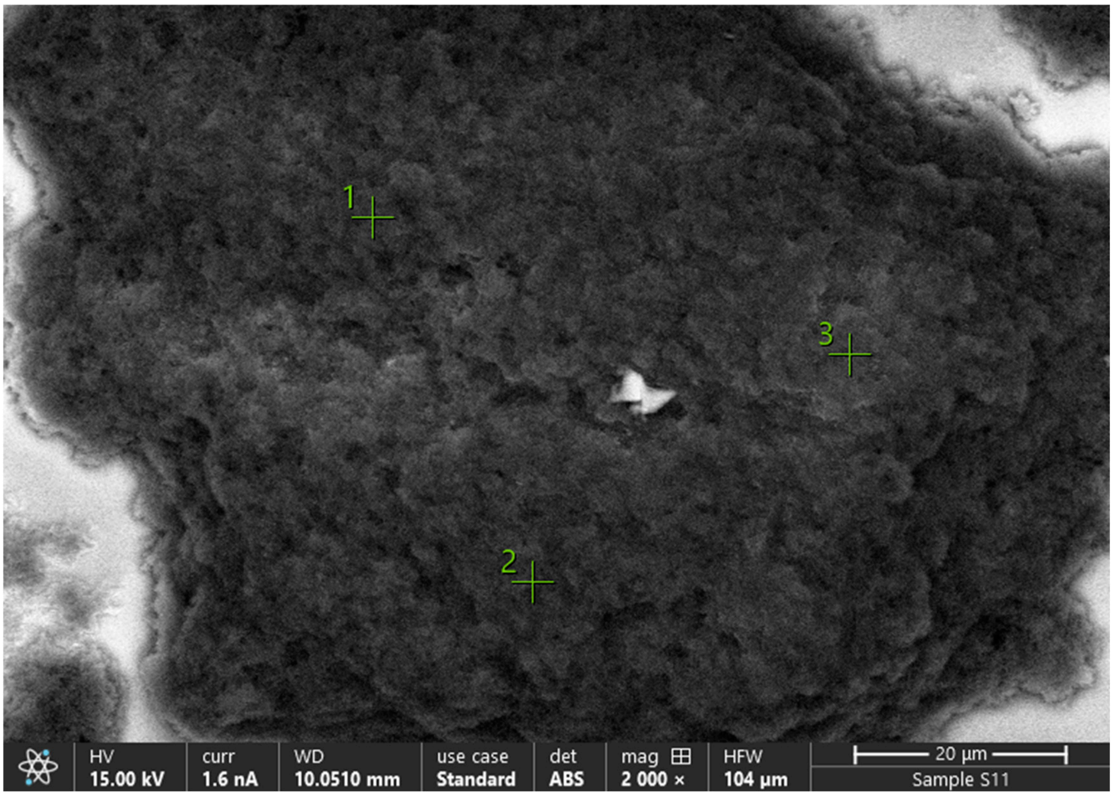

Point 1

Wt. % Error	At. % Error	Net Counts	Wt. %	At. %	Line	Element
0.2	0.3	79 728	36.0	49.6	K	C
0.2	0.2	49 631	31.2	32.1	K	O
0.0	0.0	15 779	2.6	1.9	K	Na
0.0	0.0	6 343	0.9	0.6	K	Mg
0.0	0.0	1 674	0.2	0.1	K	Al
0.1	0.0	160 968	18.7	11.0	K	Si
0.1	0.0	27 702	3.6	1.9	K	P
0.0	0.0	2 868	0.5	0.2	K	Cl
0.1	0.0	29 382	6.3	2.6	K	Ca
---	---	0	0.0	0.0	K	Zn

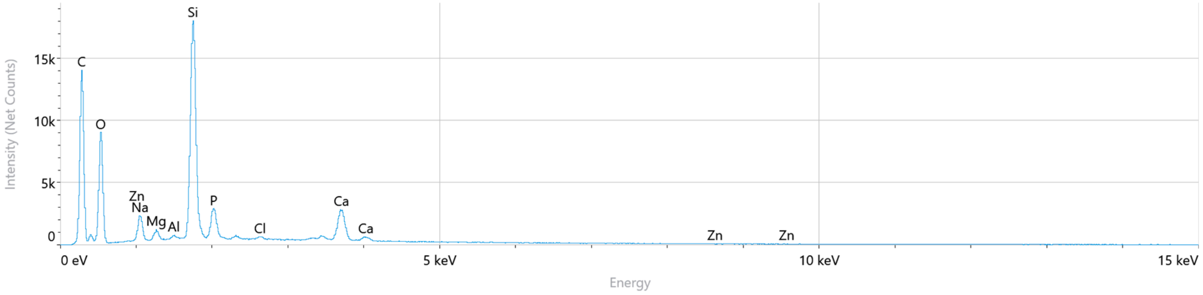

Point 2

Wt. % Error	At. % Error	Net Counts	Wt. %	At. %	Line	Element
0.2	0.3	68 893	35.8	48.5	K	C
0.2	0.3	46 506	34.7	35.4	K	O
0.1	0.1	14 085	3.0	2.1	K	Na
0.0	0.0	5 406	0.9	0.6	K	Mg
0.1	0.0	96 884	14.0	8.1	K	Si
0.1	0.0	31 893	5.0	2.6	K	P
0.0	0.0	3 526	0.7	0.3	K	Cl
0.1	0.0	22 489	5.9	2.4	K	Ca
---	---	0	0.0	0.0	K	Zn

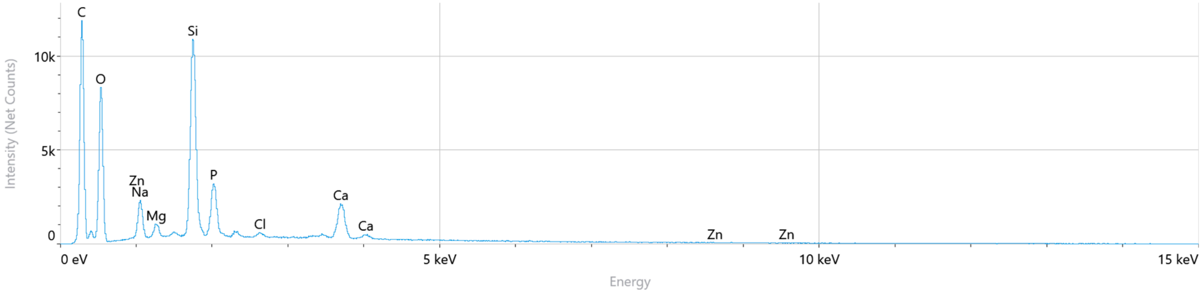

Point 3

Wt. % Error	At. % Error	Net Counts	Wt. %	At. %	Line	Element
0.2	0.3	72 557	34.6	47.7	K	C
0.2	0.2	50 702	33.2	34.4	K	O
0.0	0.0	15 450	2.8	2.1	K	Na
0.0	0.0	5 982	0.9	0.6	K	Mg
0.0	0.0	1 452	0.2	0.1	K	Al

Wt. % Error	At. % Error	Net Counts	Wt. %	At. %	Line	Element
0.1	0.0	133 214	16.8	9.9	K	Si
0.1	0.0	28 264	4.0	2.1	K	P
0.0	0.0	3 296	0.6	0.3	K	Cl
0.1	0.0	27 545	6.3	2.6	K	Ca
0.2	0.1	298	0.6	0.2	K	Zn

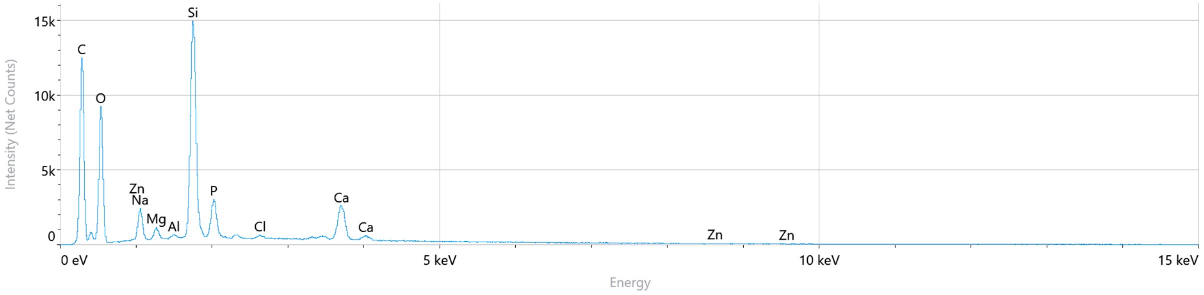

Sample 2 – Control

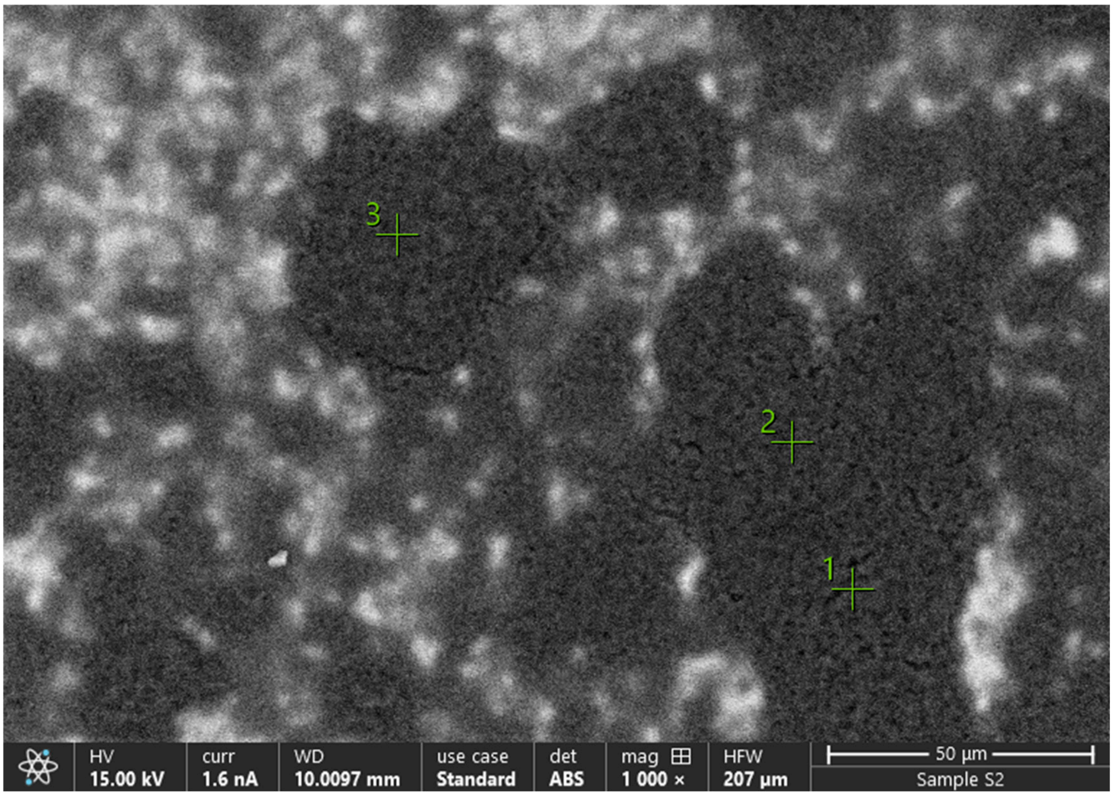

Point 1

Wt. % Error	At. % Error	Net Counts	Wt. %	At. %	Line	Element
0.2	0.2	96 846	33.0	48.1	K	C
0.2	0.2	60 537	22.5	24.6	K	O
0.0	0.0	39 166	3.4	2.6	K	Na
0.0	0.0	22 375	1.6	1.2	K	Mg
0.0	0.0	6 319	0.4	0.3	K	Al
0.1	0.1	479 074	31.2	19.5	K	Si
0.0	0.0	23 057	1.9	1.1	K	P
0.0	0.0	48 706	6.0	2.6	K	Ca
---	---	0	0.0	0.0	K	Zn

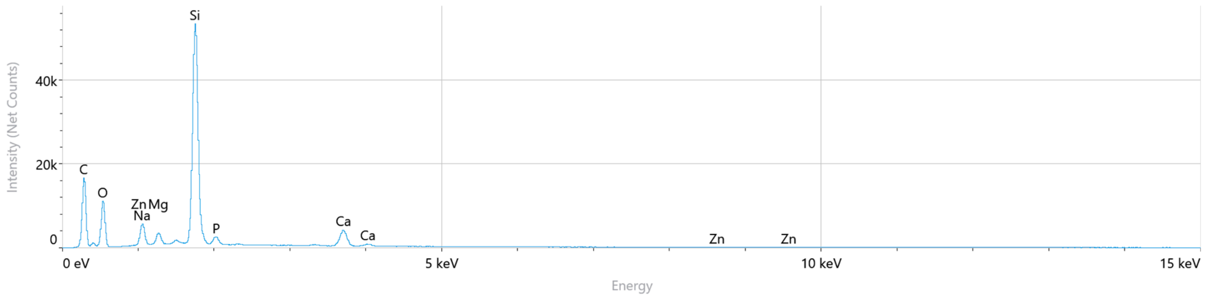

Point 2

Wt. % Error	At. % Error	Net Counts	Wt. %	At. %	Line	Element
0.2	0.2	90 510	32.1	46.2	K	C
0.5	0.6	2 942	3.2	3.9	K	N
0.2	0.2	54 851	24.3	26.1	K	O
0.0	0.0	22 520	2.4	1.8	K	Na
0.0	0.0	14 041	1.2	0.8	K	Mg
0.0	0.0	5 453	0.4	0.3	K	Al
0.1	0.1	370 614	27.8	17.0	K	Si
0.0	0.0	21 700	2.0	1.1	K	P
0.0	0.0	46 571	6.6	2.8	K	Ca
---	---	0	0.0	0.0	K	Zn

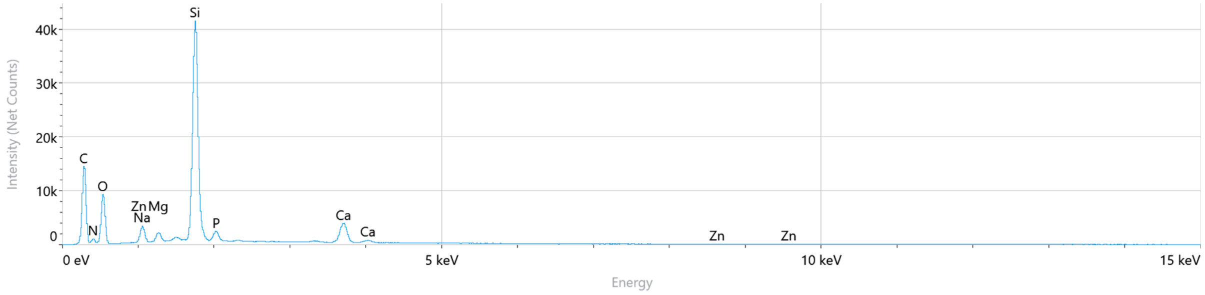

Point 3

Wt. % Error	At. % Error	Net Counts	Wt. %	At. %	Line	Element
0.2	0.3	78 531	32.8	47.5	K	C
0.2	0.2	52 435	25.5	27.8	K	O
0.0	0.0	22 243	2.7	2.0	K	Na
0.0	0.0	12 229	1.2	0.8	K	Mg

Wt. % Error	At. % Error	Net Counts	Wt. %	At. %	Line	Element
0.0	0.0	5 064	0.4	0.3	K	Al
0.1	0.1	322 004	27.6	17.2	K	Si
0.0	0.0	24 234	2.5	1.4	K	P
0.0	0.0	41 198	6.6	2.9	K	Ca
---	---	0	0.0	0.0	K	Zn
0.0	0.0	4 105	0.7	0.1	L	Mo

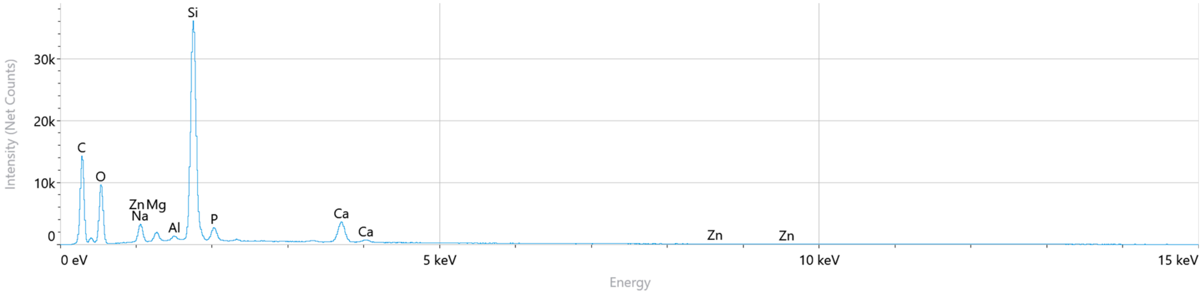

Sample 3 – Control

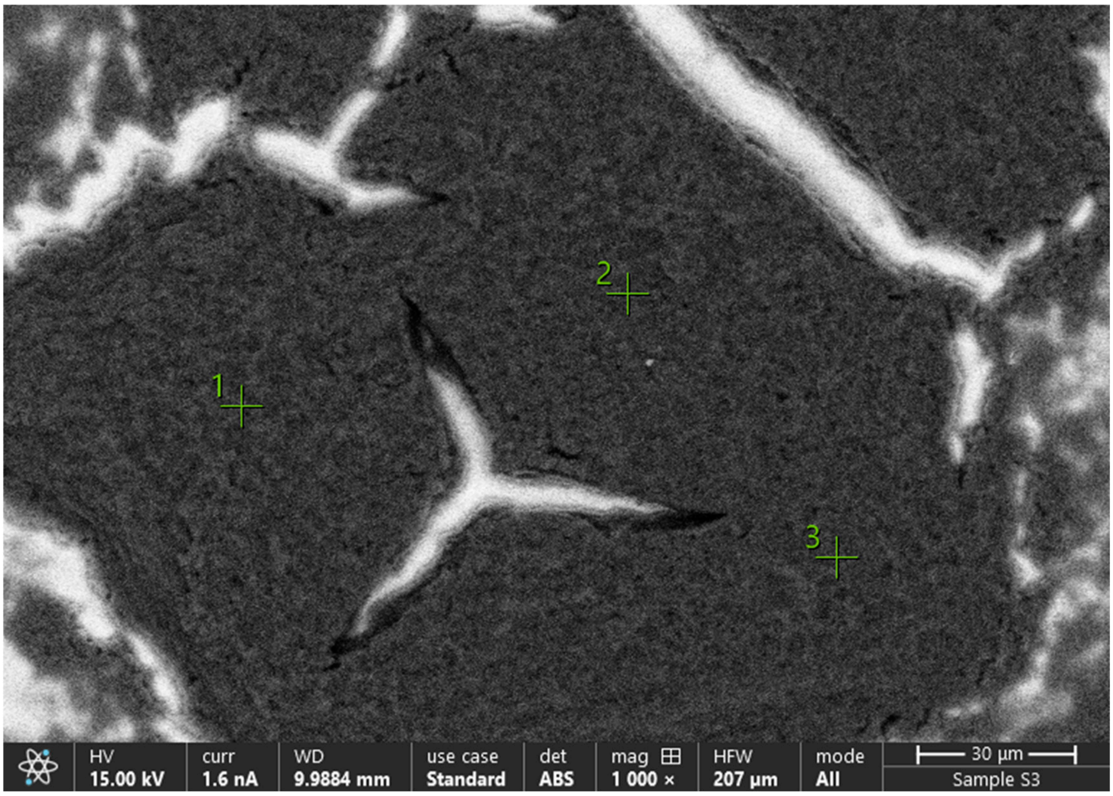

Point 1

Wt. % Error	At. % Error	Net Counts	Wt. %	At. %	Line	Element
0.2	0.2	97 734	34.5	45.0	K	C
0.7	0.8	4 793	6.6	7.4	K	N
0.3	0.3	59 358	36.3	35.5	K	O
0.0	0.0	13 269	2.3	1.6	K	Na
0.0	0.0	5 174	0.7	0.5	K	Mg
0.0	0.0	99 903	11.5	6.4	K	Si
0.1	0.0	30 414	3.7	1.9	K	P
0.0	0.0	3 329	0.4	0.2	K	S
0.1	0.0	18 807	3.9	1.5	K	Ca
0.1	0.0	73	0.1	0.0	K	Zn

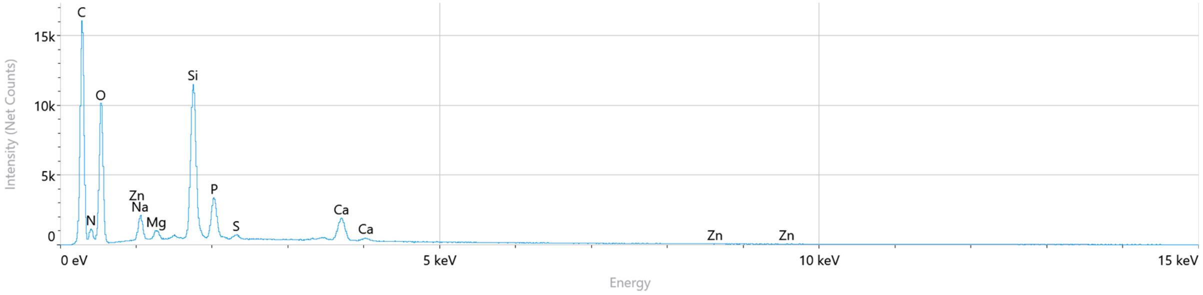

Point 2

Wt. % Error	At. % Error	Net Counts	Wt. %	At. %	Line	Element
0.2	0.2	86 046	33.1	43.8	K	C
0.4	0.5	4 670	6.9	7.8	K	N
0.3	0.3	53 924	35.8	35.5	K	O
0.0	0.0	12 415	2.3	1.6	K	Na
0.0	0.0	4 884	0.7	0.5	K	Mg
0.1	0.0	97 037	12.0	6.8	K	Si
0.1	0.0	27 273	3.6	1.8	K	P
0.0	0.0	2 890	0.4	0.2	K	S
0.1	0.0	21 967	4.9	1.9	K	Ca
0.2	0.1	129	0.3	0.1	K	Zn

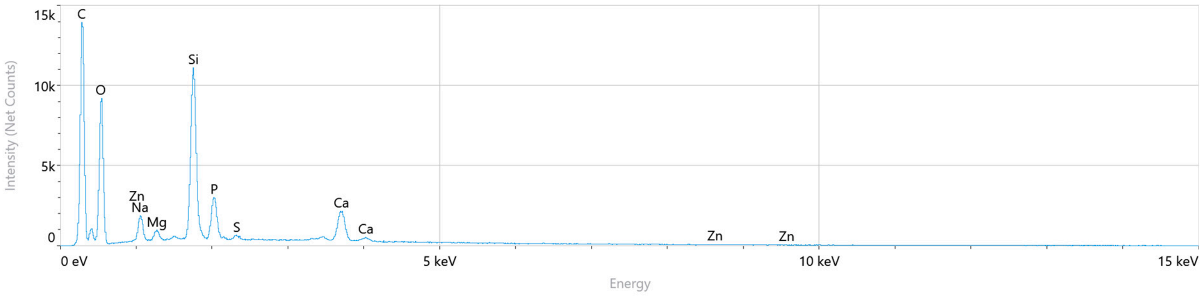

Point 3

Wt. % Error	At. % Error	Net Counts	Wt. %	At. %	Line	Element
0.2	0.3	92 517	37.1	50.2	K	C
0.2	0.2	56 390	33.0	33.4	K	O
0.0	0.0	13 380	2.1	1.5	K	Na
0.0	0.0	6 647	0.8	0.6	K	Mg

Wt. % Error	At. % Error	Net Counts	Wt. %	At. %	Line	Element
0.1	0.0	154 334	16.9	9.7	K	Si
0.1	0.0	34 425	4.2	2.2	K	P
0.1	0.0	29 349	5.9	2.4	K	Ca
---	---	0	0.0	0.0	K	Zn

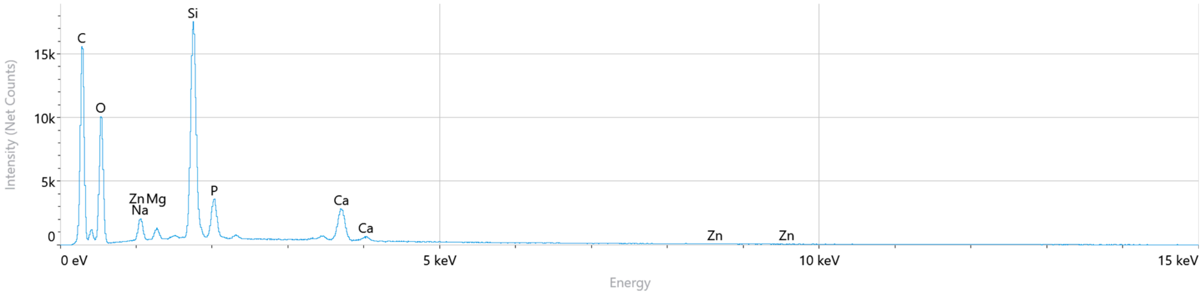

Sample 4 – 0.1 mg/mL ZnO

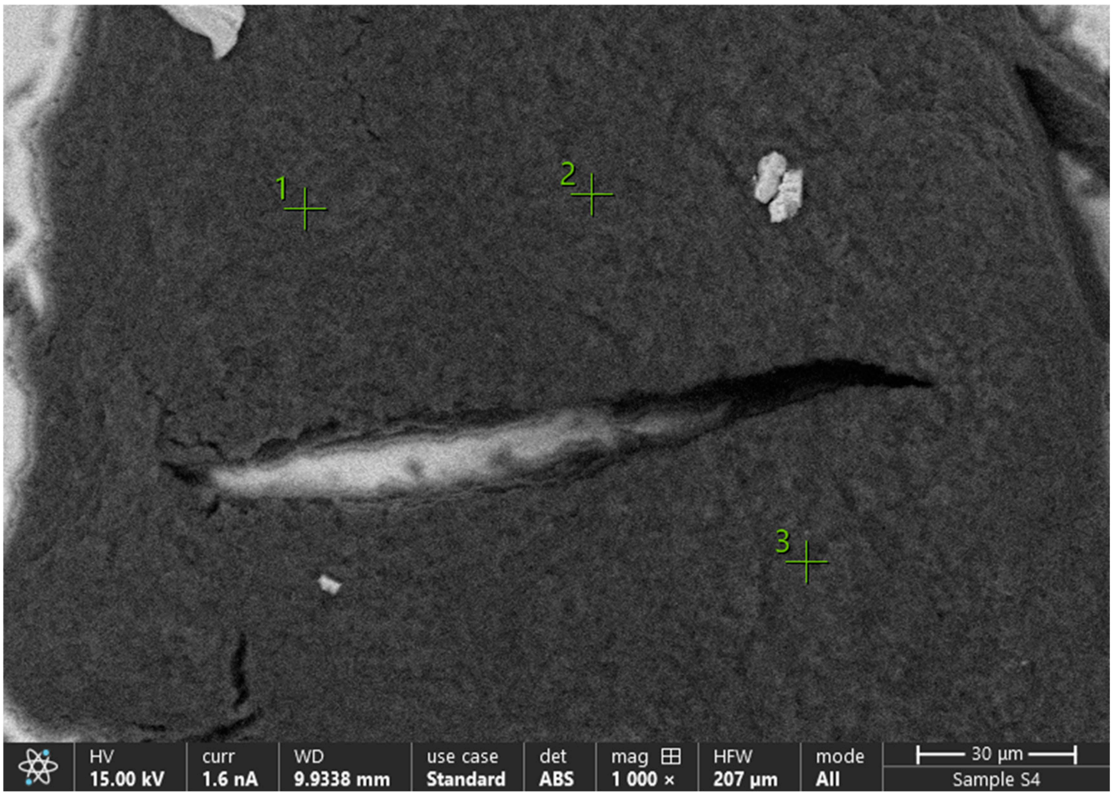

Point 1

Wt. % Error	At. % Error	Net Counts	Wt. %	At. %	Line	Element
0.2	0.2	82 399	31.7	41.7	K	C
0.6	0.7	4 278	5.5	6.2	K	N
0.3	0.3	69 721	39.5	39.0	K	O
0.0	0.0	18 719	3.3	2.3	K	Na
0.0	0.0	7 492	1.1	0.7	K	Mg
0.0	0.0	1 486	0.2	0.1	K	Al
0.0	0.0	96 543	11.5	6.5	K	Si
0.1	0.0	33 568	4.2	2.2	K	P
0.0	0.0	4 169	0.5	0.3	K	S
0.1	0.0	10 856	2.3	0.9	K	Ca
0.1	0.1	122	0.2	0.1	K	Zn

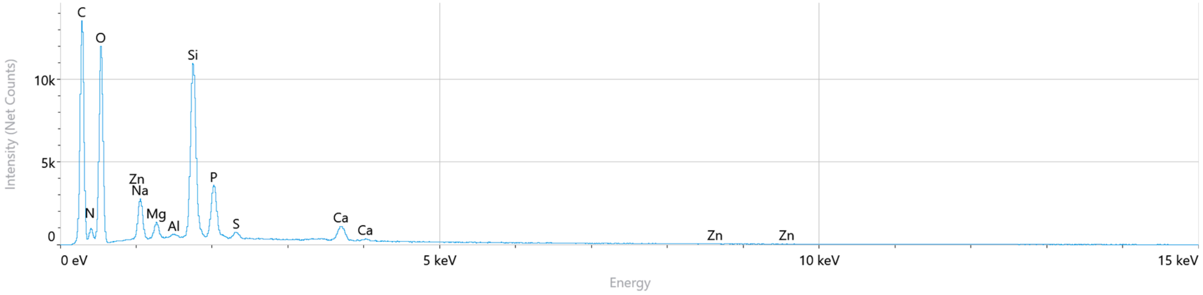

Point 2

Wt. % Error	At. % Error	Net Counts	Wt. %	At. %	Line	Element
0.2	0.2	131 551	36.6	46.7	K	C
0.6	0.7	3 484	3.9	4.2	K	N
0.3	0.3	87 924	40.4	38.7	K	O
0.0	0.0	18 206	2.6	1.8	K	Na
0.0	0.0	7 752	0.9	0.6	K	Mg
0.0	0.0	92 708	9.0	4.9	K	Si
0.1	0.0	48 838	4.9	2.4	K	P
0.0	0.0	10 055	1.7	0.7	K	Ca
0.0	0.0	14	0.0	0.0	K	Zn

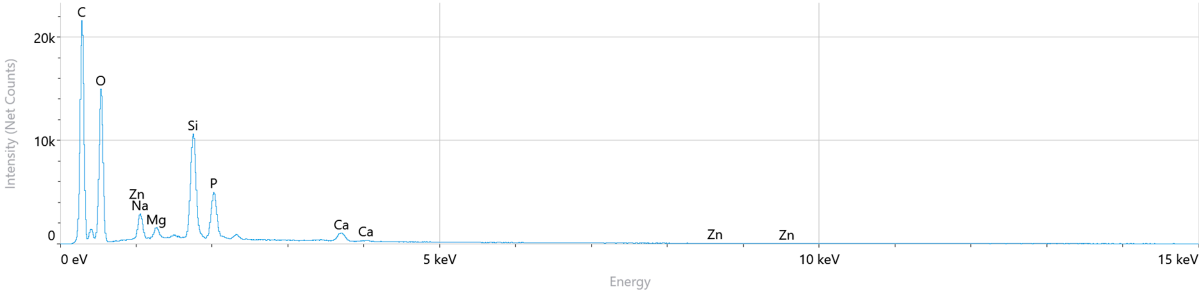

Point 3

Wt. % Error	At. % Error	Net Counts	Wt. %	At. %	Line	Element
0.2	0.2	103 090	33.9	43.9	K	C
0.6	0.7	3 766	4.6	5.1	K	N
0.3	0.3	79 747	41.3	40.0	K	O
0.0	0.0	17 690	2.9	2.0	K	Na
0.0	0.0	7 084	0.9	0.6	K	Mg
0.0	0.0	90 294	10.0	5.5	K	Si
0.1	0.0	37 793	4.4	2.2	K	P
0.0	0.0	9 791	1.9	0.7	K	Ca
0.1	0.0	44	0.1	0.0	K	Zn

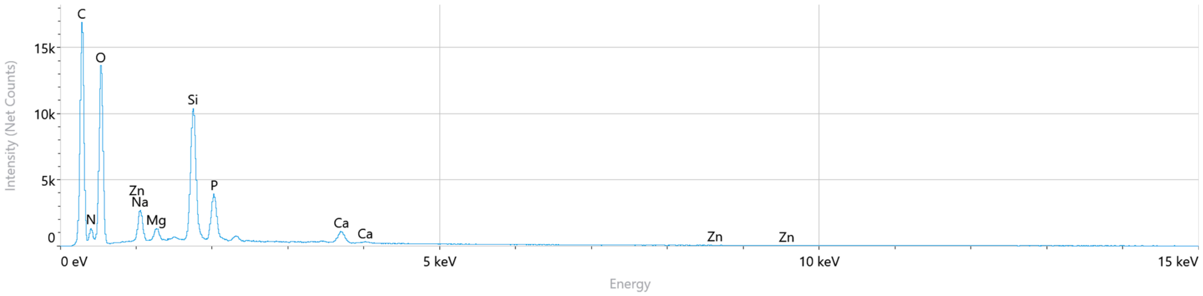

Sample 5 - 0.1 mg/mL ZnO

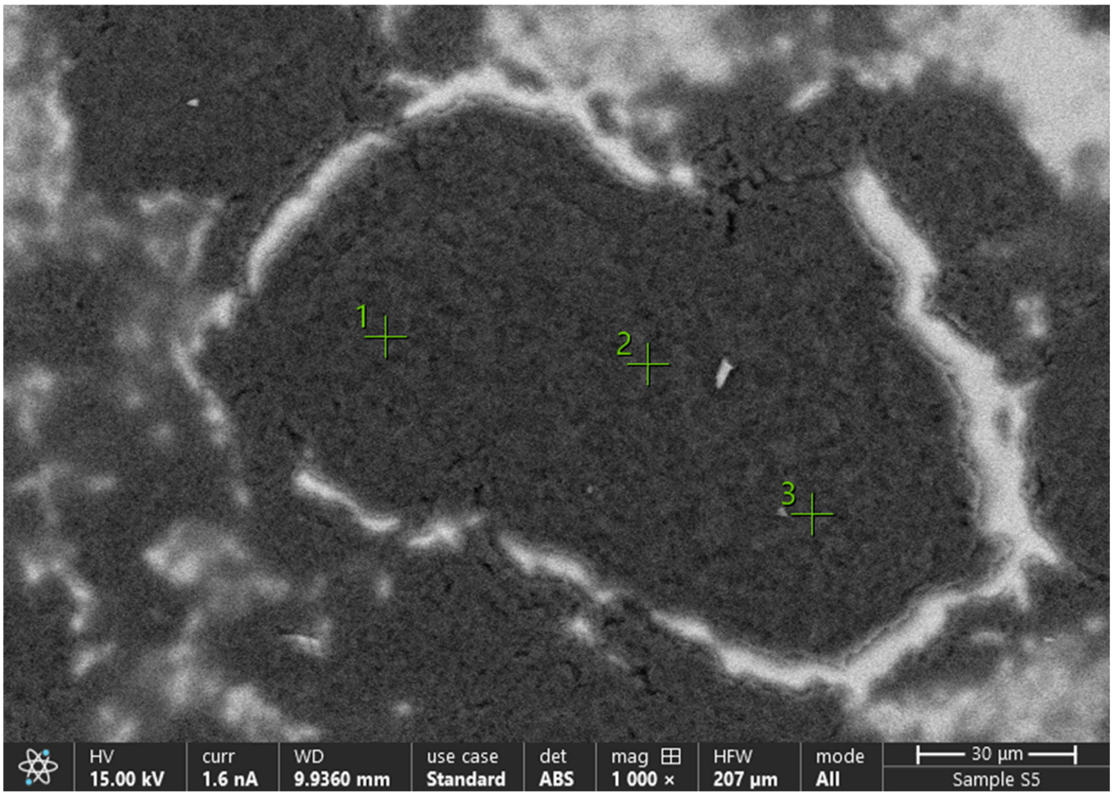

Point 1

Wt. % Error	At. % Error	Net Counts	Wt. %	At. %	Line	Element
0.2	0.2	68 829	32.9	45.6	K	C
0.2	0.2	53 062	34.6	36.1	K	O
0.0	0.0	17 708	3.3	2.4	K	Na
0.0	0.0	6 330	0.9	0.6	K	Mg
0.1	0.0	140 400	18.0	10.6	K	Si
0.1	0.0	22 800	3.3	1.8	K	P
0.1	0.0	30 031	7.0	2.9	K	Ca
0.0	0.0	6	0.0	0.0	K	Zn

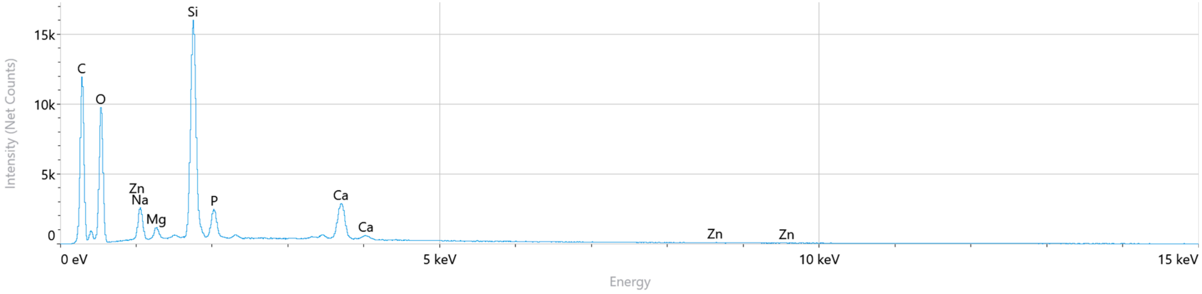

Point 2

Wt. % Error	At. % Error	Net Counts	Wt. %	At. %	Line	Element
0.2	0.2	122 596	33.7	43.9	K	C
0.6	0.7	6 787	7.2	8.0	K	N
0.3	0.3	76 120	36.6	35.8	K	O
0.0	0.0	17 860	2.4	1.7	K	Na
0.0	0.0	6 789	0.7	0.5	K	Mg
0.0	0.0	1 895	0.2	0.1	K	Al
0.0	0.0	132 840	12.1	6.8	K	Si
0.0	0.0	29 758	2.9	1.5	K	P
0.0	0.0	3 337	0.3	0.2	K	S
0.1	0.0	24 140	3.9	1.5	K	Ca
---	---	0	0.0	0.0	K	Zn

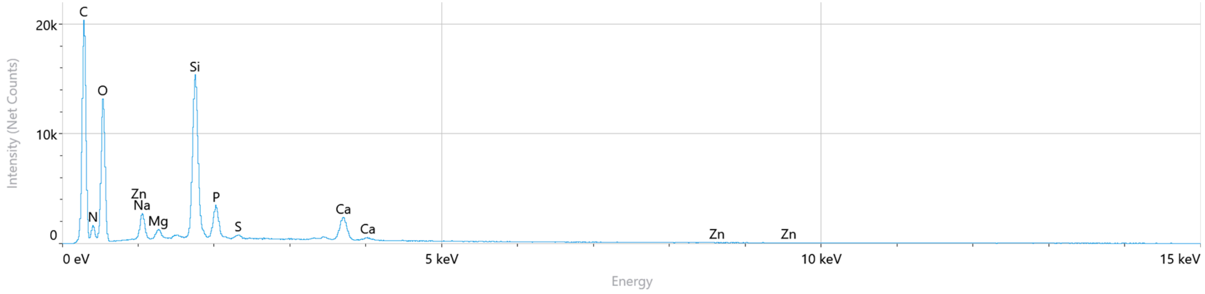

Point 3

Wt. % Error	At. % Error	Net Counts	Wt. %	At. %	Line	Element
0.2	0.2	89 908	32.7	44.0	K	C
0.6	0.7	3 140	4.1	4.7	K	N
0.3	0.3	64 853	35.3	35.8	K	O
0.0	0.0	17 894	2.8	2.0	K	Na
0.0	0.0	6 902	0.9	0.6	K	Mg
0.0	0.0	2 033	0.2	0.1	K	Al
0.1	0.0	147 338	15.6	9.0	K	Si
0.0	0.0	27 249	3.2	1.7	K	P
0.1	0.0	26 757	5.1	2.1	K	Ca
0.1	0.0	81	0.1	0.0	K	Zn

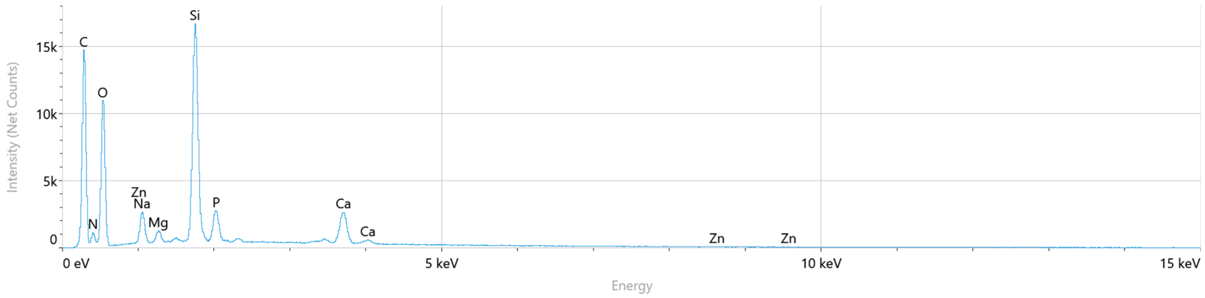

Intensity (Net Counts)
15k
10k
5k
0
C
O
N
Zn
Na
Mg
Si
P
Ca
Ca
Zn
Zn
0 eV
5 keV
10 keV
15 keV
Energy

Sample 6 - 0.1 mg/mL ZnO

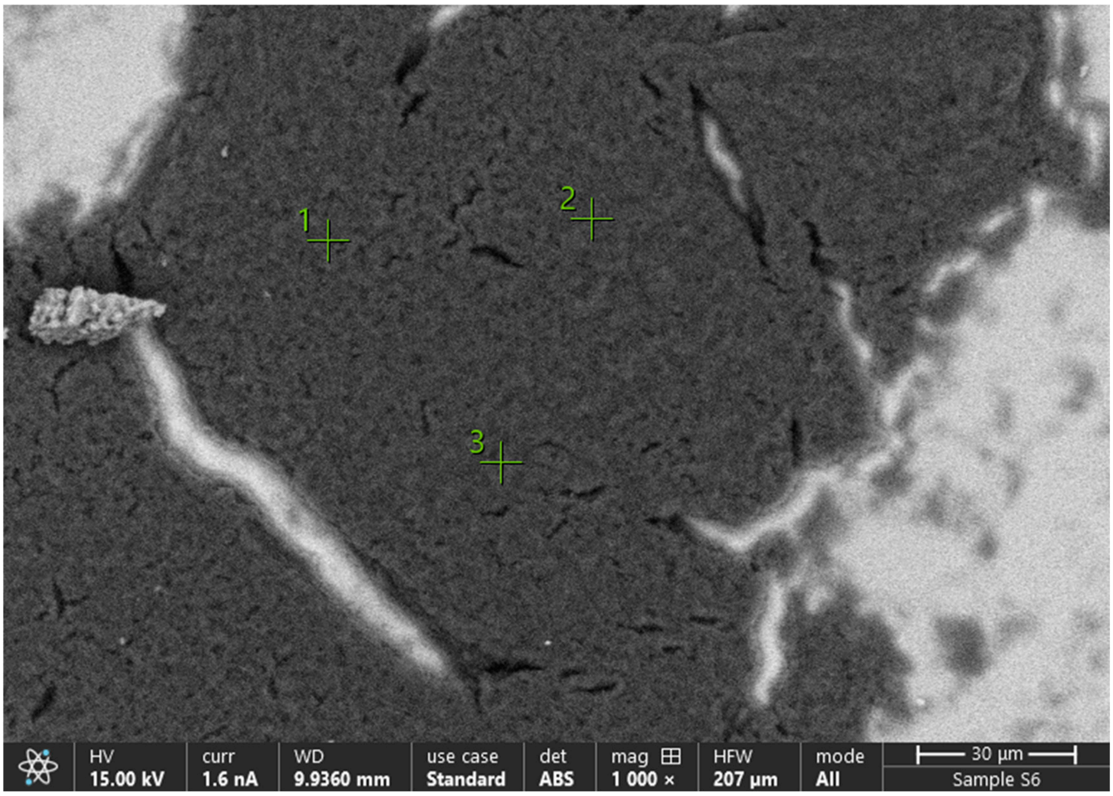

Point 1

Wt. % Error	At. % Error	Net Counts	Wt. %	At. %	Line	Element
0.2	0.2	86 657	28.9	39.5	K	C
0.3	0.4	5 265	5.7	6.6	K	N
0.3	0.3	78 904	37.6	38.4	K	O
0.0	0.0	20 156	2.8	2.0	K	Na
0.0	0.0	8 037	0.9	0.6	K	Mg
0.0	0.0	2 138	0.2	0.1	K	Al
0.1	0.0	157 766	15.0	8.7	K	Si
0.0	0.0	35 415	3.7	1.9	K	P
0.0	0.0	3 883	0.4	0.2	K	S
0.1	0.0	28 069	4.8	2.0	K	Ca
---	---	0	0.0	0.0	K	Zn

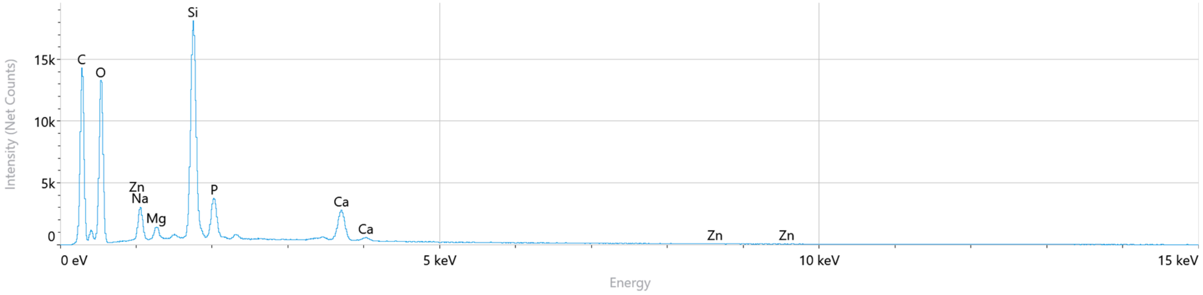

Point 2

Wt. % Error	At. % Error	Net Counts	Wt. %	At. %	Line	Element
0.2	0.2	101 155	32.9	44.0	K	C
0.4	0.4	3 753	4.3	4.9	K	N
0.3	0.3	76 026	36.5	36.6	K	O
0.0	0.0	20 372	2.9	2.0	K	Na
0.0	0.0	8 367	0.9	0.6	K	Mg
0.0	0.0	2 156	0.2	0.1	K	Al
0.1	0.0	143 304	13.6	7.8	K	Si
0.1	0.0	44 919	4.6	2.4	K	P
0.1	0.0	23 256	4.0	1.6	K	Ca
0.1	0.0	64	0.1	0.0	K	Zn

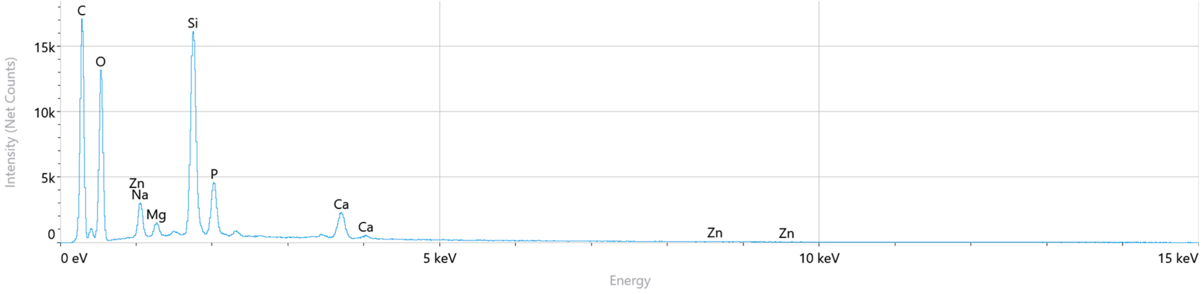

Point 3

Wt. % Error	At. % Error	Net Counts	Wt. %	At. %	Line	Element
0.2	0.2	93 978	30.4	40.9	K	C
0.6	0.6	5 227	5.7	6.5	K	N
0.3	0.3	79 254	38.1	38.4	K	O
0.0	0.0	18 878	2.7	1.9	K	Na
0.0	0.0	8 000	0.9	0.6	K	Mg
0.0	0.0	2 116	0.2	0.1	K	Al
0.1	0.0	136 472	13.2	7.6	K	Si
0.1	0.0	39 401	4.1	2.1	K	P
0.0	0.0	4 033	0.4	0.2	K	S
0.1	0.0	24 107	4.2	1.7	K	Ca
0.1	0.0	89	0.1	0.0	K	Zn

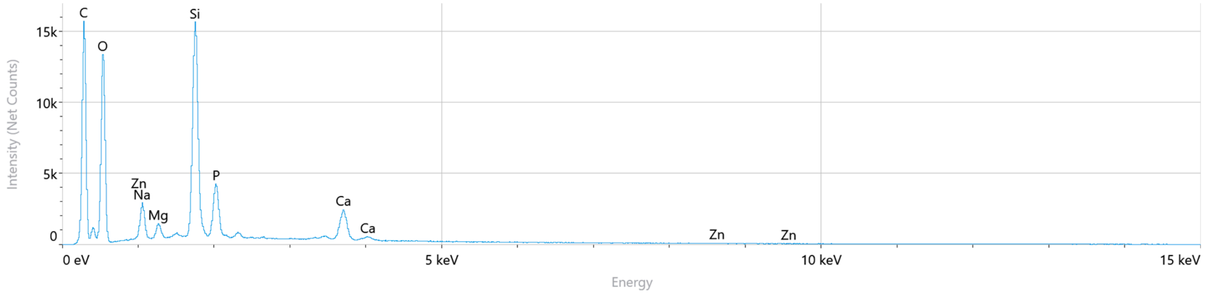

Sample 7 – 0.25 mg/mL ZnO

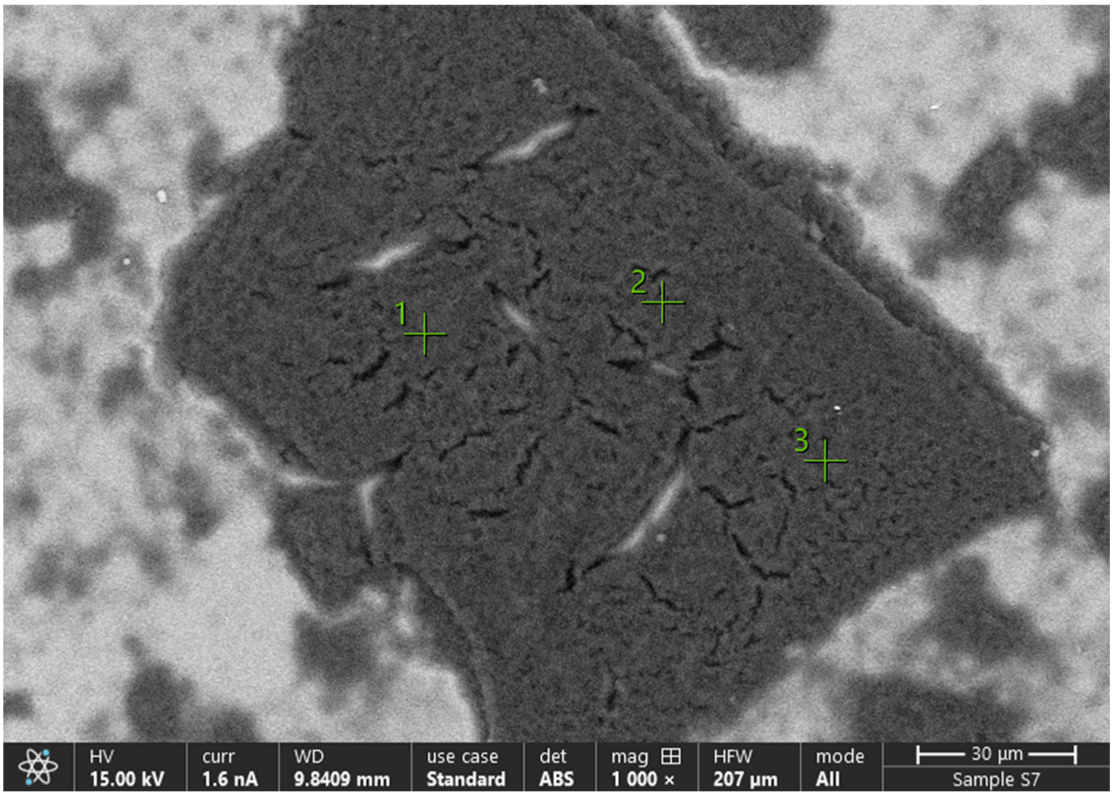

Point 1

Wt. % Error	At. % Error	Net Counts	Wt. %	At. %	Line	Element
0.1	0.2	53 600	25.0	37.6	K	C
0.2	0.2	66 464	32.2	36.1	K	O
0.0	0.0	27 167	3.7	2.9	K	Na
0.0	0.0	10 956	1.2	0.9	K	Mg
0.1	0.1	282 636	27.2	17.4	K	Si
0.0	0.0	21 423	2.5	1.4	K	P
0.0	0.0	3 073	0.3	0.2	K	S
0.1	0.0	42 509	7.5	3.4	K	Ca
0.2	0.0	233	0.4	0.1	K	Zn

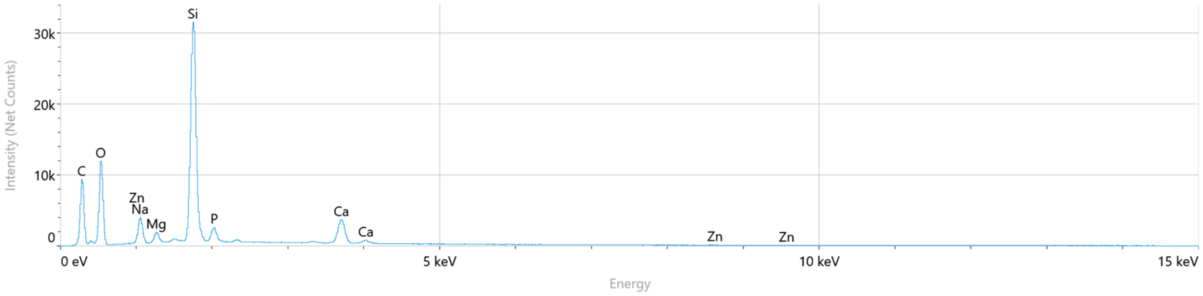

Point 2

Wt. % Error	At. % Error	Net Counts	Wt. %	At. %	Line	Element
0.1	0.2	69 074	27.5	40.6	K	C
0.2	0.2	72 188	31.2	34.4	K	O
0.0	0.0	28 412	3.4	2.6	K	Na
0.0	0.0	12 533	1.2	0.9	K	Mg
0.1	0.1	327 446	27.5	17.3	K	Si
0.0	0.0	16 181	1.6	0.9	K	P
0.0	0.0	2 810	0.3	0.2	K	S
0.0	0.0	45 909	7.1	3.1	K	Ca
0.2	0.0	127	0.2	0.0	K	Zn

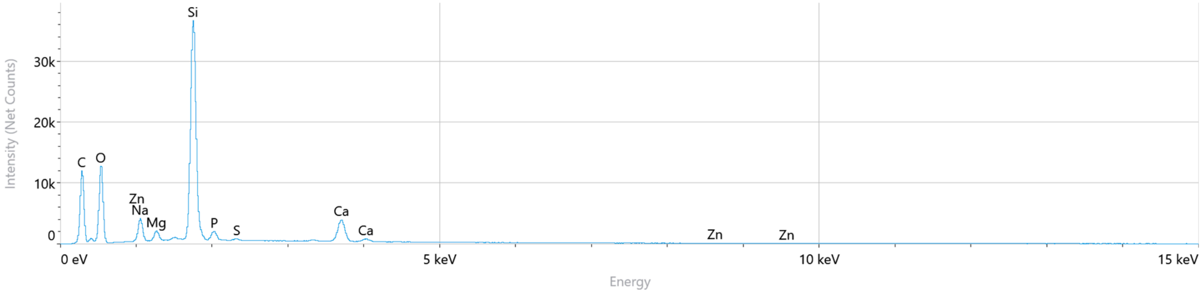

Point 3

Wt. % Error	At. % Error	Net Counts	Wt. %	At. %	Line	Element
0.1	0.2	77 075	26.0	37.9	K	C
0.3	0.4	4 087	3.7	4.7	K	N
0.2	0.2	78 243	30.1	33.0	K	O
0.0	0.0	32 087	3.3	2.5	K	Na
0.0	0.0	14 718	1.2	0.9	K	Mg

Wt. % Error	At. % Error	Net Counts	Wt. %	At. %	Line	Element
0.1	0.1	375 821	27.0	16.9	K	Si
0.0	0.0	19 527	1.7	1.0	K	P
0.0	0.0	3 496	0.3	0.2	K	S
0.0	0.0	49 691	6.6	2.9	K	Ca
0.1	0.0	42	0.1	0.0	K	Zn

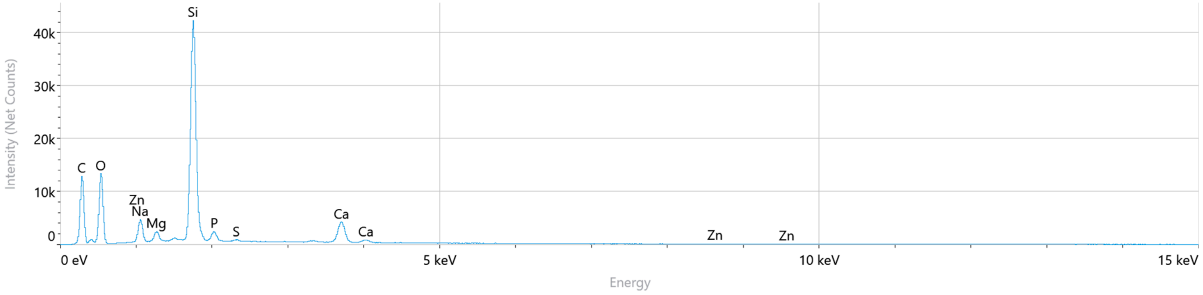

Sample 8– 0.25 mg/mL ZnO

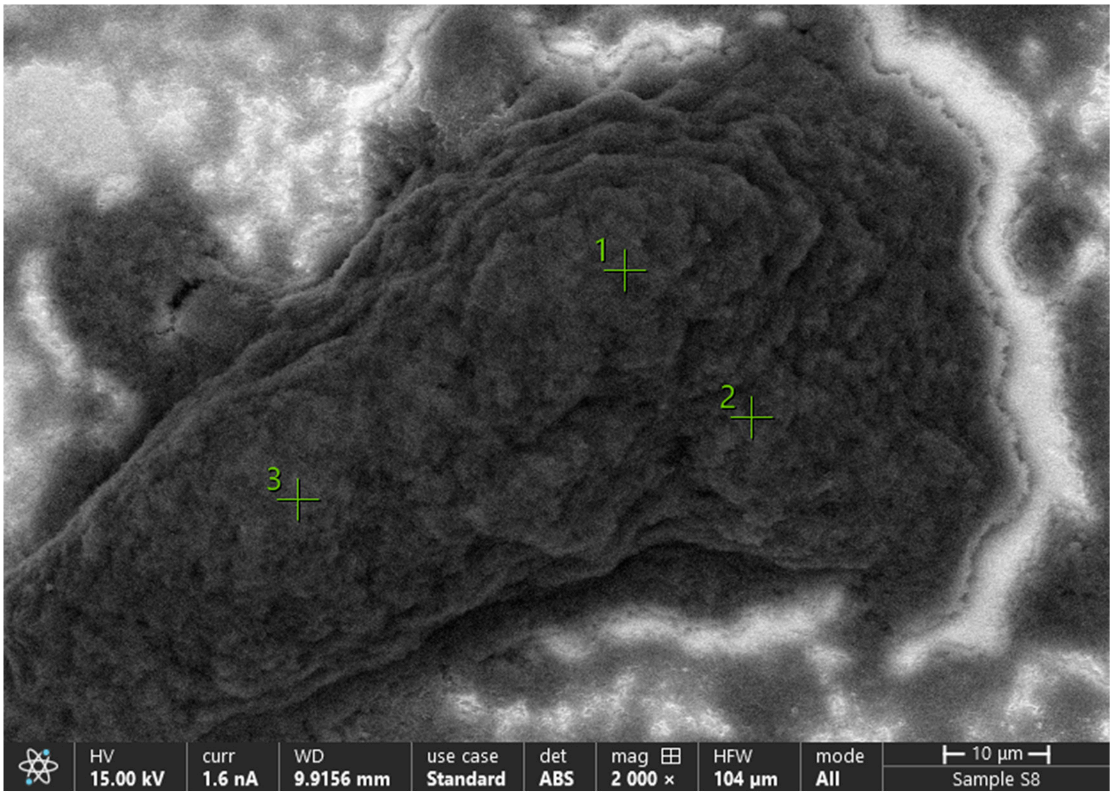

Point 1

Wt. % Error	At. % Error	Net Counts	Wt. %	At. %	Line	Element
0.2	0.3	48 794	32.3	44.8	K	C
0.2	0.3	46 107	35.2	36.4	K	O
0.0	0.0	19 968	4.6	3.3	K	Na
0.0	0.0	6 572	1.2	0.8	K	Mg
0.1	0.1	103 510	16.7	9.9	K	Si
0.1	0.0	30 582	5.5	2.9	K	P
0.0	0.0	5 478	1.0	0.5	K	S
0.1	0.0	11 317	3.3	1.4	K	Ca
0.2	0.0	70	0.2	0.0	K	Zn

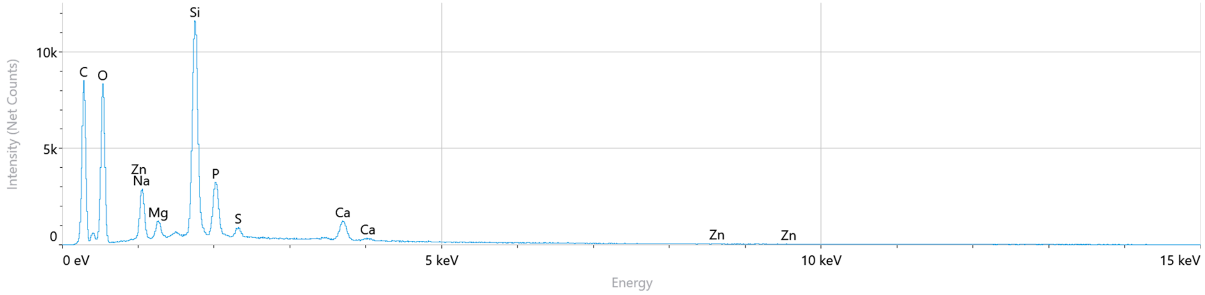

Point 2

Wt. % Error	At. % Error	Net Counts	Wt. %	At. %	Line	Element
0.2	0.3	50 961	32.1	44.1	K	C
0.3	0.3	49 650	37.2	38.1	K	O
0.0	0.0	19 563	4.6	3.3	K	Na
0.0	0.0	6 719	1.3	0.9	K	Mg
0.0	0.0	1 459	0.2	0.2	K	Al
0.1	0.0	102 388	17.0	10.0	K	Si
0.1	0.0	20 837	3.8	2.0	K	P
0.1	0.0	10 857	3.2	1.3	K	Ca
0.2	0.0	211	0.6	0.1	K	Zn

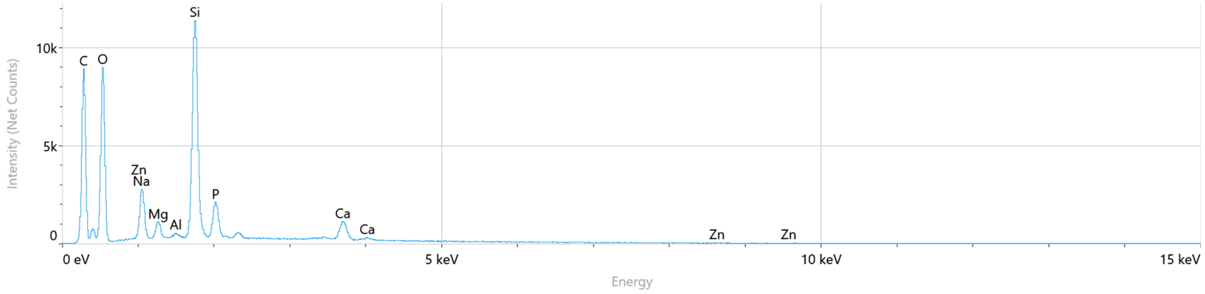

Point 3

Wt. % Error	At. % Error	Net Counts	Wt. %	At. %	Line	Element
0.2	0.3	59 778	34.1	46.3	K	C
0.2	0.2	50 986	35.8	36.5	K	O
0.0	0.0	20 078	4.3	3.1	K	Na
0.0	0.0	7 106	1.2	0.8	K	Mg
0.0	0.0	1 406	0.2	0.1	K	Al

Wt. % Error	At. % Error	Net Counts	Wt. %	At. %	Line	Element
0.1	0.0	103 638	15.5	9.0	K	Si
0.1	0.0	27 985	4.6	2.4	K	P
0.0	0.0	4 565	0.8	0.4	K	S
0.1	0.0	11 484	3.1	1.3	K	Ca
0.2	0.0	166	0.4	0.1	K	Zn

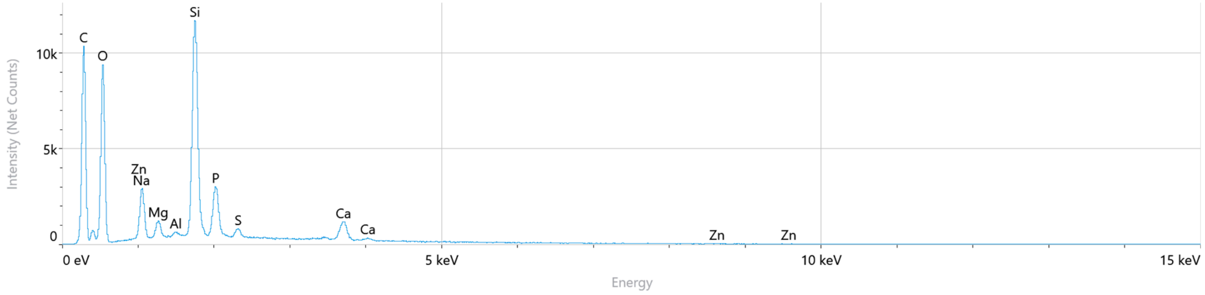

Sample 9 – 0.25 mg/mL ZnO

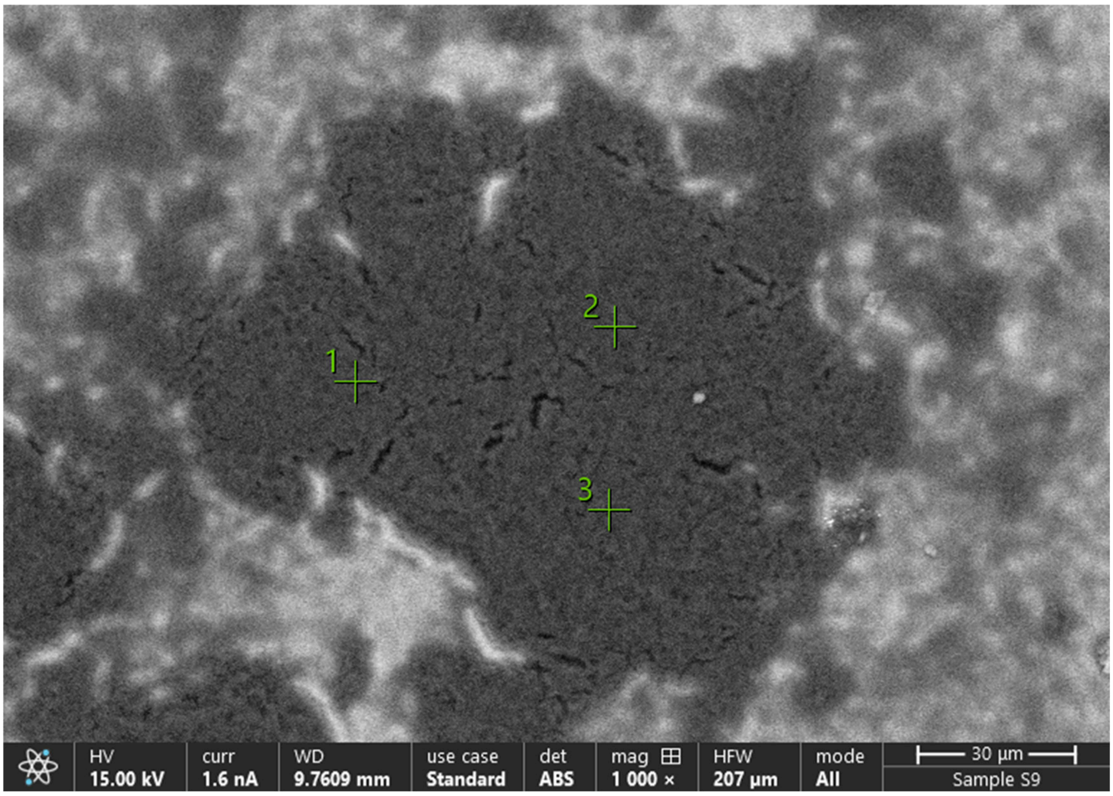

Point 1

Wt. % Error	At. % Error	Net Counts	Wt. %	At. %	Line	Element
0.2	0.2	79 989	31.5	45.9	K	C
0.2	0.2	58 885	25.9	28.3	K	O
0.0	0.0	28 855	3.2	2.4	K	Na
0.0	0.0	14 736	1.3	1.0	K	Mg
0.1	0.1	369 667	29.2	18.2	K	Si
0.0	0.0	19 196	1.9	1.1	K	P
0.0	0.0	3 330	0.3	0.2	K	S
0.1	0.0	44 172	6.6	2.9	K	Ca
0.1	0.0	79	0.1	0.0	K	Zn

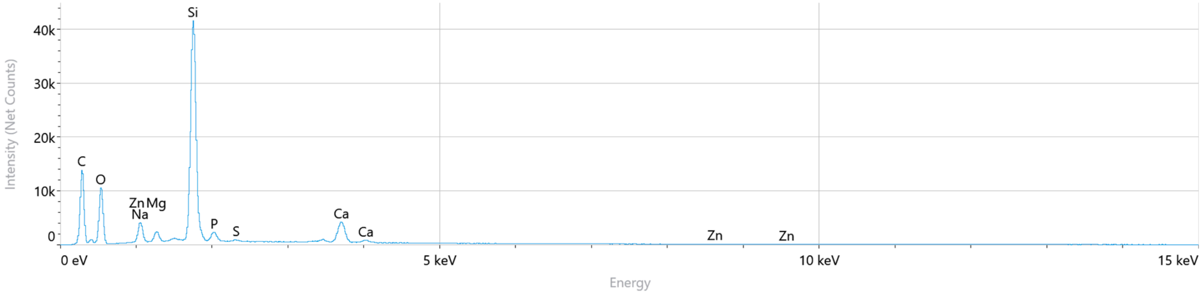

Point 2

Wt. % Error	At. % Error	Net Counts	Wt. %	At. %	Line	Element
0.2	0.2	77 608	31.6	45.8	K	C
0.2	0.2	58 291	27.4	29.8	K	O
0.0	0.0	25 341	3.0	2.3	K	Na
0.0	0.0	11 740	1.1	0.8	K	Mg
0.1	0.1	321 901	27.5	17.0	K	Si
0.0	0.0	19 157	2.0	1.1	K	P
0.0	0.0	3 514	0.4	0.2	K	S
0.1	0.0	43 124	6.9	3.0	K	Ca
0.1	0.0	54	0.1	0.0	K	Zn

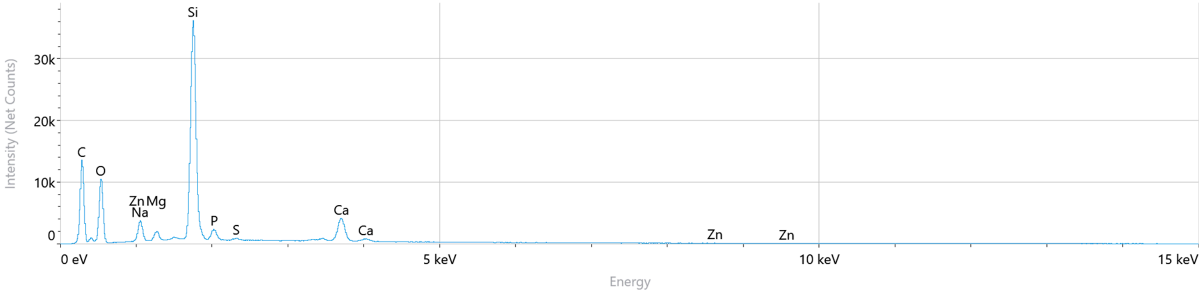

Point 3

Wt. % Error	At. % Error	Net Counts	Wt. %	At. %	Line	Element
0.2	0.2	85 830	30.6	43.6	K	C
0.5	0.6	2 706	2.9	3.6	K	N
0.2	0.2	64 812	29.0	30.9	K	O
0.0	0.0	24 731	2.9	2.1	K	Na
0.0	0.0	10 861	1.0	0.7	K	Mg

Wt. % Error	At. % Error	Net Counts	Wt. %	At. %	Line	Element
0.1	0.1	305 846	24.8	15.1	K	Si
0.0	0.0	23 114	2.2	1.2	K	P
0.1	0.0	41 966	6.3	2.7	K	Ca
0.2	0.1	190	0.3	0.1	K	Zn

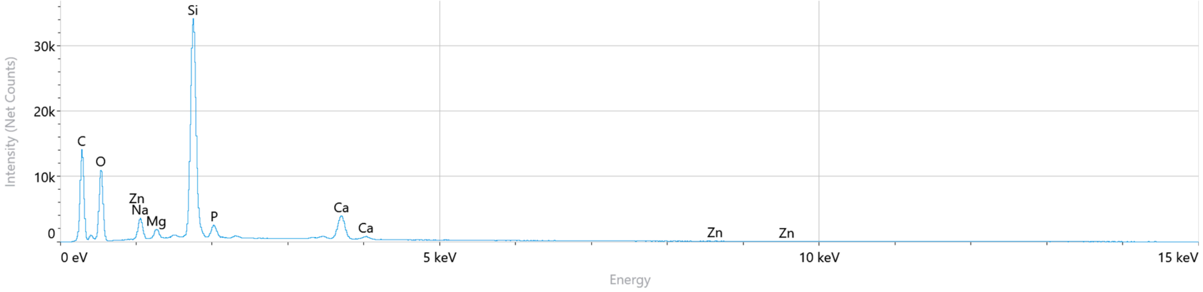

Sample 10– 0.5 mg/mL ZnO

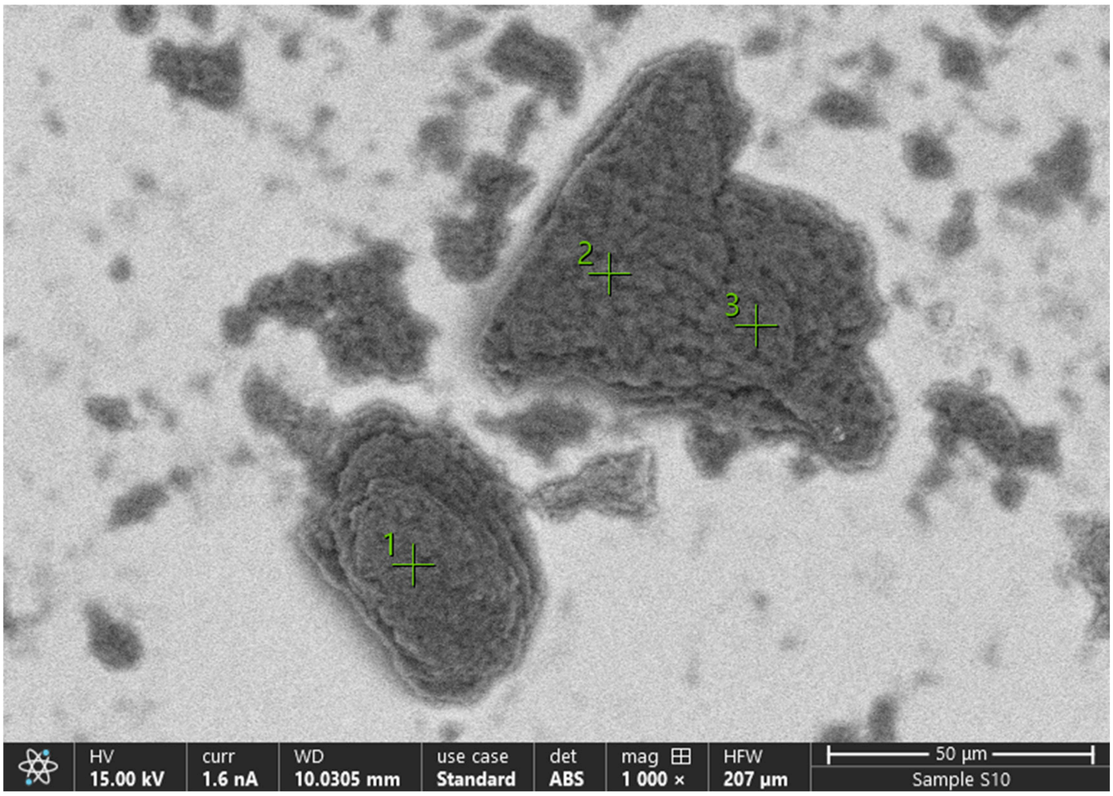

Point 1

Wt. % Error	At. % Error	Net Counts	Wt. %	At. %	Line	Element
0.1	0.2	25 104	15.9	25.5	K	C
0.2	0.3	77 302	37.3	45.1	K	O
0.1	0.0	36 997	7.0	5.9	K	Na
0.0	0.0	10 189	1.7	1.3	K	Mg
0.1	0.1	162 012	21.4	14.7	K	Si
0.1	0.0	33 051	4.9	3.0	K	P
0.0	0.0	5 985	0.9	0.5	K	S
0.1	0.0	18 090	4.1	2.0	K	Ca
0.4	0.1	3 381	6.8	2.0	K	Zn

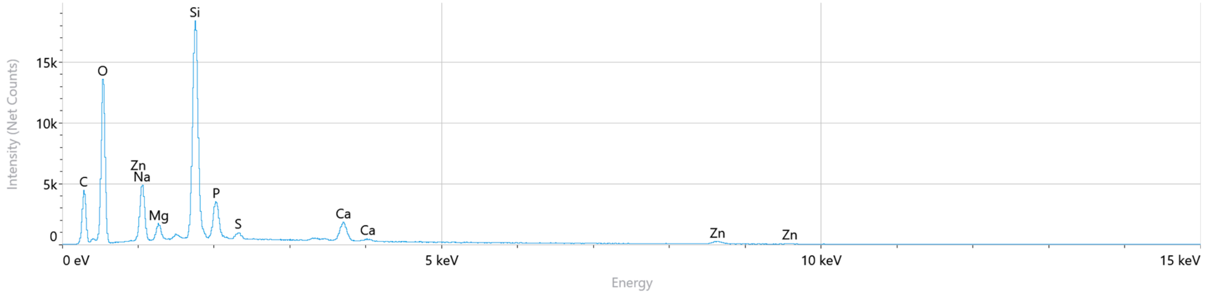

Point 2

Wt. % Error	At. % Error	Net Counts	Wt. %	At. %	Line	Element
0.2	0.2	172 486	36.6	48.9	K	C
0.2	0.2	126 029	36.3	36.6	K	O
0.0	0.0	58 882	5.7	4.0	K	Na
0.0	0.0	10 335	0.8	0.6	K	Mg
0.0	0.0	152 052	10.1	5.8	K	Si
0.0	0.0	54 453	3.8	2.0	K	P
0.0	0.0	8 763	0.6	0.3	K	S
0.0	0.0	17 053	1.9	0.8	K	Ca
0.2	0.1	4 024	4.2	1.0	K	Zn

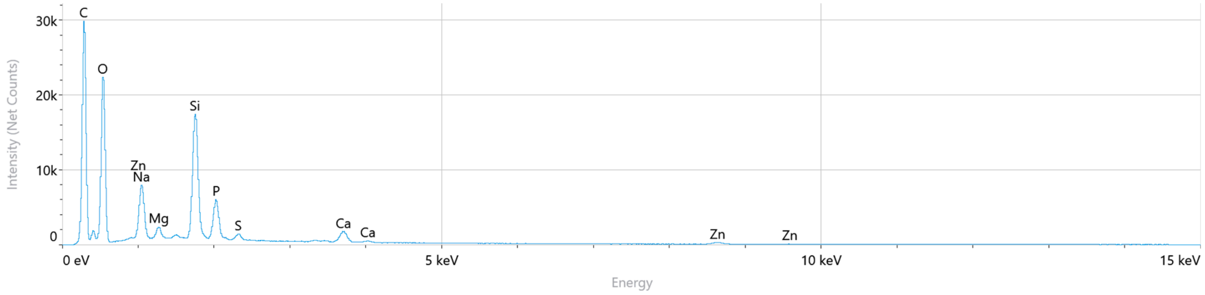

Point 3

Wt. % Error	At. % Error	Net Counts	Wt. %	At. %	Line	Element
0.1	0.2	72 018	28.1	41.0	K	C
0.2	0.2	85 519	34.6	37.7	K	O
0.0	0.0	45 494	6.4	4.8	K	Na
0.0	0.0	10 297	1.2	0.9	K	Mg
0.1	0.0	154 902	15.0	9.4	K	Si

Wt. % Error	At. % Error	Net Counts	Wt. %	At. %	Line	Element
0.1	0.0	50 182	5.2	2.9	K	P
0.0	0.0	7 909	0.8	0.5	K	S
0.1	0.0	17 858	3.0	1.3	K	Ca
0.2	0.1	3 822	5.7	1.5	K	Zn

Sample 11 – 0.5 mg/mL ZnO

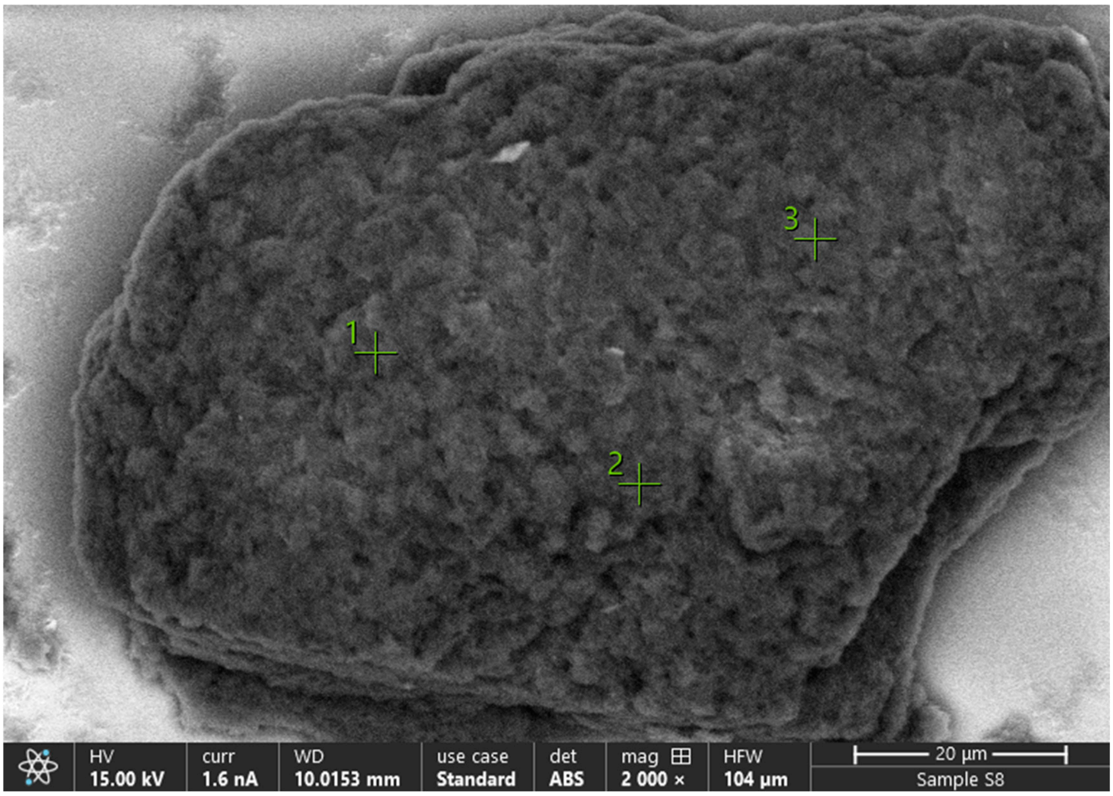

Point 1

Wt. % Error	At. % Error	Net Counts	Wt. %	At. %	Line	Element
0.2	0.2	43 486	24.3	36.1	K	C
0.5	0.7	2 372	3.3	4.2	K	N
0.3	0.3	58 371	34.2	38.1	K	O
0.0	0.0	27 979	5.8	4.5	K	Na
0.0	0.0	6 846	1.2	0.9	K	Mg
0.0	0.0	1 612	0.2	0.2	K	Al
0.1	0.0	104 490	14.8	9.4	K	Si
0.1	0.0	30 835	4.6	2.7	K	P
0.0	0.0	5 064	0.8	0.4	K	S
0.1	0.0	12 872	3.1	1.4	K	Ca
0.4	0.1	3 608	7.7	2.1	K	Zn

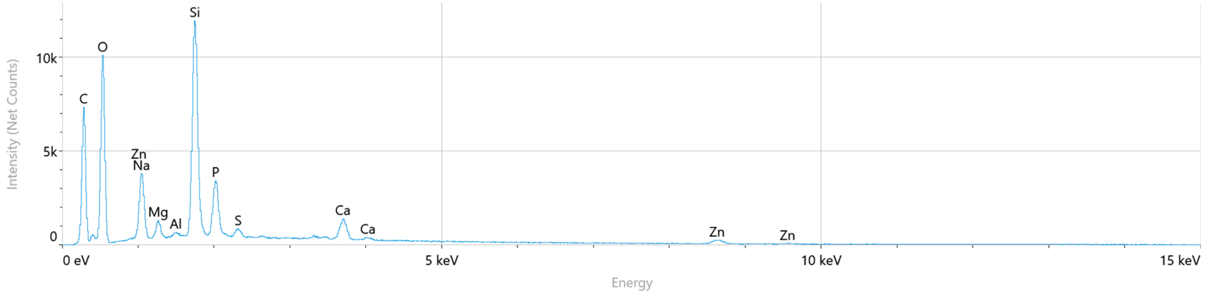

Point 2

Wt. % Error	At. % Error	Net Counts	Wt. %	At. %	Line	Element
0.2	0.2	82 435	27.9	38.7	K	C
0.5	0.6	6 749	6.7	8.0	K	N
0.2	0.3	79 804	36.4	37.8	K	O
0.0	0.0	35 654	5.5	4.0	K	Na
0.0	0.0	6 846	0.9	0.6	K	Mg
0.0	0.0	1 935	0.2	0.1	K	Al
0.0	0.0	100 793	10.5	6.2	K	Si
0.0	0.0	36 036	3.9	2.1	K	P
0.0	0.0	5 882	0.6	0.3	K	S
0.0	0.0	12 482	2.2	0.9	K	Ca
0.3	0.1	3 269	5.2	1.3	K	Zn

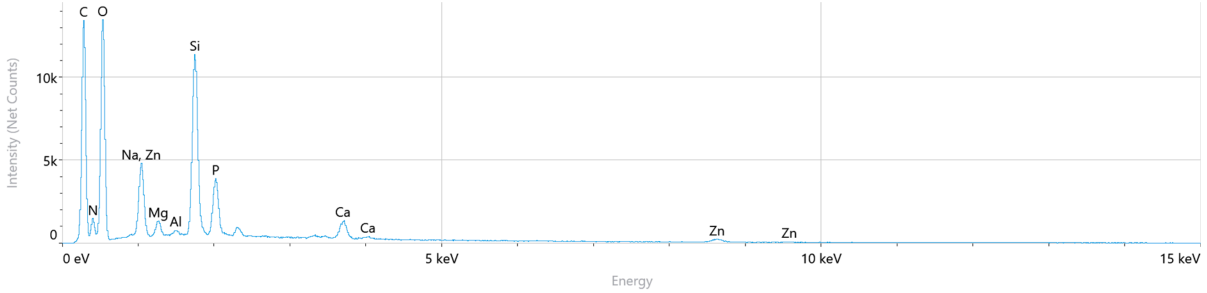

Point 3

Wt. % Error	At. % Error	Net Counts	Wt. %	At. %	Line	Element
0.2	0.2	113 264	30.9	42.6	K	C
0.5	0.6	6 677	5.7	6.8	K	N
0.2	0.2	89 578	34.0	35.1	K	O
0.0	0.0	48 735	6.0	4.3	K	Na
0.0	0.0	7 798	0.8	0.6	K	Mg
0.0	0.0	2 500	0.2	0.1	K	Al
0.0	0.0	106 817	9.0	5.3	K	Si
0.1	0.0	53 572	4.6	2.5	K	P
0.0	0.0	8 924	0.8	0.4	K	S
0.0	0.0	14 107	2.0	0.8	K	Ca
0.3	0.1	4 597	6.0	1.5	K	Zn

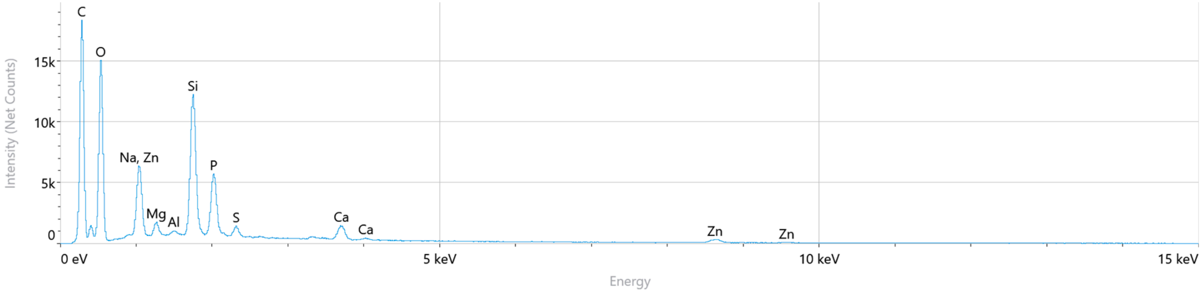

Sample 12 – 0.5 mg/mL ZnO

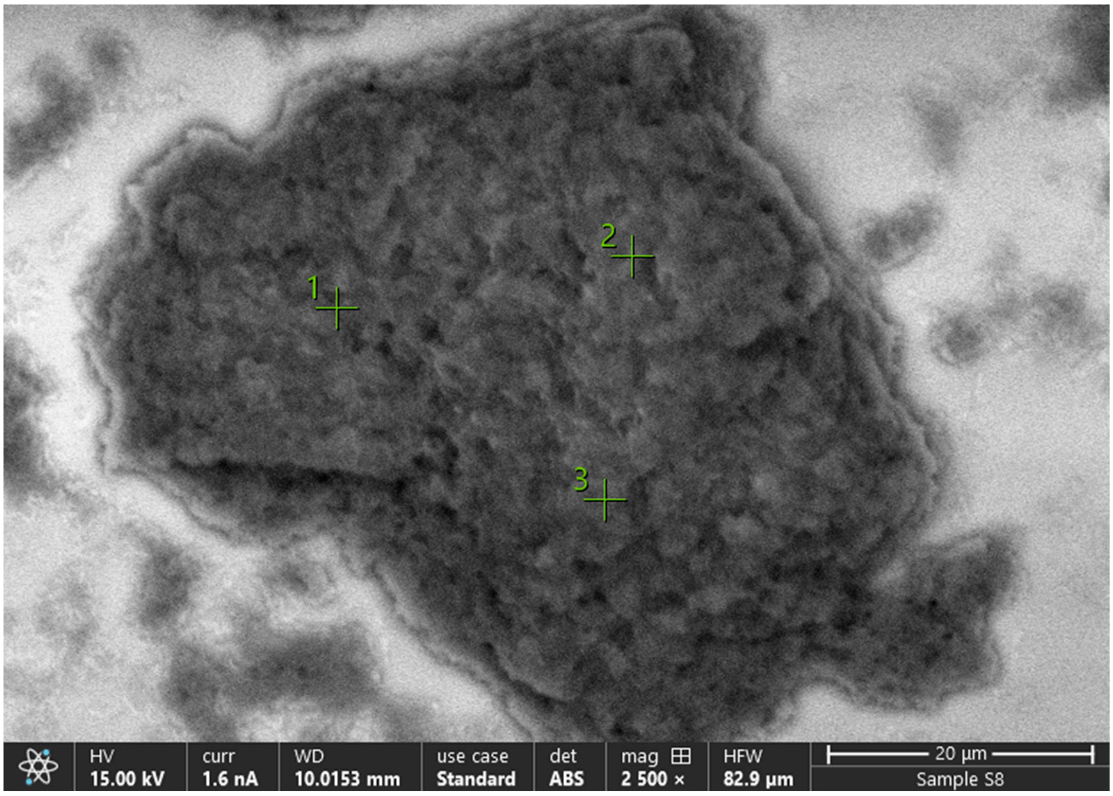

Point 1

Wt. % Error	At. % Error	Net Counts	Wt. %	At. %	Line	Element
0.1	0.2	37 989	20.6	32.2	K	C
0.2	0.2	66 672	33.6	39.7	K	O
0.0	0.0	33 345	5.5	4.5	K	Na
0.0	0.0	9 289	1.3	1.0	K	Mg
0.1	0.1	193 918	22.2	14.8	K	Si
0.1	0.0	36 129	4.7	2.8	K	P
0.0	0.0	5 844	0.8	0.4	K	S
0.0	0.0	1 329	0.2	0.1	K	Cl
0.0	0.0	4 061	0.7	0.4	K	K
0.1	0.0	30 992	6.3	2.9	K	Ca
0.4	0.1	2 283	4.1	1.2	K	Zn

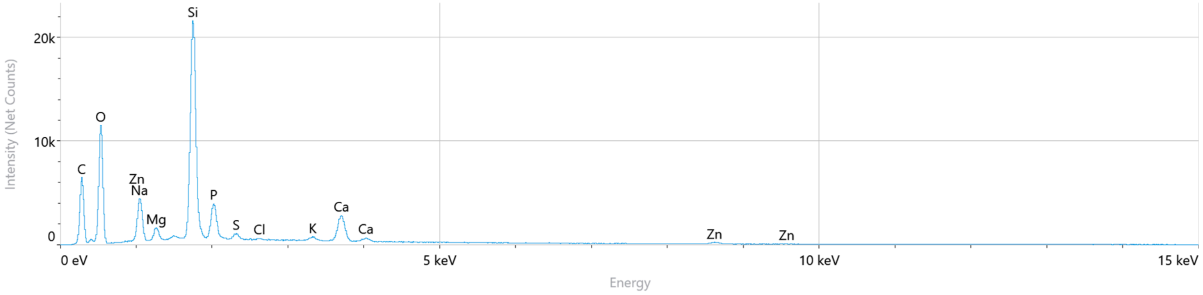

Point 2

Wt. % Error	At. % Error	Net Counts	Wt. %	At. %	Line	Element
0.1	0.2	52 990	24.7	37.0	K	C
0.2	0.2	71 125	34.4	38.7	K	O
0.0	0.0	34 937	5.4	4.2	K	Na
0.0	0.0	9 714	1.3	0.9	K	Mg
0.0	0.0	1 735	0.2	0.1	K	Al
0.1	0.1	182 167	19.6	12.5	K	Si
0.1	0.0	34 018	4.1	2.4	K	P
0.0	0.0	6 167	0.7	0.4	K	S
0.0	0.0	1 524	0.2	0.1	K	Cl
0.0	0.0	3 587	0.6	0.3	K	K
0.1	0.0	30 218	5.7	2.6	K	Ca
0.4	0.1	1 793	3.1	0.8	K	Zn

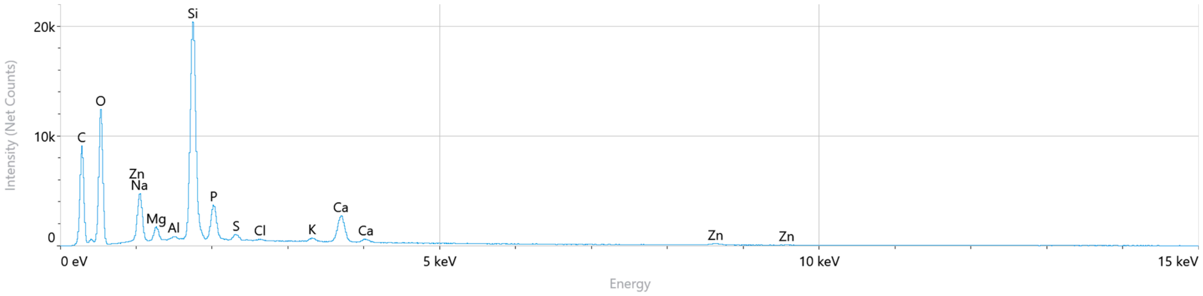

Point 3

Wt. % Error	At. % Error	Net Counts	Wt. %	At. %	Line	Element
0.2	0.2	88 388	30.7	43.5	K	C
0.2	0.2	85 513	36.0	37.9	K	O
0.0	0.0	40 389	5.5	4.0	K	Na
0.0	0.0	9 526	1.1	0.7	K	Mg
0.0	0.0	2 192	0.2	0.1	K	Al
0.1	0.0	156 283	14.7	8.8	K	Si
0.0	0.0	37 100	3.7	2.0	K	P
0.0	0.0	7 599	0.8	0.4	K	S
0.0	0.0	1 441	0.2	0.1	K	Cl
0.0	0.0	3 722	0.5	0.2	K	K
0.1	0.0	25 163	4.1	1.7	K	Ca
0.3	0.1	1 666	2.5	0.6	K	Zn

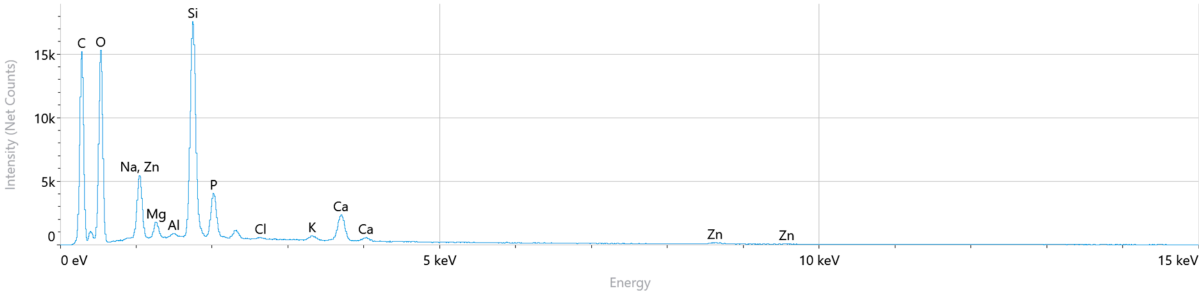

Sample 13 – 1 mg/mL ZnO

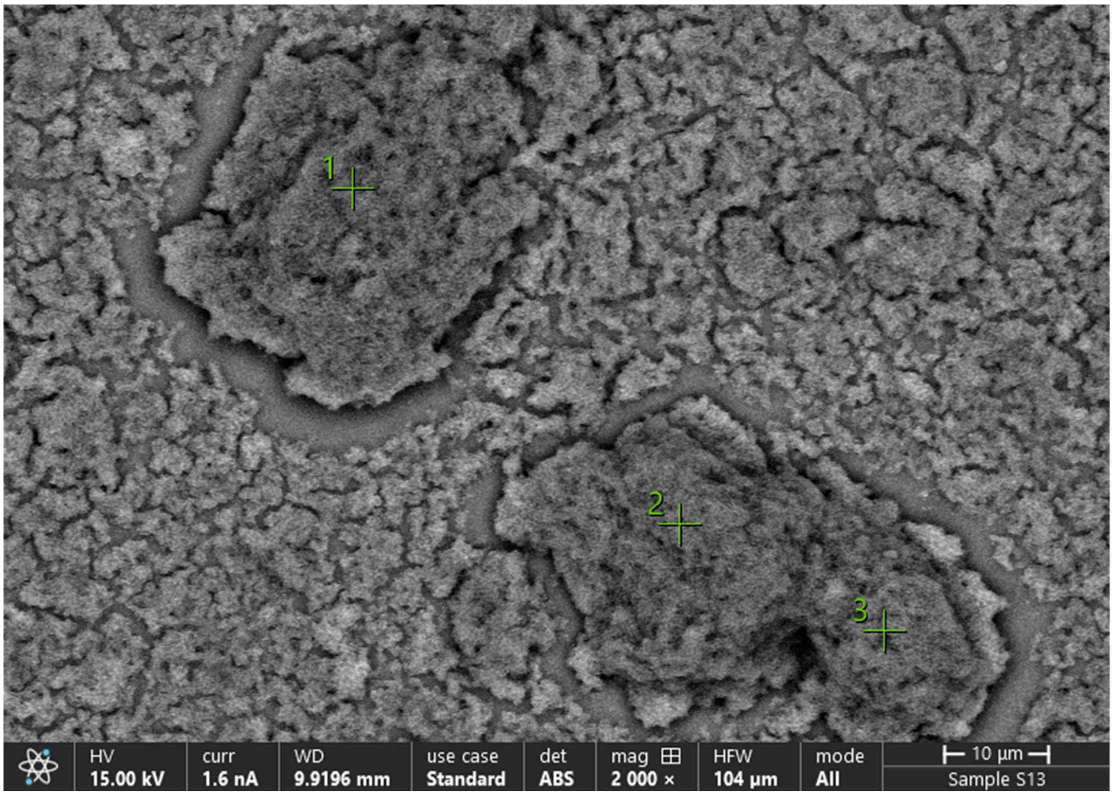

Point 1

Wt. % Error	At. % Error	Net Counts	Wt. %	At. %	Line	Element
0.1	0.2	35 933	12.5	27.4	K	C
0.3	0.5	4 688	2.9	5.5	K	N
0.1	0.2	78 621	21.1	34.6	K	O
0.0	0.0	2 688	0.4	0.4	K	Mg
0.0	0.0	2 089	0.2	0.2	K	Al
0.1	0.1	75 955	7.3	6.8	K	Si
0.1	0.0	56 112	5.0	4.2	K	P
0.0	0.0	5 314	0.5	0.4	K	S
0.0	0.0	12 574	1.6	1.0	K	Ca
0.5	0.2	45 423	48.5	19.5	K	Zn

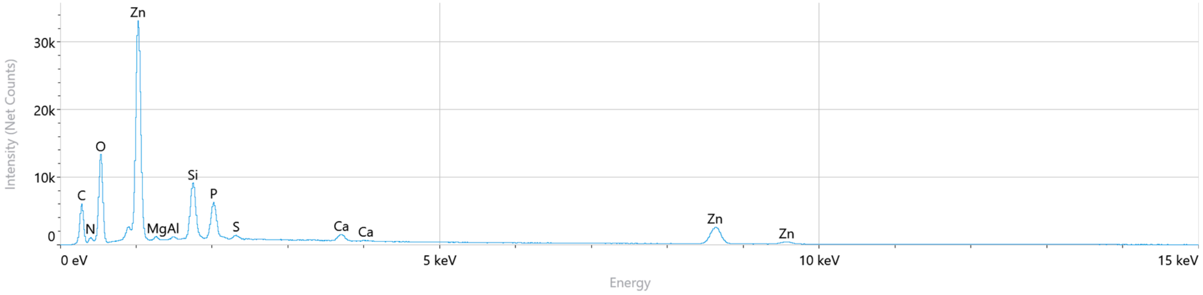

Point 2

Wt. % Error	At. % Error	Net Counts	Wt. %	At. %	Line	Element
0.1	0.2	46 777	12.2	26.9	K	C
0.2	0.4	6 746	3.1	5.9	K	N
0.1	0.2	106 501	21.5	35.6	K	O
0.0	0.0	3 065	0.4	0.4	K	Mg
0.0	0.0	2 871	0.2	0.2	K	Al
0.0	0.0	79 117	5.9	5.6	K	Si
0.1	0.0	67 559	4.6	3.9	K	P
0.0	0.0	5 566	0.4	0.3	K	S
0.0	0.0	13 137	1.2	0.8	K	Ca
0.5	0.2	61 722	50.5	20.4	K	Zn

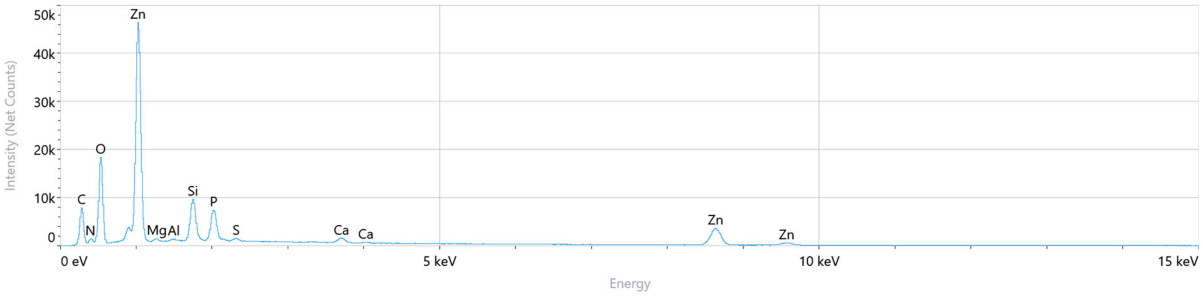

Point 3

Wt. % Error	At. % Error	Net Counts	Wt. %	At. %	Line	Element
0.1	0.2	27 978	11.3	27.9	K	C
0.1	0.2	59 933	16.6	30.7	K	O
0.0	0.0	3 138	0.5	0.6	K	Mg
0.1	0.1	77 657	8.3	8.8	K	Si

Wt. % Error	At. % Error	Net Counts	Wt. %	At. %	Line	Element
0.1	0.1	53 294	5.2	5.0	K	P
0.0	0.0	4 897	0.5	0.4	K	S
0.0	0.0	12 706	1.7	1.3	K	Ca
0.6	0.3	49 231	55.9	25.3	K	Zn

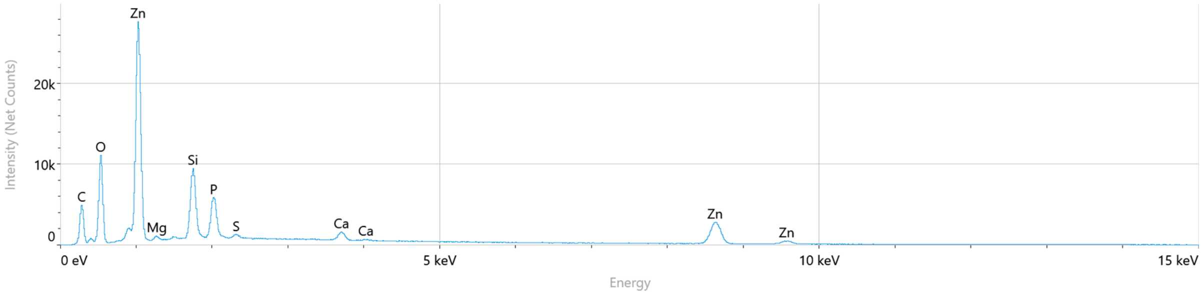

Sample 14 – 1 mg/mL ZnO

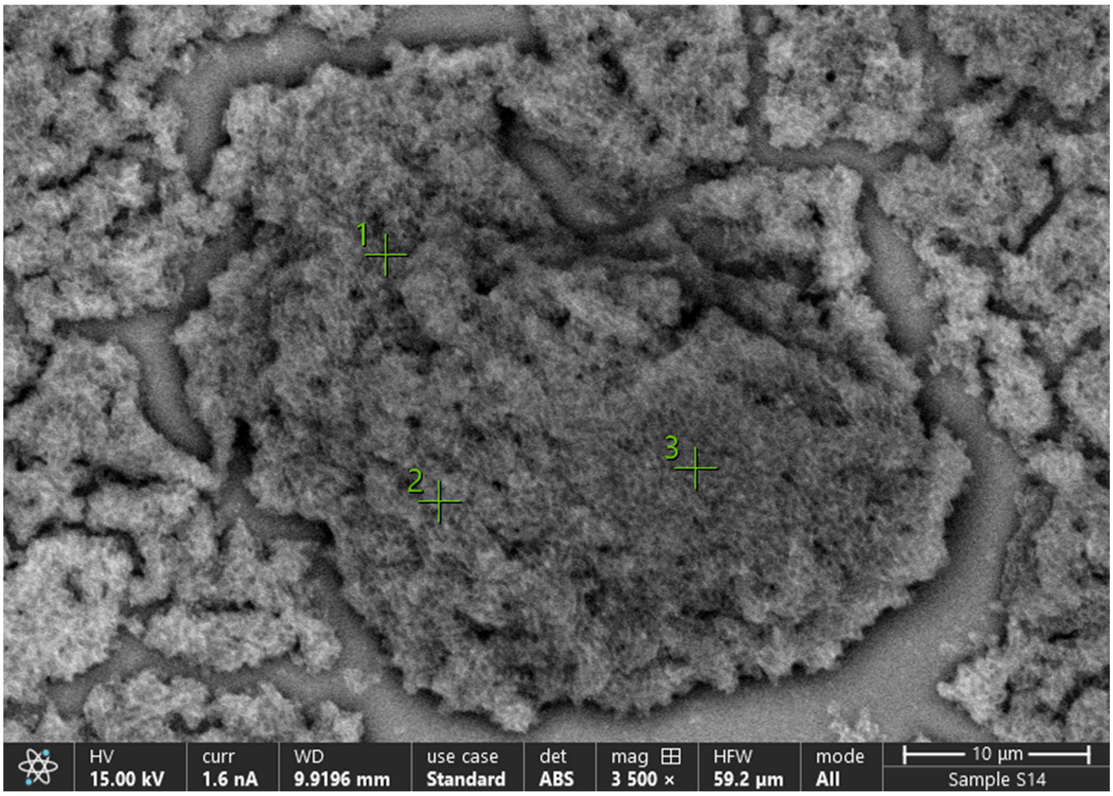

Point 1

Wt. % Error	At. % Error	Net Counts	Wt. %	At. %	Line	Element
0.1	0.2	71 166	14.8	28.7	K	C
0.2	0.4	11 474	4.7	7.8	K	N
0.2	0.2	150 361	27.9	41.0	K	O
0.0	0.0	3 547	0.3	0.2	K	Al
0.0	0.0	58 271	3.8	3.1	K	Si
0.0	0.0	77 096	4.5	3.4	K	P
0.0	0.0	9 568	0.8	0.5	K	Ca
0.4	0.1	56 348	42.2	15.1	K	Zn
0.1	0.0	10 255	1.0	0.2	L	Mo

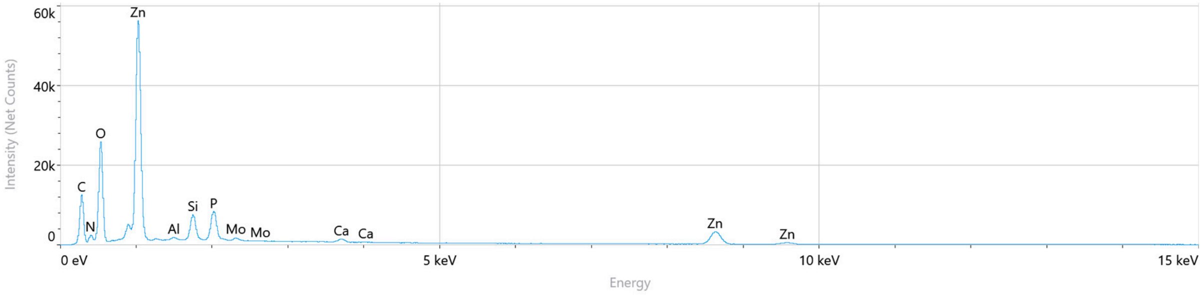

Point 2

Wt. % Error	At. % Error	Net Counts	Wt. %	At. %	Line	Element
0.1	0.3	19 239	8.2	23.3	K	C
0.1	0.2	46 208	12.9	27.5	K	O
0.0	0.1	2 098	0.4	0.5	K	Mg
0.0	0.0	1 594	0.2	0.3	K	Al
0.1	0.1	58 411	6.8	8.3	K	Si
0.1	0.1	41 019	4.3	4.7	K	P
0.0	0.0	9 045	1.2	1.1	K	Ca
0.6	0.3	56 291	65.0	33.9	K	Zn
0.1	0.0	6 213	1.0	0.4	L	Mo

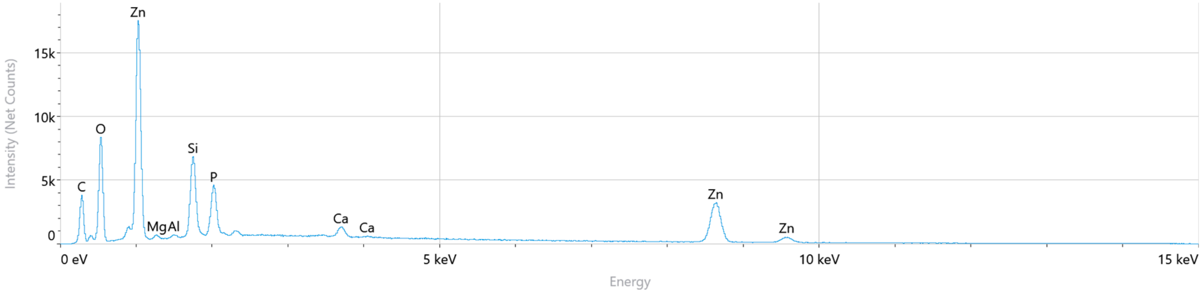

Point 3

Wt. % Error	At. % Error	Net Counts	Wt. %	At. %	Line	Element
0.1	0.3	20 330	8.5	24.0	K	C
0.1	0.2	46 804	13.0	27.5	K	O
0.0	0.1	2 403	0.4	0.6	K	Mg
0.0	0.1	1 502	0.2	0.3	K	Al
0.1	0.1	58 310	6.7	8.1	K	Si

Wt. % Error	At. % Error	Net Counts	Wt. %	At. %	Line	Element
0.1	0.1	43 783	4.5	4.9	K	P
0.0	0.0	9 735	1.3	1.1	K	Ca
0.6	0.3	55 850	64.2	33.1	K	Zn
0.1	0.0	7 564	1.2	0.4	L	Mo

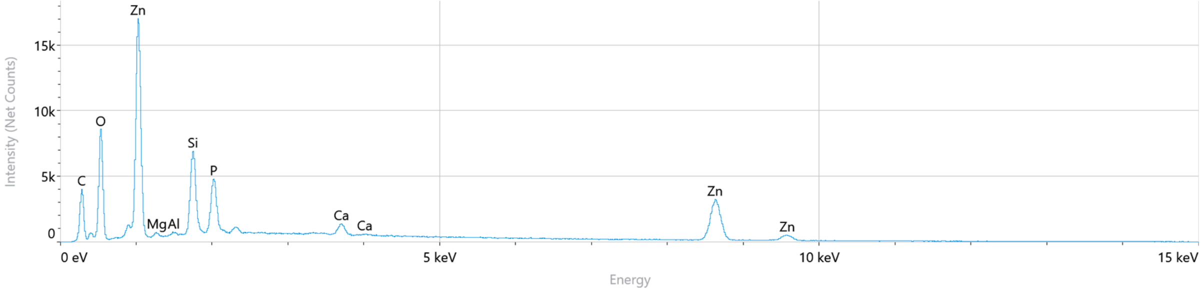

Sample 15 – 1 mg/mL ZnO

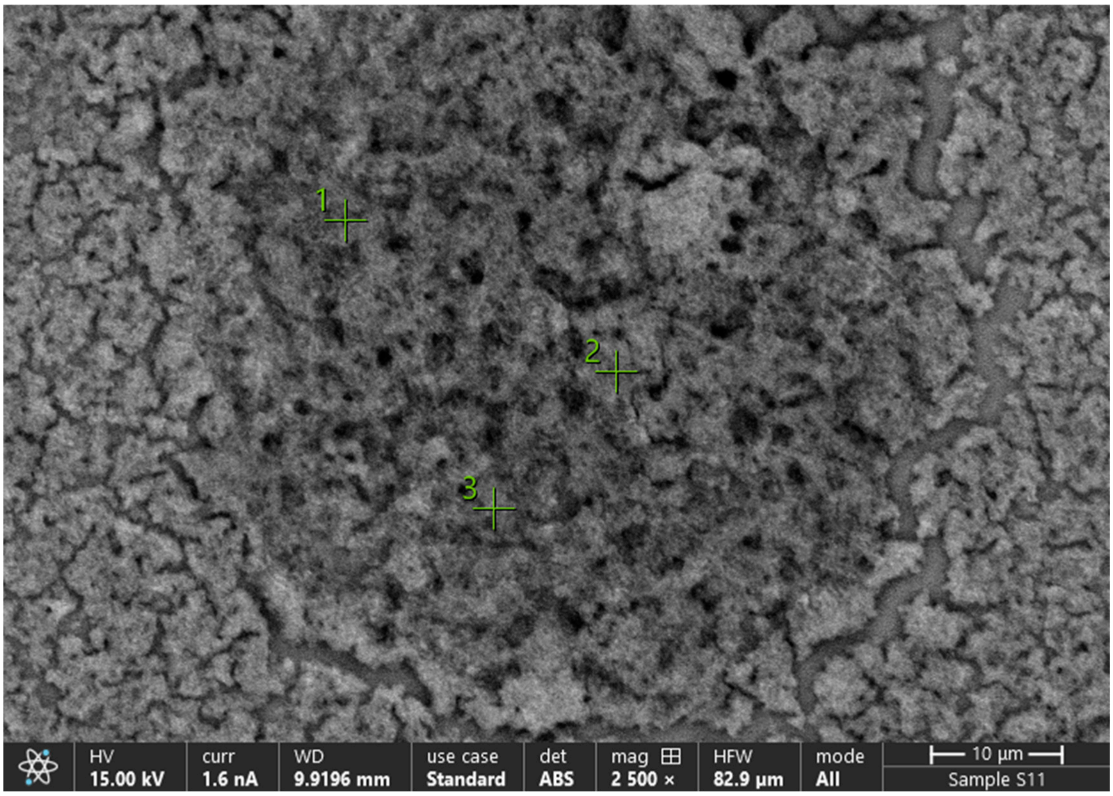

Point 1

Wt. % Error	At. % Error	Net Counts	Wt. %	At. %	Line	Element
0.1	0.2	47 533	12.7	26.2	K	C
0.2	0.4	4 290	2.1	3.7	K	N
0.1	0.2	111 270	22.7	34.9	K	O
0.0	0.0	9 227	0.9	0.9	K	Mg
0.0	0.0	4 105	0.3	0.3	K	Al
0.1	0.1	274 880	17.8	15.7	K	Si
0.0	0.0	37 836	2.5	2.0	K	P
0.0	0.0	36 943	3.4	2.1	K	Ca
0.4	0.1	47 937	37.6	14.2	K	Zn

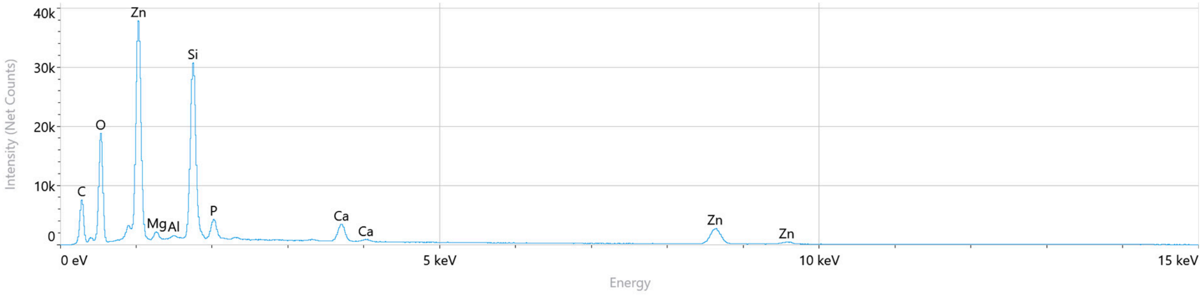

Point 2

Wt. % Error	At. % Error	Net Counts	Wt. %	At. %	Line	Element
0.1	0.2	41 565	12.2	25.3	K	C
0.2	0.4	5 498	2.9	5.2	K	N
0.1	0.2	99 538	22.7	35.2	K	O
0.0	0.0	6 971	0.7	0.8	K	Mg
0.0	0.0	3 515	0.3	0.3	K	Al
0.1	0.1	210 891	15.4	13.7	K	Si
0.0	0.0	39 560	2.9	2.3	K	P
0.0	0.0	4 431	0.3	0.2	K	S
0.0	0.0	1 858	0.2	0.1	K	K
0.0	0.0	29 870	3.0	1.9	K	Ca
0.4	0.2	45 043	39.4	15.0	K	Zn

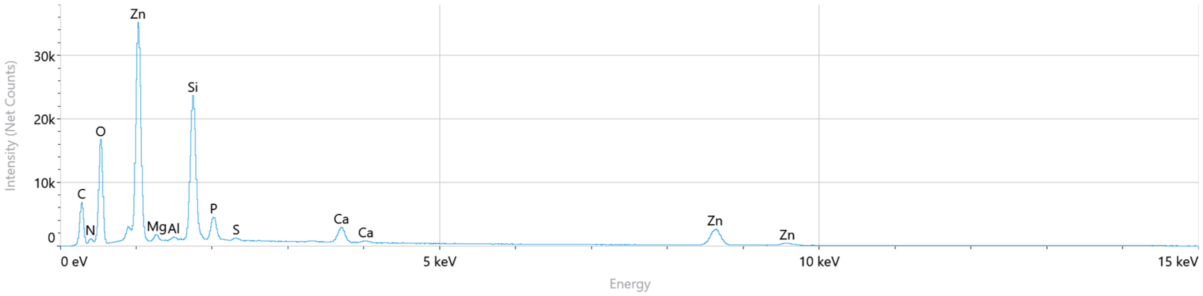

Point 3

Wt. % Error	At. % Error	Net Counts	Wt. %	At. %	Line	Element
0.1	0.2	57 197	13.6	25.6	K	C
0.2	0.4	4 364	1.9	3.0	K	N
0.1	0.2	134 603	24.2	34.0	K	O

Wt. % Error	At. % Error	Net Counts	Wt. %	At. %	Line	Element
0.4	0.4	86 433	7.1	6.9	K	Na
0.0	0.0	16 708	1.2	1.2	K	Mg
0.0	0.0	5 965	0.4	0.3	K	Al
0.1	0.1	387 122	20.8	16.8	K	Si
0.0	0.0	33 244	1.9	1.4	K	P
0.0	0.0	45 323	3.6	2.0	K	Ca
0.3	0.1	36 299	25.3	8.8	K	Zn

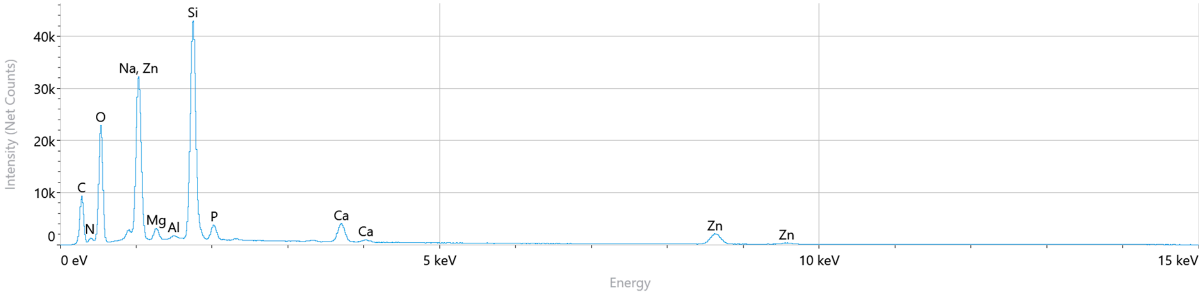

Supplement: Supplementary file 1 [file biomolecules-15-01660-s001.zip › biomolecules-3982741-supplementary.pdf]
